# Metal-free synthesis of tricyclic benzofuro[2,3-*c*]pyridin-3-ol derivatives, characterization, and photoluminescence properties

**DOI:** 10.1039/d5ra05420f

**Published:** 2025-09-04

**Authors:** Surbhi Mahender Saini, Sandeep Chandrashekharappa

**Affiliations:** a Department of Medicinal Chemistry, National Institute of Pharmaceutical Education and Research-Raebareli (NIPER-R) Lucknow UP 226002 India c.sandeep@niperraebareli.edu.in c.sandeep@niperrbl.ac.in +91-522-2975587 +91-522-2499703

## Abstract

This paper presents a metal-free synthetic protocol for assembling novel benzofuro[2,3-*c*]pyridin-3-ols (BFPYOLs) using 2,3-disubstituted benzofuran derivatives with good yield. The method's advantages include the absence of an expensive metal catalyst, organic ligands, and easily accessible starting materials. The photophysical properties of the synthesized BFPYOLs are investigated, revealing that the largest *λ*_abs_ is displayed by compound 7g at 389 nm, while the largest *λ*_em_ is observed in compound 7i at 494 nm in DMSO solvent. This highlights the significant impact of substituents on the compounds. Additionally, the solvatochromic and thermal effects of compound 6j are analysed. Among the tested BFPYOLs, the highest photoluminescent quantum yield (PLQY) was exhibited by 7k, achieving 91% in DMSO solvent. This study demonstrates that our synthetic methodology and the synthesised BFPYOLs can provide a powerful gateway to the generation of novel economic fluorescent probes.

## Abstract 2

This paper presents a metal-free synthetic protocol for assembling novel benzofuro[2,3-*c*]pyridin-3-ols (BFPYOLs) using 2,3-disubstituted benzofuran derivatives and their photoluminescence properties.

## Graphical abstract

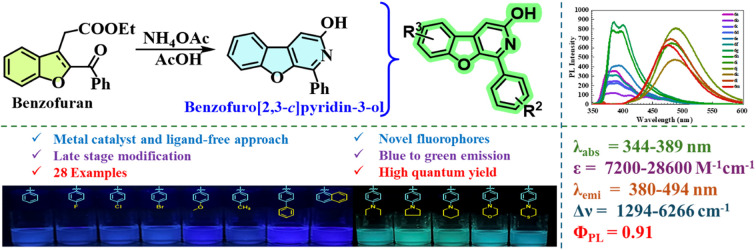

## Introduction

**Fig. 1:**
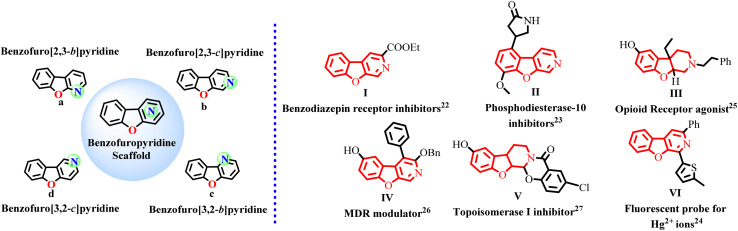
Types of benzofuropyridine core and examples of biologically active benzofuro[2,3-*c*]pyridine-based compounds.

Fluorescent organic molecules with flexible chemical structures and tunable photophysical properties continue to attract substantial interest for their applications in organic light-emitting devices (OLEDs),^1,2^ modern material chemistry, biomedical research for micro-macroscopic applications,^3–7^ for pathogen detection,^8–10^ disease diagnosis^11,12^ and quantitative analysis.^13^ High photoluminescent quantum yield (PLQY) organic fluorophores in solution under ambient settings enable economic, high-throughput manufacturing of devices on various substrates.^14^ Developing highly emissive organic materials from simple starting materials, with the potential for later-stage modification of their chemical structures, is a significant research goal. Benzofuropyridine derivatives are a significant class of luminescent tricyclic N-heterocycles, consisting of combinations of annulated pyridine, furan, and benzene ring structures. These compounds are further categorised into four groups based upon the position of the *N*-atom in the pyridine ring: (a) benzofuro[2,3-*b*]pyridine, (b) benzofuro[2,3-*c*]pyridine, (c) benzofuro[3,2-*b*]pyridine, (d) benzofuro[3,2-*c*]pyridine. These cores are evident in natural products,^15^ medicinal^16–21^ and luminescent materials. Despite its significant potential in applications, benzofuro[2,3-*c*]pyridine has been less explored than other benzofuropyridine structures, yet it exhibits diverse potent biological activities and holds interesting properties for material science. For instance, ethyl benzofuro[2,3-*c*]pyridine-based analogues have been discovered to be a non-benzodiazepine anticonvulsant (I),^22^ phosphodiesterase-10 inhibitor (II)^23^ and ratiometric fluorescent probe (VI)^24^ for Hg^2+^ metal ions. Other examples are of benzofuro[2,3-*c*]pyridin-6-ols based analogues, which are reported as opioid receptor agonists (III),^25^ MDR modulator (IV)^26^ and topoisomerase inhibitors (V)^27^ (Fig. 1).

**Scheme 1:**
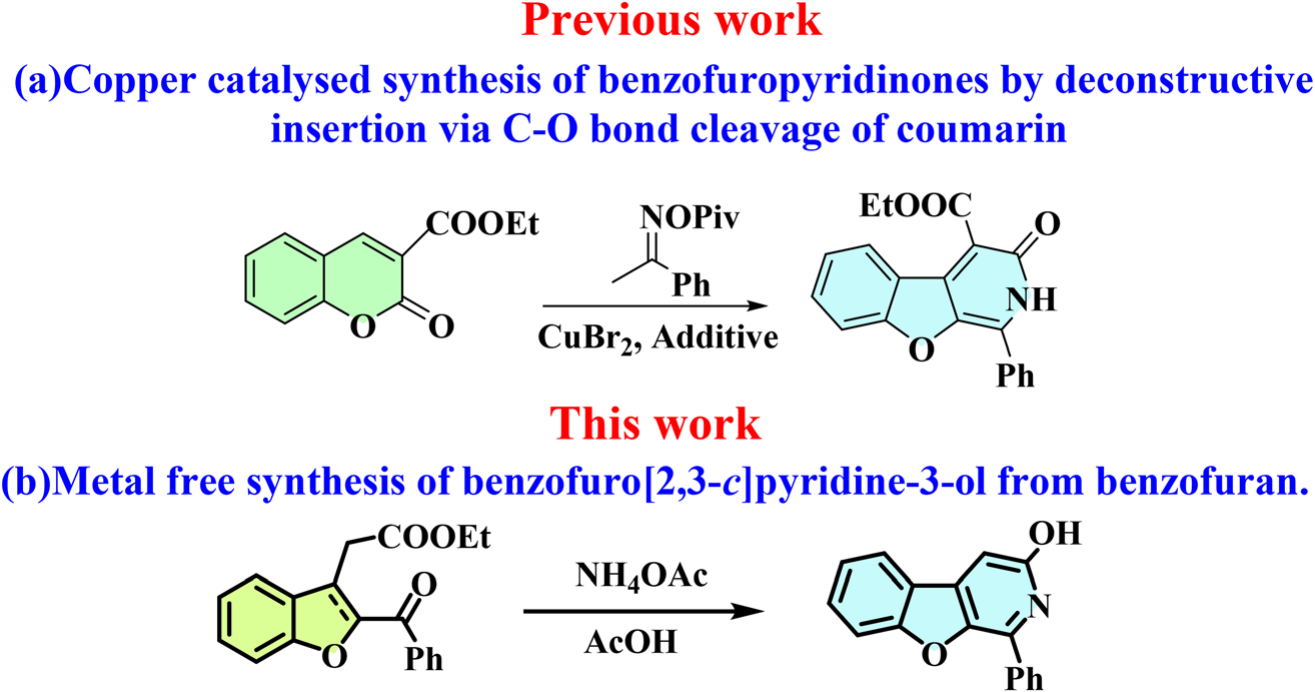
(a) Previous work: metal-catalysed synthesis of benzofuran-fused pyridinones from coumarin,^34^ (b) present work: metal-free synthesis of benzofuran-fused pyridinol from 2,3-disubstituted benzofuran derivatives.

Multiple reports discuss various substituted benzofuro[2,3-*c*]pyridine and related analogues, highlighting their synthetic methods and significance. Fayol A. *et al.* developed a multicomponent method for the synthesis of tetrahydrofuro[2,3-*c*]pyridines by heating a mixture of an aminopentynoate, aldehyde, and isocyanoacetamide in the presence of ammonium chloride.^28^ Hu J. *et al.* developed a protocol for synthesising polysubstituted benzofuro[2,3-*c*]pyridines using bromoacetophenone, a functionalized α,β-unsaturated ketone, and ammonium acetate.^29^ Xiong W. *et al.* developed a method for the synthesis of benzofuro[2,3-*c*]pyridine *via* Pd(ii)-catalysed cascade reactions of 2-(cyanomethoxy)chalcones with aryl boronic acids. The reaction cascade involves the formation of C–C/C–C/C–N bonds through nitrile carbopalladation, intramolecular Michael addition, cyclisation, and aromatisation.^30^ Xiong W. *et al.* prepared a diverse range of 3-aryl-1-(thiophen-2-yl)benzofuro[2,3-*c*]pyridines *via* a Pd-catalysed tandem reaction of 2-(cyanomethoxy)chalcones with thiophenes through direct C–H addition and sequential intramolecular conjugate addition and aromatisation using organic ligands. From the reported series, a compound was found to be a ratiometric fluorescent probe for Hg^2+^ ions.^24^ Clarkson G. J. *et al.* assembled fused benzofuran heterocycles *via ortho*-lithiation using LDA and zincation using zinc chloride and palladium catalysed Negishi cross-coupling of 2-bromophenyl acetates and fluoropyridines/fluoroarenes in the presence of base potassium *tert*-butoxide. The acyl group is deprotected *in situ*, followed by intramolecular aromatic nucleophilic substitution, forming benzofuropyridines and dibenzofurans.^31^ Hutchison A. J. *et al.* synthesised a series of novel analogues of anti-nociceptive *cis*-1,2,3,4,4a,9a-hexahydrobenzofuropyridin-6-ols as opioid-receptor subtypes modulators using hydroxy propiophenone, ethyl bromo acetate, and diethyl(cyanomethyl) phosphonate *via* a multistep process.^32^ Wang X. *et al.* developed a method of construction of N-heterocyclic benzofuro[3,2-*b*]pyridin-2-ones through β-activation of alkynoic acid esters using NHC-carbene catalyst following [3 + 3] annulation of alkynyl acylazoliums with benzofuran-3-amines.^33^ Zheng T. Y. *et al*. disclosed a very close study describing a copper-catalysed radical-mediated annulation reaction *via* ring opening of the lactone ring of coumarins and insertion of oximes to prepare dihydro-benzofuran-fused pyridinones^34^ (Scheme 1).

The processes mentioned above to prepare tricyclic benzofuro[2,3-*c*]pyridine-based compounds are complex because of the expensive starting materials, multistep reaction and purification stages, harsh reaction conditions, and organometallic catalysts. The direct and rapid synthesis protocol for diversified organic fluorescent molecules from easily accessible starting materials has great interest to synthetic chemists and industrial organisations actively working on fluorescent material projects. In pursuit of innovative methodologies for the synthesis of the benzofuro[2,3-*c*]pyridine core, we present a metal-free, efficient, and practical approach to prepare BFPYOL compounds. The synthesised organic molecules exhibit strong photoluminescence properties, ranging from blue to green, and possess a high PLQY. The benzofuro[2,3-*c*]pyridine core is valuable in advancing the development of cutting-edge chemical and biosensors. Moreover, this approach can also be applied to the synthesis of benzo[4,5]thieno[2,3-*c*]pyridine and 9*H*-pyrido[3,4-*b*]indole analogues by using respective thiophene and indole-containing heteroarenes as reaction starting materials.

### Retrosynthetic approach for the target scaffold

**Fig. 2:**
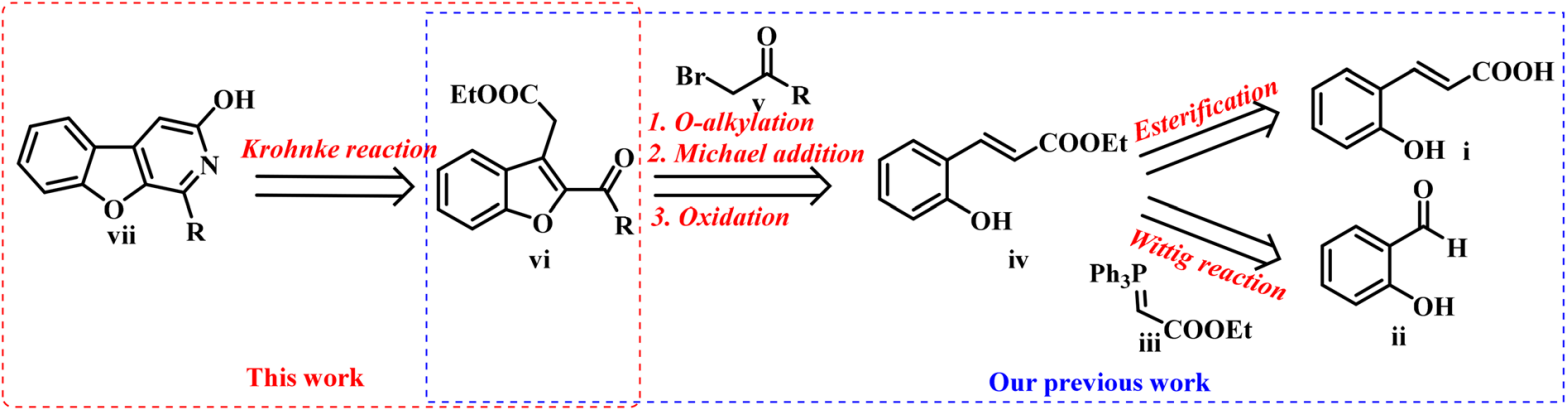
Retrosynthetic analysis of the target scaffold BFPYOL (vii).

Based on retrosynthesis analysis, BFPYOLs can be synthesised sequentially using the Wittig reaction, O-alkylation, Michael addition, oxidation, and the Krohnke reaction (Fig. 2).

## Results and discussion

**Scheme 2:**
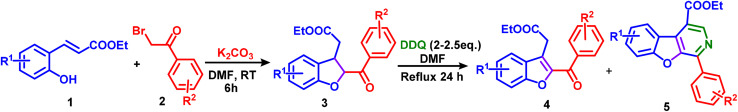
Our related previous work for the synthesis of benzofuran and benzofuro(2,3-*c*) pyridine-based compound.^36^

Based on the organic and medicinal significance of benzofuran derivatives, we have previously developed a domino methodology for the synthesis of 2,3-disubstituted benzofurans (3/4) and 1-phenylbenzofuro[2,3-*c*]pyridine-based molecules (5) from chalcones^35^ and 2-hydroxyethyl cinnamate (1)^36^ (Scheme 2).

Owing to the chemical reactivity of synthesised multi-functionalised benzofuran derivatives and continuation of our work in the field, we envisioned the synthesis of various 1-phenylbenzofuro[2,3-*c*]pyridin-3-ol analogues (BFPYOLs) from 2,3-disubstituted benzofuran (4) derivatives with a source of nitrogen. We commenced the work by synthesising starting materials, such as benzofurans, using our previously reported method (general procedures A, B, C, SI). For the synthesis of BFPYOLs, a trial reaction of ethyl 2-(2-benzoylbenzofuran-3-yl)acetate (4a) and 1 eq. of ammonium hydroxide aqueous solution with acetonitrile solvent was refluxed for six hours, but no product formation was observed. After continuing the reaction for 12 hours, delightfully, a trace amount of product 1-phenyl benzofuro[2,3-*c*]pyridin-3-ol (6a) formation was observed, identified by a blue-fluorescent appearance on TLC on 365 nm UV-irradiation and HRMS analysis of the crude reaction mixture.

**Scheme 3:**
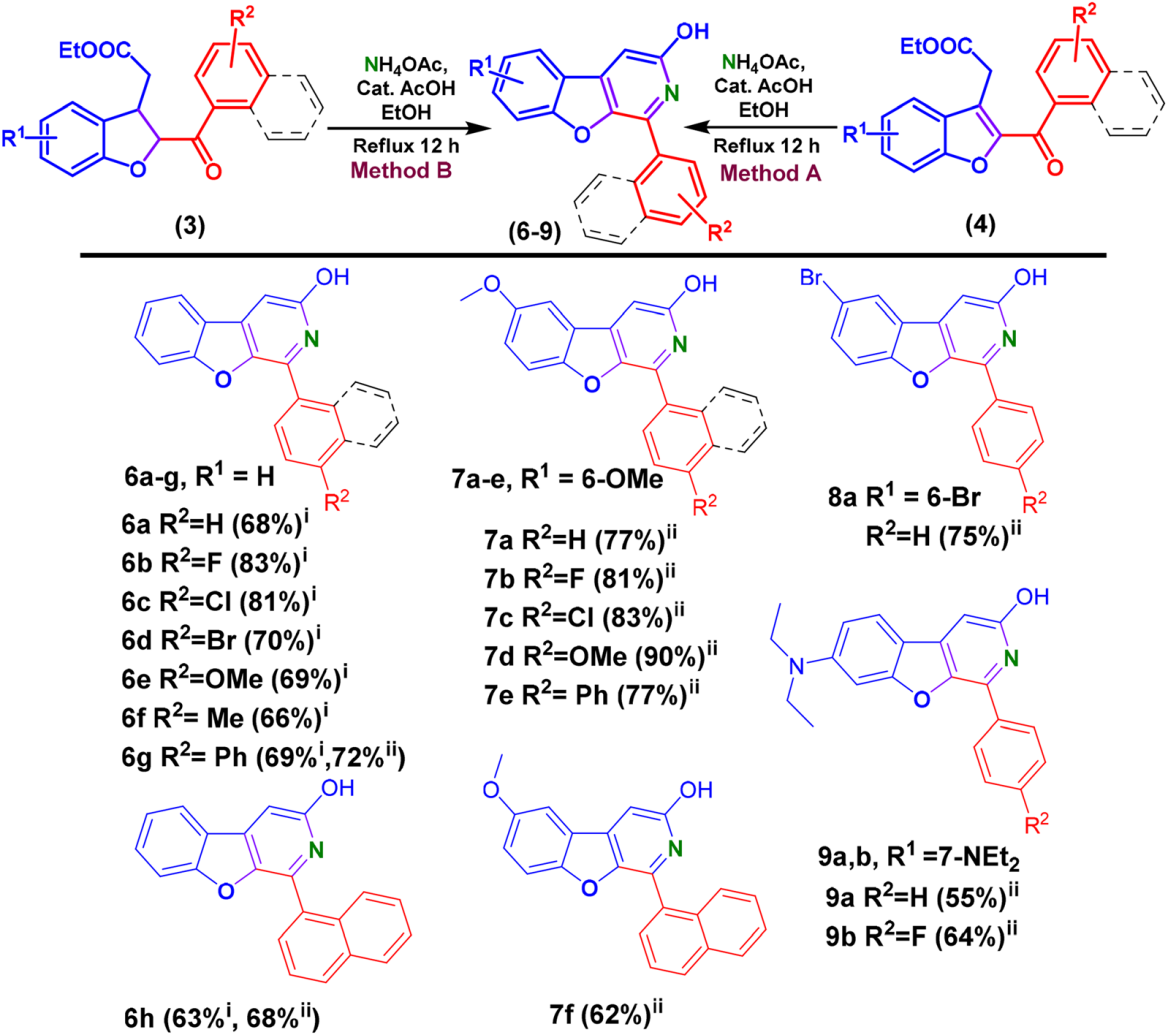
Synthesis of photoluminescent 1-phenylbenzofuro[2,3-*c*]pyridin-3-ol analogues (BFPYOLs) from 2,3-dihydrobenzofuran and benzofuran compounds. ^*i*^Isolated yield with method A, ^*ii*^isolated yield with method B.

Encouraged by the positive results, and to improve the yield of 6a, a stoichiometric amount of different ammonia reagents such as aqueous ammonium solution (entries 1–3, 7–9), methanolic ammonia (entry-4), ammonium acetate (entries 5, 6, 10–15) in different solvents such as acetone, methanol, ethanol, water : ethanol, water : acetonitrile with (entries 7–15) and without (entries 1–6) the presence of a cat. acetic acid was screened (Table 1). Among the tested conditions, 4a dissolved in ethanol, with ammonium acetate (10 eq.) and a catalytic amount of acetic acid (0.1 eq.), led to the cleanest and most efficient reaction with 68% formation of 6a (general procedure E, SI). On addition of ammonium acetate, pyridin-3-ol (ring C) is spawned by the formation of two C–N bonds, between the 1,5-dicarbonyl fragment of ethyl 2-(2-benzoylbenzofuran-3-yl)acetate 4a and the ammonium *N*-atom. After establishing the optimal reaction conditions, the method's applicability for constructing differently substituted fused BFPYOLs was evaluated (Scheme 3). The benzofurans (4) derived from substituted 2-hydroxyethylcinnamates (1) and 2-bromoacetophenones (2) smoothly reacted with ammonium acetate to furnish the corresponding BFPYOLs in moderate to good yields with tolerance of various functional groups such as halogens (F, Cl, Br), methyl, phenyl, naphthyl, and methoxy.

**Table 1:** Optimisation of reaction conditions for the synthesis of 1-phenyl benzofuro[2,3-*c*]pyridin-3-ol (6a) from ethyl 2-(2-benzoylbenzofuran-3-yl)acetate (4a)^a^

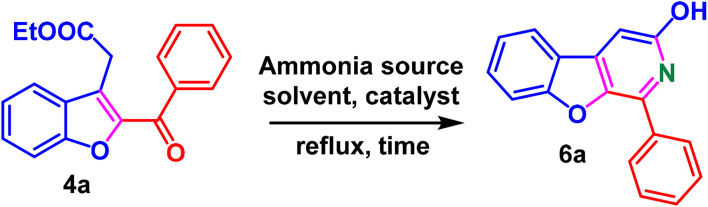					
Entry	Ammonia source (eq.)	Acid catalyst^b^ (0.1 eq.)	Solvent	Time (h)	6a-yield^c^ (%)
1	Aq. NH_4_OH (1)	—	CH_3_CN	6	NP
2	Aq. NH_4_OH (1)	—	CH_3_CN	12	Trace
3	Aq. NH_4_OH (excess)	—	Acetone	12	NP
4	7 M methanolic ammonia (excess)	—	—	12	15
5	NH_4_OAc (excess)	—	EtOH	12	NP
6	NH_4_OAc (5)	—	EtOH : H_2_O (1 : 1)	12	8
7	Aq. NH_4_OH (excess)	AcOH	CH_3_CN	12	10
8	Aq. NH_4_OH (excess)	AcOH	CH_3_OH	12	12
9	Aq. NH_4_OH (excess)	AcOH	EtOH	12	22
10	NH_4_OAc (5)	AcOH	EtOH	12	44
**11**	**NH**_**4**_ **OAc (10)**	**AcOH**	**EtOH**	**12**	**68**
12	NH_4_OAc (10)	AcOH	EtOH : H_2_O (1 : 1)	12	50
13	NH_4_OAc (10)	AcOH	CH_3_CN : H_2_O (1 : 1)	12	48
14	NH_4_OAc (10)	AcOH	CH_3_CN	12	28
15	NH_4_OAc (15)	AcOH	EtOH	12	62

a General reaction conditions: ethyl 2-(2-benzoylbenzofuran-3-yl)acetate (4a) (0.3 mmol), ammonia source in solvents (4–5 ml) at reflux for the mentioned time.

b Catalytic amount (0.1 eq.).

c Isolated yield of 6a.

**Scheme 4:**
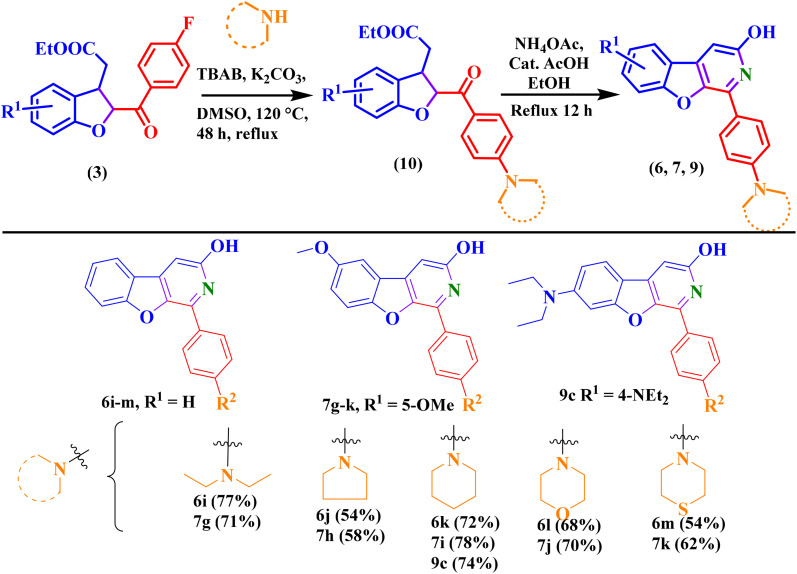
Synthesis of highly emissive *para-N* substituted BFPYOLs.

Additionally, the established reaction condition was also found suitable for a series of ethyl-2-(2-benzoyl-2,3-dihydrobenzofuran-3-yl)acetates to prepare corresponding BFPYOLs in good to moderate yield. This finding offered the benefit of simplifying the preparation of BFPYOLs by reducing the number of steps involved. The 6g–h, 7a–f, 8a, 9a–b were prepared using the direct approach from respective dihydro-benzofurans (3) (general procedure F, SI). Impressed with the substituent's effect on the emission colour of the BFPYOLs, some derivatives bearing *para-N*-saturated heterocycles, including piperidine, pyrrolidine, morpholine, and thiomorpholine, were designed and synthesised using Scheme 4. Purposefully, first the ethyl 2-(2-(4-fluorobenzoyl)-2,3-dihydrobenzofuran-3-yl)acetate (3b) was prepared (general procedure B. SI) and then the substitution of *para*-fluorine atom with secondary amines such as *N*,*N*-diethylamine, piperidine, pyrrolidine, morpholine, and thiomorpholine using TBAB (0.1 eq.), K_2_CO_3_ (1 eq.), DMSO and refluxed at 120 °C for 48 h (general procedure D, SI). After preparation of ethyl 2-(2-(4-substituted amine-benzoyl)-2,3-dihydrobenzofuran-3-yl)acetates, the preparation of corresponding benzofuro[2,3-*c*]pyridin-3-ols was achieved using the direct approach discussed above (general procedure F, SI). As anticipated, fluorescence of these newly synthesised *para-N* substituted BFPYOLs displayed a significant difference in intensity and colour of luminescence on TLC on exposure to 365 nm UV light while reaction monitoring. With curiosity, photophysical studies were conducted to explore the photoluminescent properties of the synthesised molecules. Photophysical studies indicate that the derived BFPYOLs have significant potential for application in material chemistry and the development of diagnostic devices and sensors.

**Fig. 3:**
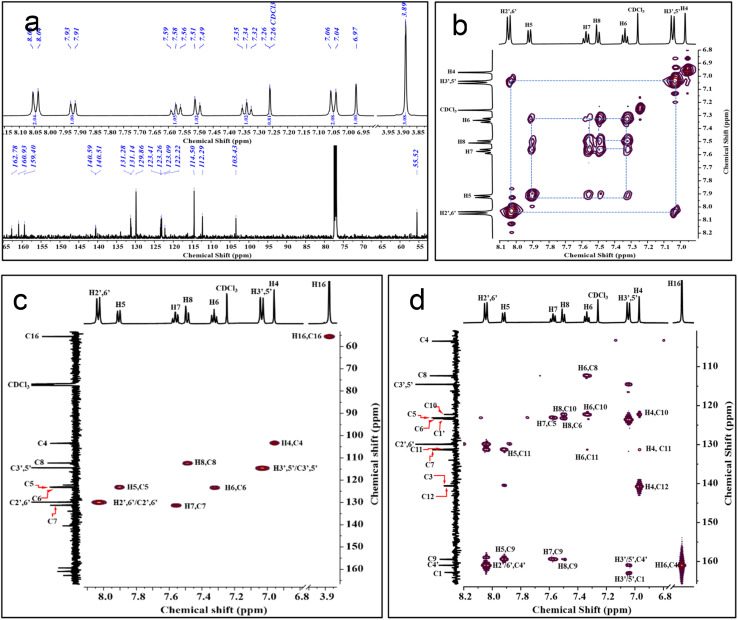
Structure confirmation and NMR signal assignments of 6e. (a) ^1^H NMR and ^13^C NMR spectra of 6e represent all the proton and carbon signals. Partial TOCSY (b) and HSQC (c) spectrum of 6e representing the ^1^H–^1^H correlation and ^1^H–^13^C correlation. Partial HMBC spectrum (d) of 6e representing the scalar and long-range ^1^H–^13^C correlation of protons and carbons.

**Fig. 4:**
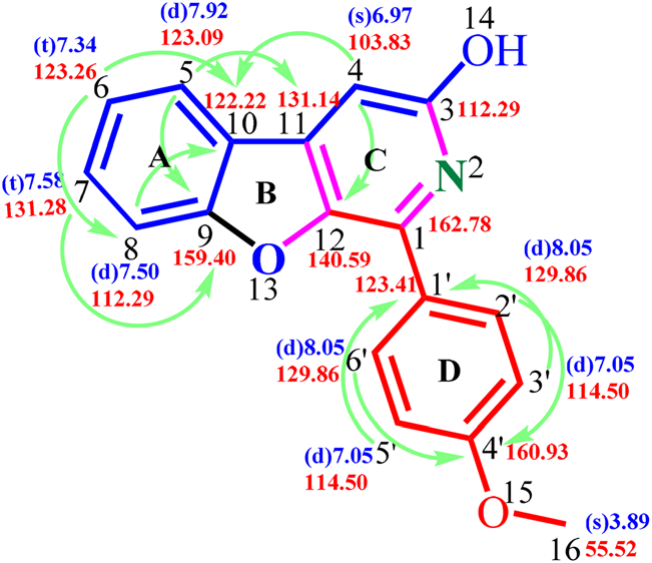
Structure of 6e with respective ^1^H NMR and ^13^C NMR signal assignments with a ^1^H–^13^C correlation of protons and carbons in 2D NMR.

There have been no documented cases of benzofuran-fused pyridin-3-ols, highlighting a significant gap in current research. To confirm the structure of this novel skeleton and the assignment of signals observed in 1D NMR spectra to their respective protons and carbon atoms in the framework, we decided to analyse the 2D NMR spectrum of the 6e (Fig. 3 and 4). The presence of *para*-substitution on the test molecule is advantageous in picking the obvious signal to initiate the signal assignment. ^1^H NMR and ^13^C NMR spectra confirm the presence of 12 protons and 18 carbons (Fig. 3(a)). The first signal to be recognised as rational for other signals assignment is the singlet at *δ* H 3.89 ppm corresponding to the protons of the *p*-methoxy group, and the respective carbon signal at *δ* C 55.5 ppm. The other singlet at *δ* H 6.97 ppm was anticipated and later assigned as H4, which exhibited no connectivity with other protons of ring A and D in the structure of 6e in the TOCSY spectrum (Fig. 3(b)). The corresponding carbon C4 at *δ* C 103.4 ppm was also confirmed by HSQC analysis. Other signals assigned to *o*-H3′, 5′ *δ* H 7.04–7.05, C2′, 6′ *δ* C 114.5 ppm and *m*-H2′, 6′ *δ* H 8.04–8.06 ppm, C2′, 6′ *δ* C 129.8 ppm to the methoxy group of ring D, Four CHs– of ring A at H5–C5 at *δ* H d-7.92 ppm *δ* C 123.1 ppm, H6–C6 at *δ* H t-7.34 ppm *δ* C 123.3 ppm, H7–C7 at *δ* H t-7.58 ppm *δ* C 131.3 ppm, H8–C8 at *δ* H d-7.50 ppm *δ* C 112.3 ppm, unambiguously with the TOCSY and HSQC connectivity (Fig. 3(c)). The assignments of all quaternary carbons *via* observed HMBC connectivity confirmed the signals C1 and C3 at *δ* C 162.8 ppm, C9 at *δ* C 159.4 ppm, C10 at *δ* C 122.2 ppm, C11 at *δ* C 131.1 ppm, C12 at *δ* C 140.6 ppm, C1′ at *δ* C 123.4 ppm, and C4′ at *δ* C 160.9 ppm (Fig. 3(d)). Two long-range couplings between H3′, 5′-C1 and H5–C3 are observed in HMBC analysis.

### Mechanism

**Scheme 5:**
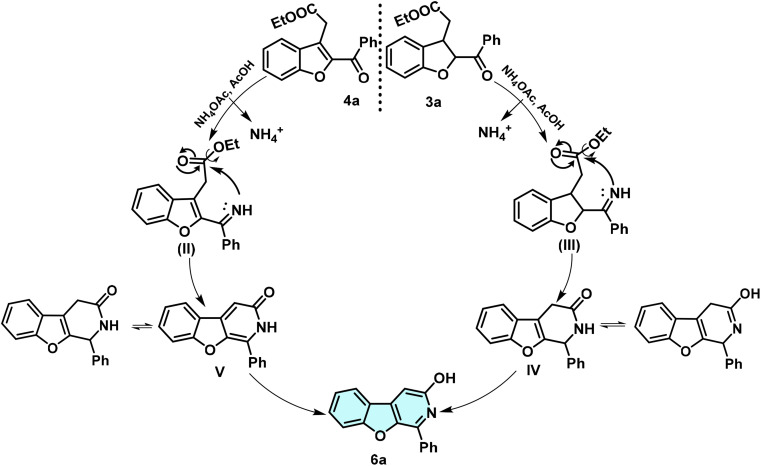
The chemical reaction mechanism for synthesising tricyclic ring system-benzofuro[2,3-*c*]pyridin-3-ol (6a) based compounds from 2,3-disubstituted benzofuran derivatives 3a/4a.

A possible reaction mechanism that accounts for the formation of the benzofuro[2,3-*c*]pyridin-3-ol 6a is depicted in Scheme 5. An intramolecular cascade condensation reaction of diketone 3a/4a and ammonia is the major chemistry in transforming the bicyclic to the tricyclic ring system. The carbonyl of the benzoyl group of benzofuran 4a reacts with the nitrogen atom of the released ammonia from ammonium acetate in the presence of acetic acid to form intermediate imine II, and further cyclisation of II gives a pyridin-3-ol ring to furnish benzofuro[2,3-*c*]pyridin-3-ol (6a) in the case of starting material 4a. Whereas, the dihydro-benzofuran compound 3a undergoes an intramolecular cascade condensation and annulation towards pyridin-3-ol ring to afford benzofuro[2,3-*c*]pyridin-3-ol 6a*via* intermediate III and sequentially 3,4-dihydrobenzofuro[2,3-*c*]pyridin-3-ol as intermediate IV (characterisation details, SI), which could be readily oxidised to a more stable π-conjugated planar tri-ring framework benzofuro[2,3-*c*]pyridin-3-ol.

### Photophysical studies

**Fig. 5:**
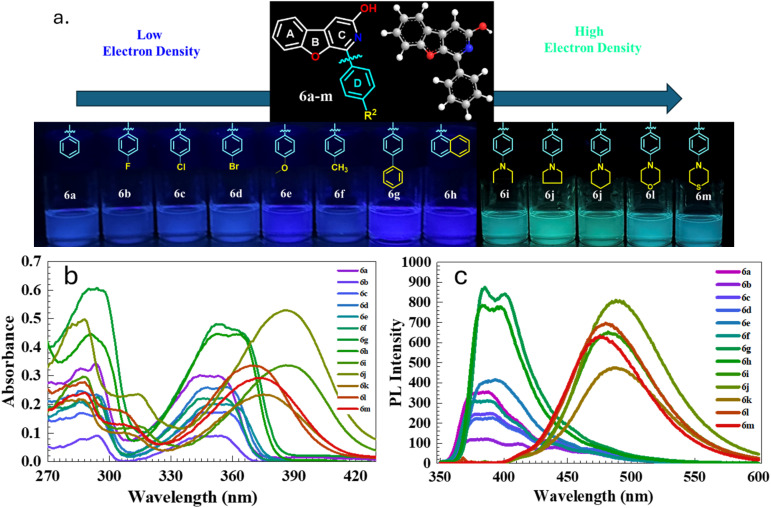
(a) Photograph of the compounds 6a–m in DMSO under 365 nm UV irradiation (b) UV-visible absorbance (20 μM) and (c) emission spectra (10 μM) of derivatives of 6, at 20 °C; excitation and emission slit width of 5, 5 nm, 600 V except 6g and 6h-570 V.

**Fig. 6:**
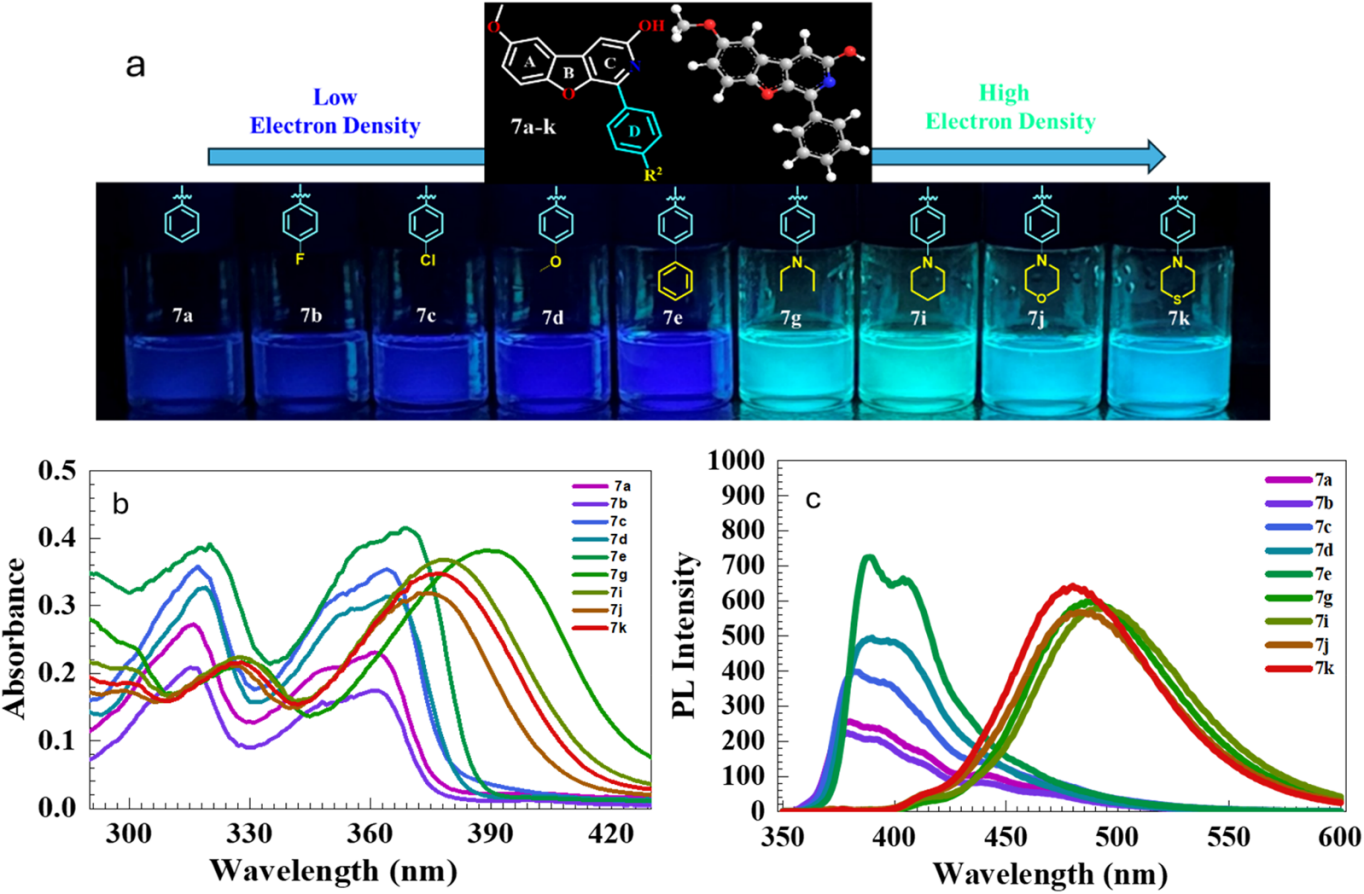
(a) Photograph of the compounds 7a–k in DMSO under 365 nm UV irradiation, (b) UV-visible absorbance (20 μM) and (c) emission spectra of derivatives (7a–k), 10 μM at 20 °C; excitation and emission slit width of 5; 5 nm, 600 V.

**Fig. 7:**
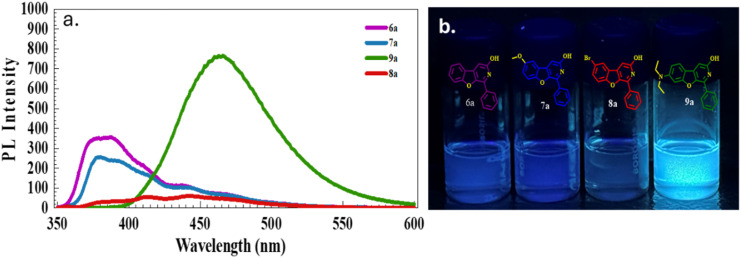
(a) Emission spectra of 6a, 7a, 8a, 9a, 10 μM at 20 °C; excitation and emission slit width of 5, 5 nm, 600 V representing the effect of different substitutions on ring A. (b) Photograph of the compounds in DMSO under 365 nm UV irradiation.

In this work, the synthesised benzofuro[2,3-*c*]pyridin-3-ol skeleton was a good candidate with strong photoluminescence in the solution state. The planar structure and the conjugated system of the core can be responsible for the photoluminescence of the compounds. To investigate the photophysical potential of the synthesised BFPYOLs, all pure compounds 6a–m, 7a–k, 8a, 9a–c were analysed for their photoluminescence properties. To simplify this study, we initially focused on the relationship between the photophysical properties and the *R*^2^ substituents at the *para*-position on ring D, rather than at other available positions on the core skeleton. The presence of various electron-withdrawing groups (EWGs) and electron-donating groups (EDGs) on ring A, combined with the *para*-substituted phenyl ring D and the free hydroxyl (–OH) group on ring C, significantly affects both the emission wavelength and PL intensity of the compounds. A comprehensive photophysical analysis of the molecules was conducted using DMSO as the solvent at 20 °C to elucidate their photophysical behaviour. The UV absorbance (*λ*_abs_) was measured at room temperature with a concentration of 20 μM for all derivatives. The maximum absorbance wavelength measured falls within the 344 to 389 nm range. The least *λ*_abs_ is exhibited by 6a-344 nm, containing no substitution on ring A and ring D. In contrast, the largest *λ*_abs_ is displayed by 7g-389 nm, which has 6-methoxy substitution at ring A and 4-*N*,*N*-diethyl substitution at ring D. The emission wavelength (*λ*_em_) of the test compounds ranges from 380 to 494 nm, displaying varying photoluminescence intensities. The measured *λ*_em_ of the test compounds exhibited a significant red shift of 114 nm, demonstrating that the substitution difference considerably affects the photophysical properties of the compounds. The maximum *λ*_em_ is associated with compound 7i-494 nm which has the methoxy at 6-positions on ring A and *para*-piperidinyl group on ring D. In contrast, the least *λ*_em_ is associated with compound 7a-380 nm consisting of methoxy at 6-position on ring A and simple phenyl with no substitution as ring D. The emission effect of the different EWGs and EDGs present on ring D of the test compounds; without 6-methoxy group (6a–m, Fig. 5 and Table 2) and with 6-methoxy group (7a–k, Fig. 6 and Table 3) on ring A is studied and summarised. The effect of different EWG and EDG on ring A on emission wavelength and intensity is also compared and summarised for the compounds 6a, 7a, 8a, 9a (Fig. 7 and Table 4), which has a simple unsubstituted ring D. All emission spectra display broad emission bands, which are typical for the intramolecular charge transfer (ICT) transition. The Stokes shift was measured to be maximum for compound 6l with a value of 6266 cm^−1^ and minimum for compound 7c with 1294 cm^−1^. The synthesised BFPYOLs containing alkylated amines such as *N*,*N*-diethyl (both ring A and D), pyrrolidine, piperidine, morpholine, and thiomorpholine in the molecular frame have displayed a significant red shift in the series. The increased electron density at position *R*^2^ leads to a bathochromic shift in the emission wavelength. Changes in substituents from hydrogen (6a) to *p*-methyl (6f), *p*-methoxy (6e), *p-N*,*N*-diethylamino (6i), and *p*-piperidyl (6k) resulted in red shifts in the emission wavelengths, measuring 387 nm, 388 nm, 393 nm, 482 nm, and 488 nm, respectively. Changes to the substituents at *R*^1^ lead to various shifts in emission wavelengths. Specifically, replacing hydrogen (6a) with bromine (8a) at the 6-position and with *N*,*N*-diethylamine (9a) at the 7-position results in a red shift in emission wavelengths to 387 nm, 442 nm, and 466 nm, respectively. Conversely, substituting hydrogen (6a) with methoxy (7a) at the 6-position causes a slight blue shift in the emission wavelength, changing it from 387 nm to 380 nm (Table 5).

**Table 2:** Photophysical properties of the BFPYOLs containing compounds (6a–m)

Comp. code	*R* ^1^	*R* ^2^	*λ*_abs_ a (nm)	*λ*_em_ b (nm)	PL intensity	Stokes shift (cm^−1^)	PLQY^c^ (*Φ*_PL_)	Molar absorption coefficient (*ε* (M^−1^ cm^−1^))
6a	H	H	344	387	355	3229	0.41	14 100
6b	H	4-F	354	385	121	2274	nd	nd
6c	H	4-Cl	356	389	250	2382	0.48	7200
6d	H	4-Br	356	391	228	2514	0.30	13 300
6e	H	4-OCH_3_	353	393	416	2883	0.51	11 800
6f	H	4-CH_3_	355	388	314	2395	0.38	11 500
6g	H	4-Ph	352	385	875	2435	0.59	23 800
6h	H	Naph	353	383	798	2218	0.52	24 300
6i	H	4-NEt_2_	386	482	653	5159	0.88	16 800
6j	H	4-Pyrrolidine	386	488	813	5414	0.80	26 300
6k	H	4-Piperidine	375	488	476	6174	0.83	12 500
6l	H	4-Morpholine	369	480	695	6266	0.88	18 400
6m	H	4-Thiomorpholine	373	475	630	5757	0.88	14 700

a Absorbance with 20 μM.

b Emission with 10 μM recorded for benzofuro[2,3-*c*]pyridinol-based compounds (6a–m) in solvent DMSO at 20 °C; excitation and emission slit width of 5; 5 nm, 600 V except 6g and 6h (570 V) for emission.

c Relative PL quantum yield (*Φ*_PL_) is calculated in DMSO solvent with reference quinine sulphate (reported *Φ*_PL_ = 0.54, calculated = 0.534 ± 0.04 at 360 nm in 0.1 M H_2_SO_4_).

**Table 3:** Photophysical properties of the benzofuro[2,3-*c*]pyridinol-based compounds (7a–k)

Comp. code	*R* ^1^	*R* ^2^	*λ*_abs_ a (nm)	*λ*_em_ b (nm)	PL intensity	Stokes shift (cm^−1^)	PLQY^c^ (*Φ*_PL_)	Molar absorption coefficient (*ε* (M^−1^ cm^−1^))
7a	6-OCH_3_	H	361	380	260	1385	0.36	10 100
7b	6-OCH_3_	4-F	361	379	224	1315	0.37	9500
7c	6-OCH_3_	4-Cl	364	382	401	1294	0.42	15 100
7d	6-OCH_3_	4-OCH_3_	366	390	495	1681	0.50	14 400
7e	6-OCH_3_	4-Ph	369	389	735	1393	0.56	19 800
7g	6-OCH_3_	4-NEt_2_	389	486	598	5130	0.84	16 200
7i	6-OCH_3_	4-Piperidine	378	494	580	6212	0.81	16 400
7j	6-OCH_3_	4-Morpholine	372	483	568	6177	0.89	15 700
7k	6-OCH_3_	4-Thiomorpholine	377	480	639	5691	0.91	16 900

a Absorbance with 20 μM.

b Emission with 10 μM recorded for benzofuro[2,3-*c*]pyridinol-based compounds (7a–k) in solvent DMSO at 20 °C; excitation and emission slit width of 5; 5 nm, 600 V.

c Relative PL quantum yield (*Φ*_PL_) is calculated in DMSO solvent with reference quinine sulphate (reported *Φ*_PL_ of 0.54, calculated 0.534 ± 0.04 at 360 nm in 0.1 M H_2_SO_4_).

**Table 4:** Photophysical properties of the benzofuro[2,3-*c*]pyridinol based compounds (6a, 7a, 8a, 9a)

Comp. code	*R* ^1^	*R* ^2^	*λ*_abs_ a (nm)	*λ*_em_ b (nm)	PL intensity	Stokes shift (cm^−1^)	PLQY^c^ (*Φ*_PL_)	Molar absorption coefficient (*ε* (M^−1^ cm^−1^))
6a	H	H	344	387	355	3229	0.41	14 100
7a	6-OCH_3_	H	361	380	260	1385	0.36	10 100
8a	6-Br	H	357	442	60	5386	0.09	9900
9a	7-NEt_2_	H	368	466	767	5714	0.69	28 600

a Absorbance with 20 μM.

b Emission with 10 μM recorded for benzofuro[2,3-*c*]pyridinol-based compounds (6a, 7a, 8a, 9a) in solvent DMSO at 20 °C; excitation and emission slit width of 5; 5 nm, 600 V.

c Relative PL quantum yield (*Φ*_PL_) is calculated in DMSO solvent with reference quinine sulphate (reported *Φ*_PL_ of 0.54, calculated 0.534 ± 0.04 at 360 nm in 0.1 M H_2_SO_4_).

**Table 5:** Photophysical properties of the benzofuro[2,3-*c*]pyridinol-based compounds (9a–c)

Comp. code	*R* ^1^	*R* ^2^	*λ*_abs_ a (nm)	*λ*_em_ b (nm)	PL intensity	Stokes shift (cm^−1^)	PLQY^c^ (*Φ*_PL_)	Molar absorption coefficient (*ε* (M^−1^ cm^−1^))
9a	7-NEt_2_	H	368	466	767	5714	0.69	28 600
9b	7-NEt_2_	4-F	368	460	984	5434	0.70	31 400
9c	7-NEt_2_	4-Piperidine	377	462	460	4880	0.85	11 200

a Absorbance with 20 μM.

b Emission with 10 μM recorded for benzofuro[2,3-*c*]pyridinol-based compounds (9a–c) in solvent DMSO at 20 °C; excitation and emission slit width of 5; 5 nm, 600 V.

c Relative PL quantum yield (*Φ*_PL_) is calculated in DMSO solvent with reference quinine sulphate (reported *Φ*_PL_ of 0.54, calculated 0.534 ± 0.04 at 360 nm in 0.1 M H_2_SO_4_).

We developed a series of novel photoluminescent compounds by modifying the *R*^1^ and *R*^2^ positions on the BFPYOL skeleton, resulting in emission in the visible region. Furthermore, additional structural modifications can fine-tune the emission properties, opening the door for a new fluorophore emitting high intensity in the near-infrared region.

**Fig. 8:**
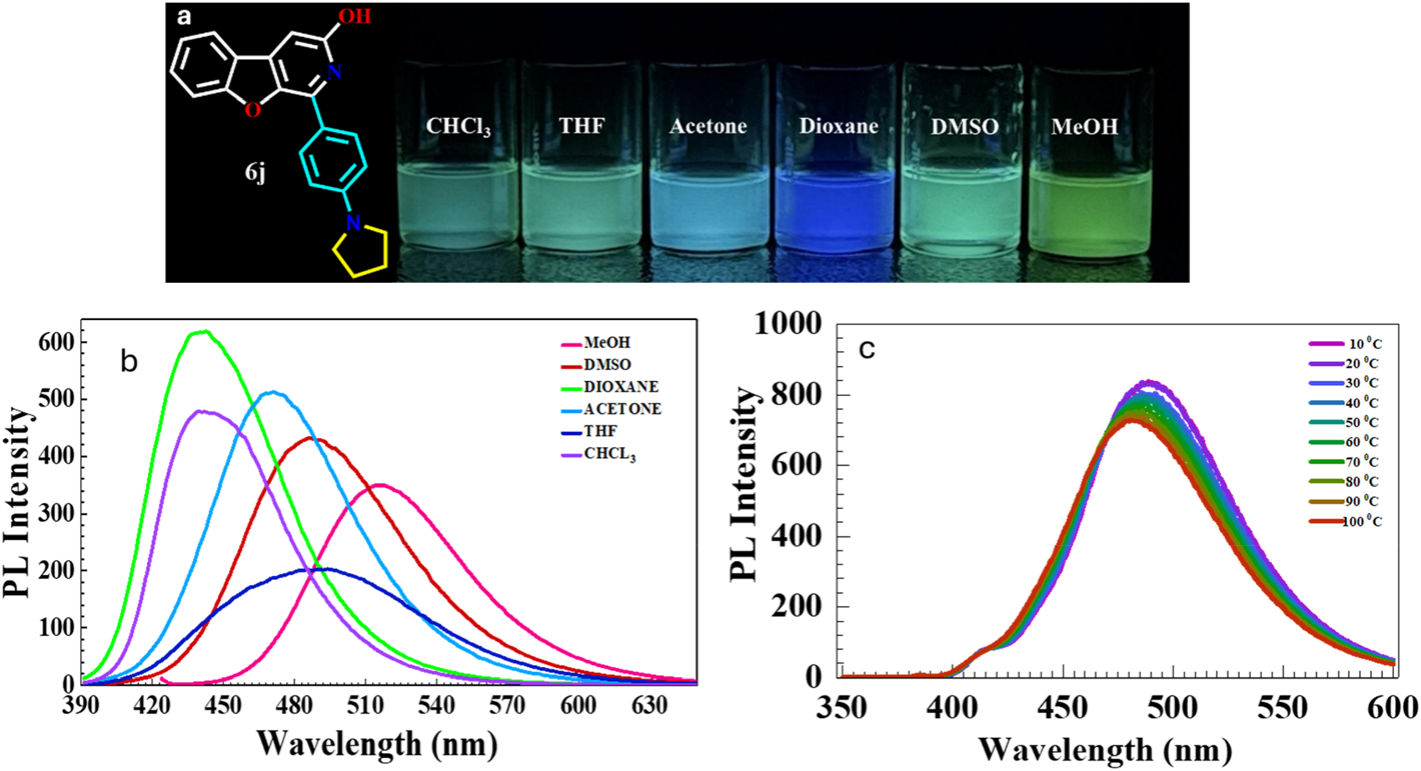
(a) Photograph of the compound 6j in different solvents under 365 nm UV irradiation (*c* = 5 μM), (b) emission spectra of 6j in different solvents excited at maximum absorption wavelength, displaying the effect of solvent polarity on emission wavelength and intensity; 5 μM at 20 °C; (c) emission spectra of 6j displaying the thermal effect on emission intensity 10 μM in DMSO solvent at 10–100 °C. Excitation and emission slit width of 5; 5 nm, 600 V.

The photophysical properties of fluorophores change with solvent polarity and temperature. The solvatochromic and thermal effects on *λ*_em_ and PL intensity for molecule 6j were evaluated. A 5 μM solution of 6j was prepared in various solvents (CHCl_3_, 1,4-dioxane, THF, acetone, DMSO, MeOH), and UV absorbance and emission spectra were recorded. All conditions were kept consistent, and results are summarised in Fig. 8(a, b) and Table 6. The effect of solvent polarity on the emission wavelength and intensity of the test compound showed a significant difference. As the solvent polarity increased, a bathochromic shift in emission was observed from 443 nm to 518 nm, while the PL intensity exhibited a hypochromic shift. Specifically, in the cases of CHCl_3_ and 1,4-dioxane, the compound displayed similar emission at 443 nm and in THF at 495 nm. In contrast, the emission in MeOH was observed at 518 nm, indicating both a bathochromic and hypochromic shift compared to DMSO, which emitted at 488 nm. The emission in 1,4-dioxane and CHCl_3_ remained at 443 nm, demonstrating a hypsochromic and hyperchromic shift. The optical analysis, conducted with increasing solvent polarity, highlighted the polar fluorophore characteristics of the BFPYOL derivatives. In addition, a slight variation in the *λ*_em_ was observed for the different solvent samples compared to the previous study when excited at the same absorption wavelength (*λ*_abs_ = 386 nm) (see Table 1, SI). Furthermore, the impact of temperature, ranging from 10 to 100 °C, on the emission wavelength of the test compound 6j was evaluated in DMSO solvent at a concentration of 10 μM. The measured change in photoluminescence PL intensity for each 10 °C increase was 0.15-fold, with values ranging from 838 to 728, as illustrated in Fig. 8(c).

**Table 6:** Absorption and emission data of 6j in different solvents

Sr. no.	Solvent	*λ*_abs_ a (nm)	*λ*_em_ b (nm)	PL intensity	Stokes shift (cm^−1^)	PLQY^c^ (*Φ*_PL_)
1	Chloroform (CHCl_3_)	385	443	480	3400	0.44
2	Tetrahydrofuran (THF)	384	495	204	5839	0.45
3	Acetone	373	471	512	5578	0.47
4	1,4-Dioxane	373	443	621	4236	0.83
5	Dimethyl sulfoxide (DMSO)	386	488	432	5414	0.79
6	Methanol (MeOH)	424	518	350	4279	0.43

a Absorbance.

b Emission recorded for 6j, excited at maximum absorption wavelength, 5 μM concentration in different solvents; at *T* = 25 °C; excitation and emission slit width of 5, 5 nm, 600 V.

c Relative PL quantum yield (*Φ*_PL_) is calculated in the mentioned solvent with reference quinine sulphate (reported *Φ*_PL_ of 0.54, calculated 0.534 ± 0.04 at 360 nm in 0.1 M H_2_SO_4_)

### Photoluminescence quantum yield (*Φ*_PL_)

Following the successful analysis of *λ*_abs_ and *λ*_em_ for the BFPYOLs, a systematic study was planned to determine the relative PLQY (*Φ*_PL_) in DMSO using quinine sulphate as a standard, since all compounds have an excitation wavelength range of 340–400 nm and an emission wavelength range of 375–495 nm. The relative quantum yield of the test samples was determined using the standard procedure^37^ (refer to SI).

**Fig. 9:**
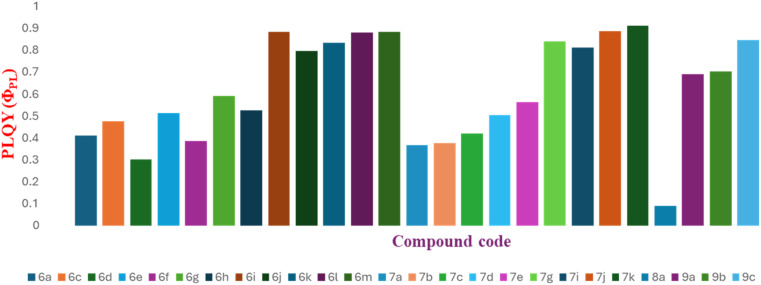
A graphical representation of quantum yield determined for the derivatives of BFPYOLs in DMSO solvent with standard quinine sulphate (reported *Φ*_PL_ of 0.54, calculated 0.534 ± 0.04 at 360 nm in 0.1 M H_2_SO_4_).

The findings are summarised by a graphical representation (Fig. 9 and Tables 2–5). The results suggest that BFPYOL compounds show intense absorption and broad fluorescence bands with the highest *Φ*_PL_ 0.91 (7k). Introduction of electron-rich *p-N*-substitutions, such as *p-N*,*N*-diethylamino (6i, 7g), *p*-pyrrolidine (6j), *p*-piperidine (6k, 7i, 9c), *p*-morpholine (6l, 7j), and *p*-thiomorpholine (6m, 7k) on ring D of the BFPYOL skeleton resulted in high *Φ*_PL_ ≥ 0.80. Similarly, substituent changes on ring A from H (6a) to *N*,*N*-diethylamine (9a) at 7-position exhibited considerable improvement in *Φ*_PL_ values, increasing from 0.41 to 0.69. The determined properties highlight the potential fluorophore nature of these compounds, which can be utilised as economic fluorescent probes in the development of new theragnostic tools. The target compounds' molar absorption coefficient (*ε*) is determined from the slope of the straight line (intercept zero, and *R*^2^ near 1) in the graph by plotting UV absorbance against the six concentrations (refer to SI).

## Conclusion

In summary, we have demonstrated a metal-free process for the synthesis of a novel series of high PLQY possessing tricyclic benzofuro[2,3-*c*]pyridin-3-ol (BFPYOL) compounds from 2,3-di-substituted benzofuran derivatives. A complete structure elucidation *via* NMR signal assignment for a novel benzofuro[2,3-*c*]pyridin-3-ol-based compound (6e) is done *via* 2D NMR analysis, and all the synthesised compounds are well characterised using ^1^H, ^13^C NMR, and HRMS. The photophysical parameters and PLQY of the synthesized BFPYOLs indicate their potential as excellent functional materials. The presence of various EWGs and EDGs at different positions on the BFPYOL skeleton significantly influences its photoluminescent properties. Compounds with high electron density on rings A and D of the BFPYOL structure (6i–m, 7g, 7i–k, 9a–c) exhibit considerable red shifts in emission wavelength and high PLQY values ranging from 0.80 to 0.91. Conversely, compounds with aromatic substituents on ring D (6g, 6h, 7e) display high photoluminescence intensity. These insights can help fine-tune the optical properties of BFPYOLs. We anticipate that this synthesis method for novel bicyclic and tricyclic compounds will have significant implications for future research in chemistry and related fields.

## Author contributions

S. M. S.: conceptualisation, methodology, synthesis, characterisation, photophysical study, investigation, data curation, formal analysis, writing – original draft and editing; S. C.: conceptualisation, methodology, characterisation, formal analysis, writing – review editing, supervision.

## Conflicts of interest

The authors declare there is no conflict of interest.

## Supplementary Material

RA-015-D5RA05420F-s001

## Data Availability

The authors declare that all the required spectral data are available in the SI. Supplementary information: the synthetic procedure, characterisation details, and spectral information. See DOI: https://doi.org/10.1039/d5ra05420f.
