# Supplementary material for: Metal-free synthesis of tricyclic benzofuro[2,3-c]pyridin-3-ol derivatives, characterization, and photoluminescence properties
Source: RSC Adv. 2025 Sep 4;15(38):31840–52. doi: 10.1039/d5ra05420f (PMC12409613; doi:10.1039/d5ra05420f)
Supplement: RA-015-D5RA05420F-s001 [file RA-015-D5RA05420F-s001.pdf]

Supplementary Information (SI) for RSC Advances.
This journal is © The Royal Society of Chemistry 2025

Supporting Information File

Metal-Free Synthesis of Tricyclic Benzofuro[2,3-c]pyridin-3-ol Derivatives, Characterization, and Photoluminescence Properties

Authors: Surbhi Mahender Saini, Sandeep Chandrashekharappa*

Affiliation: Department of Medicinal Chemistry, National Institute of Pharmaceutical Education and Research-Raebareli (NIPER-R), Lucknow (UP)-226002, India.

*Corresponding author: **Dr. Sandeep Chandrashekharappa**,
E-mail: c.sandeep@niperraebareli.edu.in and c.sandeepniper@gmail.com
Fax: 91-522-2975587; Tel: 91-522-2499703

Experimental Section:

The starting materials were synthesised using our previously reported method[1]. The required chemicals are received from Sigma-Aldrich and TCI, India, and are used as such without further purification. All the reactions are carried out in hot-air-dried glassware at room temperature. Chemical reactions are monitored on thin-layer chromatography (TLC). TLC are performed on Sigma-Aldrich silica gel 60 F254 on TLC aluminium foils with ethyl acetate and hexane (2:8) as the solvent system and visualisation with a UV-light chamber. Flash chromatography using silica gel (230-400 mesh size) is used for the purification of compounds. NMR spectra are recorded using *Jeol* Nuclear Magnetic Resonance-ECZR series spectrometers, operating at 500 MHz and 125 MHz, respectively, using tetramethyl silane (TMS) as internal standard at ambient temperature, using DMSO-d_6 and $CDCl_3$ as solvents for products. Chemical shift values are measured in δ parts per million. The peak multiplicities are given as follows: s, singlet; d, doublet; dd, double doublet; t, triplet; q, quartet; m, multiplet. *J* values are given in Hertz. Mass spectra are recorded with an AGILENT Mass spectrometer operating on an Agilent Quadrupole Time of Flight (QTOF) detector.

1. **Synthesis of differently substituted 2’-hydroxyethyl cinnamates:**

1.1. General Reaction

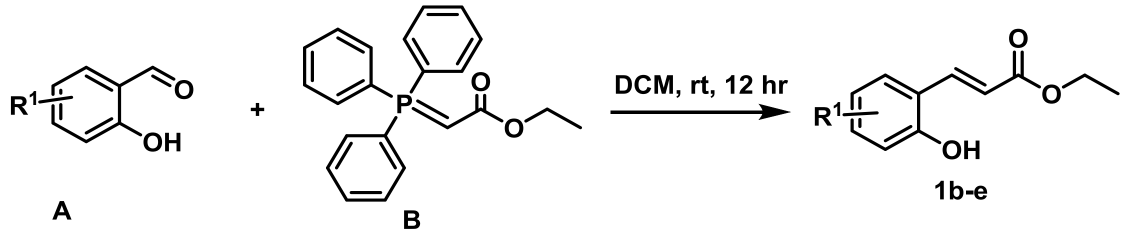

R^1 = H, 5-Br, 5-OMe, 5-NO_2, 4-NEt_2

Scheme 01. Synthesis of differently substituted 2’-hydroxyethyl cinnamates *via* Wittig reaction

1.2. General procedure A. Synthesis of 2’-hydroxyethyl cinnamate analogues

In a hot air oven-dried round bottom flask, the reaction was carried out with 2’-hydroxy-5’-methoxybenzaldehyde (**A**) (2.0 g, 16.40 mmol), ethyl 2-(triphenyl-λ^5-phosphaneylidene) acetate (**B**) (5.7 g, 16.40 mmol, 1 eq.), in DCM (25 mL) as solvent at room temperature for 12 hours. The reaction completion was monitored via TLC. The resulting reaction mixture was dried under reduced pressure and further taken for solvent extraction using ethyl acetate (75 mL) and water (50 mL). The organic content was extracted with ethyl acetate (75 mL×3), washed with water (50 mL×2), dried with anhydrous sodium sulphate and evaporated under reduced pressure to get the crude compound. The crude mixture was purified via column chromatography using 100-200 mesh size silica gel, solvent system ethyl acetate and hexane (1:9) to get 2.9 g (92 % Yield) of **1b.** The other analogues **1c-e** are synthesised using the same protocol.

2. **Synthesis of ethyl 2-(2-benzoyl-2,3-dihydrobenzofuran-3-yl) acetate[1]:**

2.1. General Reaction:

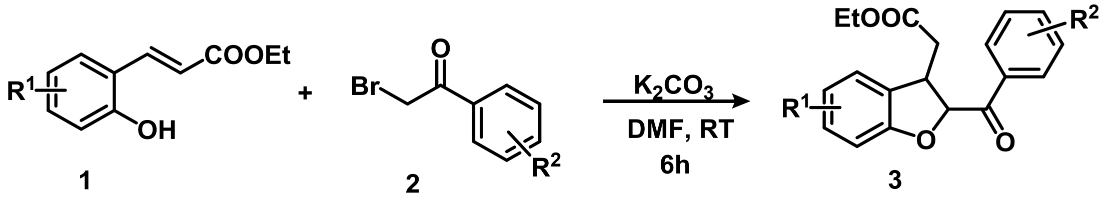

Scheme 02. Synthesis of ethyl 2-(2-benzoyl-2,3-dihydrobenzofuran-3-yl) acetate from differently substituted 2’-hydroxy ethyl cinnamates and different phenacyl bromides.

2.2. General procedure B. Synthesis of ethyl 2-(2-benzoyl-2,3-dihydrobenzofuran-3-yl) acetate analogues (3):

In a hot air oven-dried round bottom flask, the reaction was carried out with 2'-hydroxy cinnamic ethyl ester **1a** (0.20 g, 1.04 mmol, 1 eq.), phenacyl bromide **2a** (0.207 g, 1.04 mmol, 1 eq.), in presence of potassium carbonate (0.317 g, 2.08 mmol, 2.2 eq.) and DMF (5 mL) as solvent at room temperature for 6 hours. The reaction completion was monitored via TLC. The resulting reaction mixture was poured into ice and further taken for solvent extraction using ethyl acetate (75 mL) and water (50 mL). The organic content was extracted with ethyl acetate (25 mL×3), washed with water (25 mL×2), dried with anhydrous sodium sulphate and evaporated under reduced pressure to get the crude compound. The crude compound 0.298 g (92 % yield) of **3a** was used as such without further purification for the synthesis of **4a, 6a**. The remaining derivatives of **3** were synthesised using this same protocol.

3. Synthesis of Ethyl 2-(2-benzoylbenzofuran-3-yl) acetate (4) analogues[1]:

3.1. General Reaction:

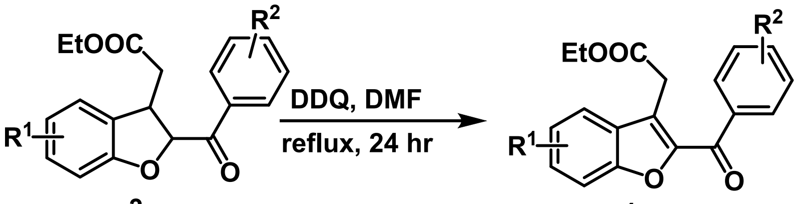

Scheme 03. Synthesis of Ethyl 2-(2-benzoylbenzofuran-3-yl) acetate analogues from differently substituted ethyl 2-(2-benzoyl-2,3-dihydrobenzofuran-3-yl) acetate and DDQ.

3.2. **General procedure C. Synthesis of Ethyl 2-(2-benzoylbenzofuran-3-yl) acetate and analogues:** In a hot air oven dried pressure tube, the reaction was carried out with Ethyl 2-(2-benzoyl-2,3-dihydrobenzofuran-3-yl) acetate **3a** (0.200 g, 0.65 mmol, 1 eq.), DDQ (0.365 g, 1.61 mmol, 2.5 eq.) and DMF (4 mL) as solvent at 150 °C for 24 hours. The completion of reaction was monitored using TLC. The resulting reaction mixture was evaporated under reduced pressure in a rotavapor for the removal of DMF, which gives a crude mixture. The crude mixture was purified via column chromatography using 100-200 mesh size silica gel, solvent system ethyl acetate and hexane to get 0.120g (60 % Yield) of **4a.** The remaining analogues 4**b-g** were synthesised using the same protocol.

4. Synthesis of 4-N substituted analogues of Ethyl 2-(2-benzoyl-2,3-dihydrobenzofuran-3-yl) acetate[2]

4.1. General Reaction:

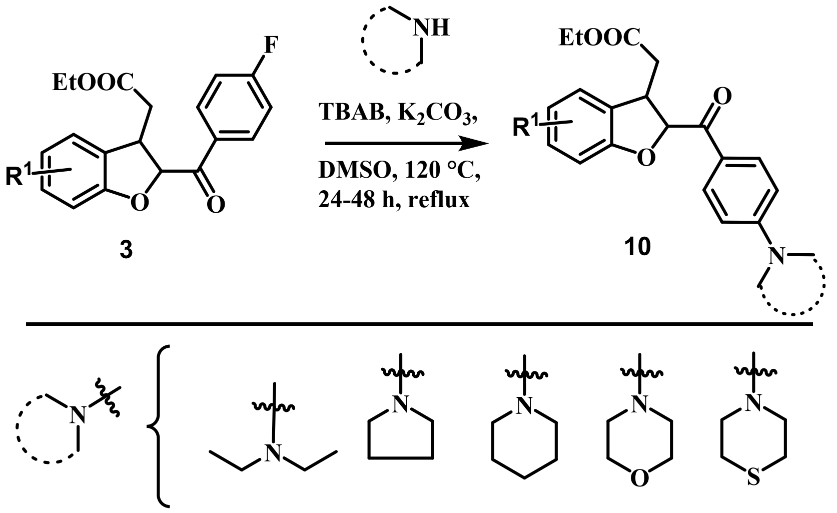

Scheme 04. Synthesis of *p*-*N*-substituted analogues of Ethyl 2-(2-benzoyl-2,3-dihydrobenzofuran-3-yl) acetate via 4-F substitution with secondary amine derivatives[4]

4.2. General procedure D. Synthesis of 4-N substituted analogues of Ethyl 2-(2-benzoyl-2,3-dihydrobenzofuran-3-yl) acetate.

In a hot air oven dried pressure tube, the reaction was carried out with ethyl 2-(2-(4-fluorobenzoyl)-2,3-dihydrobenzofuran-3-yl)acetate (**3b**) (0.105 g, 0.31mmol, 1 eq.), Morpholine (0.027g, 0.31mmol, 1 eq.), tetrabutylammonium bromide (0.011 g, 0.031 mmol, 0.1 eq) in presence of potassium carbonate (0.045 g, 0.31 mmol, 1 eq.) and DMSO (5 mL) as solvent at 100℃ for 48 hours. The reaction completion was monitored via TLC. The resulting reaction mixture was poured into ice and further taken for solvent extraction using ethyl acetate (75 mL) and water (50 mL). The organic content was extracted with ethyl acetate (25 mL×3), washed with water (25 mL×2), dried with anhydrous sodium sulphate and evaporated under reduced pressure to get the crude compound. The crude mixture was purified via column chromatography using 100-200 mesh size silica gel, solvent system ethyl acetate and hexane (2:8) to get 0.090 g (72 % Yield) of **10a.** The other analogues of **10** are synthesised using the same protocol.

5. Synthesis of 1-phenylbenzofuro[2,3-c]pyridin-3-ol from ethyl 2-(2-benzoylbenzofuran-3-yl)acetate

5.1. General Reaction:

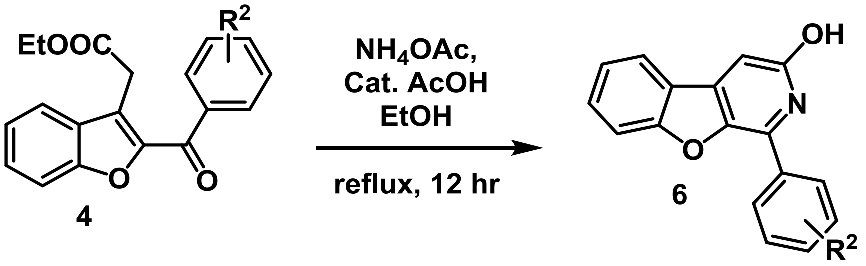

5.2. **General procedure E. Synthesis of 1-phenylbenzofuro[2,3-c]pyridin-3-ol:** A cleaned and hot air-dried pressure tube was charged with ethyl 2-(2-benzoylbenzofuran-3-yl)acetate (**3a**) (100 mg, 0.3 mmol) dissolved in ethanol (4 ml). Immediately, 10 eq. of ammonium acetate (249 mg, 3 mmol) was added to the solution with a catalytic amount of acetic acid (0.1eq) and refluxed for 12 hours. The reaction was monitored via TLC, and after completion, the reaction mixture was transferred to a round-bottom flask with ethanol, and all the solvent was evaporated under reduced pressure using a rotavapor. The crude mixture was purified via flash chromatography using 230-400 mesh size silica gel, solvent system ethyl acetate and hexane (1:9) to get 57 mg, 68% yield of **6a**. The remaining analogues were synthesised using the same protocol.

6. Synthesis of 1-phenylbenzofuro[2,3-c]pyridin-3-ol analogues from ethyl 2-(2-benzoyl-2,3-dihydrobenzofuran-3-yl) acetate analogues

6.1. General Reaction :

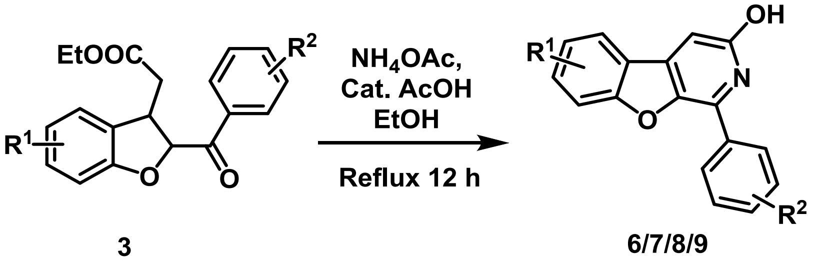

Scheme 05. Synthesis of 1-phenylbenzofuro[2,3-c]pyridin-3-ol analogues from ethyl 2-(2-benzoyl-2,3-dihydrobenzofuran-3-yl) acetate derivatives and Ammonium acetate

6.2. General procedure F. Synthesis of 1-phenylbenzofuro[2,3-c]pyridin-3-ol analogues from ethyl 2-(2-benzoyl-2,3-dihydrobenzofuran-3-yl) acetate analogues:

A cleaned, and hot air-dried pressure tube was charged with ethyl 2-(2-([1,1'-biphenyl]-4-carbonyl)-2,3-dihydrobenzofuran-3-yl)acetate (0.4 g, 1.04 mmol) and dissolved in ethanol (6 mL). Immediately 10 eq. of ammonium acetate (0.800 g, 10.31mmol) was added to the solution with catalytic amount of acetic acid (0.1eq) and refluxed for 12 hours. The reaction

was monitored via TLC and after completion the reaction mixture was transferred to RBF with ethanol and all the solvent was evaporated under reduced pressure using rotavapor. The crude mixture was purified via column chromatography using 100-200 mesh size silica gel, solvent system ethyl acetate and hexane(1:9) to get 0.25g (72 % Yield) of **6g.** The other analogues **6g-h, 7a-7f, 8a, 9a-b** are synthesised using the same protocol.

7. Characterisation details :

Ethyl (*E*)-3-(2-hydroxyphenyl)acrylate (1a)

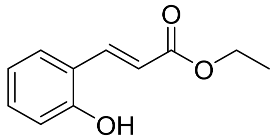

White solid, (2.9g, 92 % yield), ^{1}H NMR (500 MHz, $CDCl_3$) δ 8.04 (d, *J* = 16.1 Hz, 1H), 7.47 (d, *J* = 9.4 Hz, 1H), 7.26 - 7.21 (m, 1H), 6.92 (t, *J* = 7.0 Hz, 1H), 6.85 (d, *J* = 8.1 Hz, 1H), 6.64 (d, *J* = 16.2 Hz, 2H), 4.29 (q, *J* =, 7.1 Hz, 2H), 1.35 (t, *J* = 7.1 Hz, 3H).

Ethyl (*E*)-3-(5-bromo-2-hydroxyphenyl)acrylate (1b)

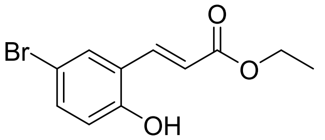

White solid, (2.4g, 89 % yield), ^{1}H NMR (500 MHz, DMSO-d_6) δ 10.57 (s, 1H), 7.81 (d, *J* = 2.6 Hz, 1H), 7.77 (d, *J* = 16.2 Hz, 1H), 7.37 (dd, *J* = 8.8, 2.6 Hz, 1H), 6.87 (d, *J* = 8.8 Hz, 1H), 6.68 (d, *J* = 16.1 Hz, 1H), 4.17 (q, *J* = 7.1 Hz, 2H), 1.25 (t, *J* = 7.1 Hz, 3H).

Ethyl (*E*)-3-(2-hydroxy-5-methoxyphenyl)acrylate (1c)

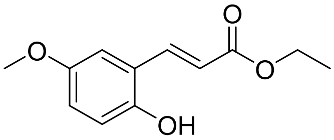

White solid, (2.7g, 93 % yield), ^{1}H NMR (500 MHz, $CDCl_3$) δ 8.05 (d, *J* = 16.4 Hz, 1H), 6.99 (d, *J* = 22.9 Hz, 2H), 6.81 (s, 2H), 6.59 (d, *J* = 17.2 Hz, 1H), 4.28 (q, *J* = 7.1 Hz, 2H), 3.76 (s, 3H), 1.34 (t, *J* = 7.1 Hz, 3H).

Ethyl (*E*)-3-(2-hydroxy-5-nitrophenyl)acrylate (1d)

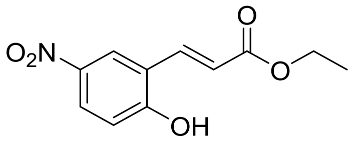

Yellow solid, (2.3g, 81 % yield) ^{1}H NMR (500 MHz, $CDCl_3$) δ 8.40 (s, 1H), 8.14 (d, *J* = 11.7 Hz, 1H), 8.03 (d, *J* = 16.3 Hz, 1H), 7.00 (d, *J* = 9.0 Hz, 1H), 6.79 (d, *J* = 18.0 Hz, 1H), 4.34 (q, *J* = 7.2 Hz, 2H), 1.39 (t, *J* = 7.2 Hz, 3H).

Ethyl (*E*)-3-(4-(diethylamino)-2-hydroxyphenyl)acrylate (1e)

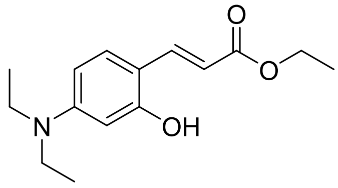

Orange solid**,** (2.6g, 94 % yield), ^{1}H NMR (500 MHz, $CDCl_3$) δ 7.94 (d, *J* = 15.9 Hz, 1H), 7.30 (d, *J* = 8.9 Hz, 1H), 6.37 (d, *J* = 15.9 Hz, 1H), 6.23 (d, *J* = 8.9 Hz, 1H), 6.08 (s, 1H), 4.25 (q, *J* = 7.2 Hz, 2H), 3.33 (q, *J* = 7.1 Hz, 4H), 1.33 (t, *J* = 7.2 Hz, 3H), 1.16 (t, *J* = 7.1 Hz, 6H).

ethyl 2-(2-benzoyl-2,3-dihydrobenzofuran-3-yl)acetate (3a)

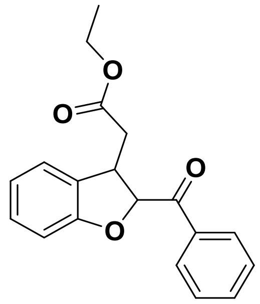

Orange oil, 298 mg, 92% yield, ^{1}H NMR (500 MHz, $CDCl_3$) δ 8.08 (d, *J* = 8.3 Hz, 2H), 7.61 (t, *J* = 6.8 Hz, 1H), 7.50 (t, *J* = 7.7 Hz, 2H), 7.22 - 7.14 (m, 2H), 6.91 (t, *J* = 7.5 Hz, 1H), 6.86 (d, *J* = 8.1 Hz, 1H), 5.73 (d, *J* = 5.8 Hz, 1H), 4.41 - 4.33 (m, 1H), 4.11 (q, *J* = 7.2, 7.1 Hz, 2H), 3.14 - 2.46 (m, 2H), 1.19 (t, *J* = 7.1 Hz, 3H).

ethyl 2-(2-(4-fluorobenzoyl)-2,3-dihydrobenzofuran-3-yl)acetate (3b)

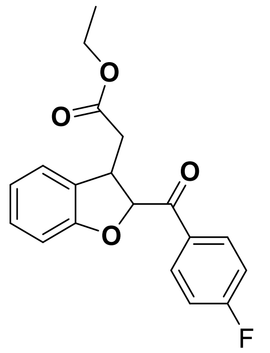

Orange oil, 323mg, 95% yield, ^{1}H NMR (500 MHz, $CDCl_3$) δ 8.13 (dd, J = 8.9, 5.4 Hz, 2H), 7.20 (d, J = 7.4 Hz, 1H), 7.16 (t, J = 8.6 Hz, 3H), 6.91 (t, J = 7.0 Hz, 1H), 6.84 (d, J = 8.0 Hz, 1H), 5.68 (d, J = 5.9 Hz, 1H), 4.43 - 4.35 (m, 1H), 4.11 (q, J =7.2 Hz, 2H), 2.87 (dd, J = 16.0, 5.7 Hz, 1H), 2.75 (dd, J = 16.0, 8.7 Hz, 1H), 1.19 (t, J =7.2 Hz, 3H).

ethyl 2-(2-(4-chlorobenzoyl)-2,3-dihydrobenzofuran-3-yl)acetate (3c)

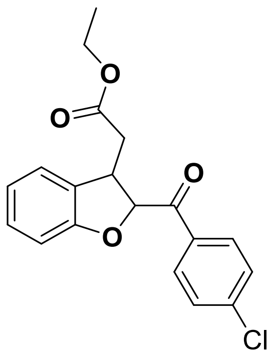

Yellow oil, 340mg, 95% yield, ^{1}H NMR (500 MHz, $CDCl_3$) δ 8.04 (d, J = 8.8 Hz, 2H), 7.47 (d, J = 8.6 Hz, 2H), 7.20 (d, J = 7.5 Hz, 1H), 7.17 (t, J = 7.8, 7.8 Hz, 1H), 6.92 (t, J = 8.0 Hz, 1H), 6.84 (d, J = 7.9 Hz, 1H), 5.67 (d, J = 5.8 Hz, 1H), 4.46 - 4.33 (m, 1H), 4.12 (q, J = 7.1 Hz, 2H), 2.87 (dd, J = 16.0, 5.6 Hz, 1H), 2.75 (dd, J = 16.1, 8.8 Hz, 1H), 1.20 (t, J = 7.1 Hz, 3H).

ethyl 2-(2-(4-bromobenzoyl)-2,3-dihydrobenzofuran-3-yl)acetate (3d)

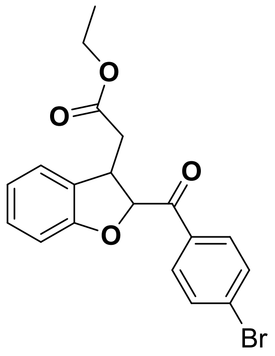

Yellow oil, 390mg, 96% yield, ^{1}H NMR (500 MHz, $CDCl_3$) δ 7.96 (d, J = 8.4 Hz, 2H), 7.64 (d, J = 8.8 Hz, 2H), 7.22 - 7.14 (m, 2H), 6.92 (t, J = 7.5 Hz, 1H), 6.83 (d, J = 7.9 Hz, 1H), 5.66 (d, J = 5.8 Hz, 1H), 4.44 - 4.28 (m, 1H), 4.12 (q, J = 7.1 Hz, 2H), 2.87 (dd, J = 16.1, 5.5 Hz, 1H), 2.75 (dd, J = 16.0, 8.7 Hz, 1H), 1.20 (t, J =7.2 Hz, 3H).

ethyl 2-(2-(4-methylbenzoyl)-2,3-dihydrobenzofuran-3-yl)acetate (3e)

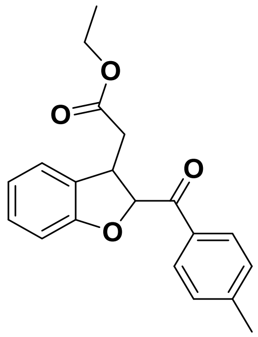

Orange oil, 305mg, 90% yield, ^{1}H NMR (500 MHz, $CDCl_3$) δ 7.98 (d, J = 8.3 Hz, 2H), 7.30 (d, J = 8.3 Hz, 2H), 7.18 (m, 2H), 6.91 (t, J = 7.5Hz, 1H), 6.85 (d, J = 8.1 Hz, 1H), 5.71 (d, J = 5.8 Hz, 1H), 4.38 - 4.32 (m, 1H), 4.11 (q, J = 7.1 Hz, 2H), 2.86 (dd, J = 15.7, 6.0 Hz, 1H), 2.77 (dd, J = 15.9, 8.3 Hz, 1H), 2.43 (s, 3H), 1.19 (t, J =7.1 Hz, 3H).

ethyl 2-(2-([1,1'-biphenyl]-4-carbonyl)-2,3-dihydrobenzofuran-3-yl)acetate (3f)

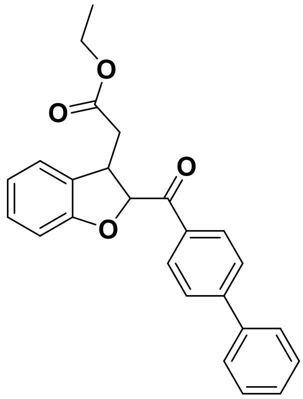

Orange oil, 388mg, 96% yield, ^{1}H NMR (500 MHz, $CDCl_3$) δ 8.16 (d, J = 8.6 Hz, 2H), 7.73 (d, J = 8.6 Hz, 2H), 7.65 (d, J = 8.3 Hz, 2H), 7.49 (t, J = 7.8 Hz, 2H), 7.42 (t, J = 7.3 Hz, 1H), 7.24 - 7.14 (m, 2H), 6.92 (t, J = 7.5 Hz, 1H), 6.87 (d, J = 8.1 Hz, 1H), 5.75 (d, J = 5.8 Hz, 1H), 4.43 - 4.38 (m, 1H), 4.13 (q, J =7.1 Hz, 2H), 2.89 (dd, J = 14.9, 6.0 Hz, 1H), 2.79 (dd, J = 16.0, 8.5 Hz, 1H), 1.20 (t, J = 7.2 Hz, 3H).

ethyl 2-(2-(4-methoxybenzoyl)-2,3-dihydrobenzofuran-3-yl)acetate (3g)

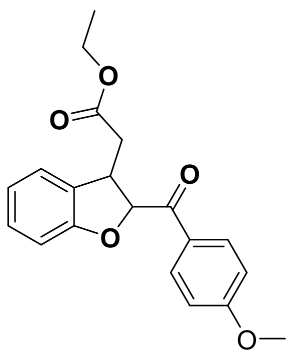

Yellow oil, 320mg, 90% yield, ^{1}H NMR (500 MHz, $CDCl_3$) δ 8.08 (d, *J* = 8.9 Hz, 2H), 7.22 - 7.13 (m, 2H), 6.97 (d, *J* = 8.9 Hz, 2H), 6.90 (t, *J* = 7.1 Hz, 1H), 6.85 (d, *J* = 7.9 Hz, 1H), 5.67 (d, *J* = 5.8 Hz, 1H), 4.41 - 4.32 (m, 1H), 4.11 (q, *J* = 7.1 Hz, 2H), 3.89 (s, 3H), 2.86 (dd, *J* = 15.9, 6.0 Hz, 1H), 2.76 (dd, *J* = 15.9, 8.3 Hz, 1H), 1.19 (t, *J* = 7.2 Hz, 3H).

ethyl 2-(2-(4-morpholinobenzoyl)-2,3-dihydrobenzofuran-3-yl)acetate (10d)

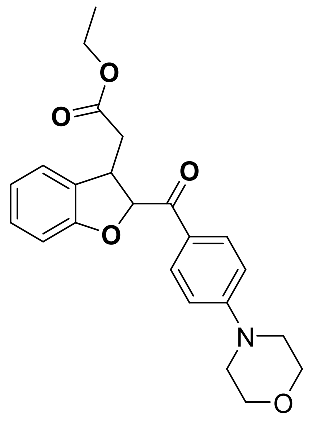

Yellow Solid, 90mg, 72%, ^{1}H NMR (500 MHz, $CDCl_3$) δ 8.00 (d, *J* = 9.0 Hz, 2H), 7.17 (d, *J* = 7.4 Hz, 1H), 7.13 (t, *J* = 7.8, 7.8 Hz, 1H), 6.84 (dd, *J* = 20.1, 8.7 Hz, 4H), 5.63 (d, *J* = 6.0 Hz, 1H), 4.34 (q, *J* = 6.9, 6.9, 6.4 Hz, 1H), 4.09 (q, *J* = 7.2, 7.2, 7.2 Hz, 2H), 3.88 - 3.76 (m, 4H), 3.29 (t, *J* = 4.0, 4.0 Hz, 4H), 2.92 - 2.65 (m, 2H), 1.17 (t, *J* = 7.2, 7.2 Hz, 3H).

ethyl 2-(2-benzoylbenzofuran-3-yl)acetate (4a)

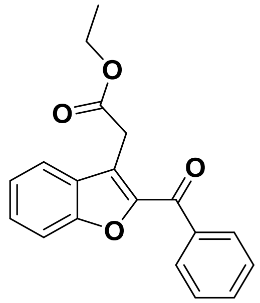

White solid, (0.120 g, 60 % yield) ^1H NMR (500 MHz, $CDCl_3$) δ 8.14 (d, *J* = 8.3 Hz, 2H), 7.71 (d, *J* = 7.9 Hz, 1H), 7.62 (t, *J* = 7.4Hz, 1H), 7.59 - 7.48 (m, 4H), 7.36 (t, *J* = 8.1Hz, 1H), 4.20 (q, *J* = 7.1Hz, 2H), 1.26 (t, *J* = 7.2Hz, 4H). ^{13}C NMR (125 MHz, $CDCl_3$) δ 185.7, 170.1, 154.4, 149.2, 137.4, 133.0, 130.0, 128.5, 128.4, 128.4, 123.8, 123.0, 121.6, 112.5, 61.2, 14.3. HRMS (ESI-TOF)(m/z):$[M+H]^+$calcd for $C_{19}H_{16}O_4$, 309.1126; found 309.1121.

ethyl 2-(2-(4-fluorobenzoyl)benzofuran-3-yl)acetate (4b)

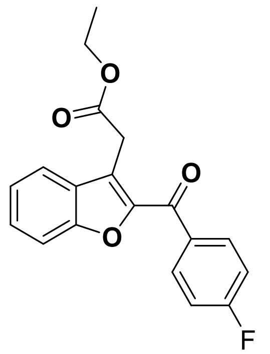

White solid, (0.118 g, 59 % yield) ^1H NMR (500 MHz, DMSO-d_6): δ 8.11 (dd, *J* = 8.7, 5.6 Hz, 2H), 7.88 (d, *J* = 7.9 Hz, 1H), 7.71 (d, *J* = 8.4 Hz, 1H), 7.56 (t, *J* = 7.8 Hz, 1H), 7.43 – 7.37 (m, 3H), 4.18 (s, 2H), 4.06 (q, *J* = 7.1 Hz, 2H), 1.14 (t, *J* = 7.1 Hz, 3H).^{13}C NMR (125 MHz, DMSO-d_6): δ 183.7, 169.9, 154.2, 148.7, 133.8, 133.1, 133.0, 129.4, 128.4, 124.5, 124.1, 122.7, 116.4, 116.2, 112.9, 61.1, 30.4, 14.6. HRMS (ESI-TOF)(m/z):$[M+H]^+$calcd for $C_{19}H_{15}FO_4$, 327.1032;found .327.1159

ethyl 2-(2-(4-chlorobenzoyl)benzofuran-3-yl)acetate (4c)

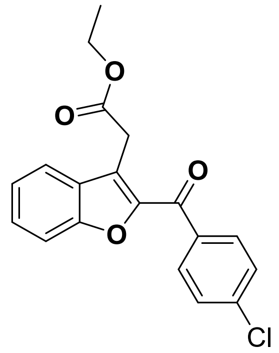

White solid, (0.140 g, 70 % yield) , ^1H NMR (500 MHz, DMSO-d_6): δ 8.03 (d, *J* = 8.4 Hz, 2H), 7.89 (d, *J* = 7.9 Hz, 1H), 7.71 (d, *J* = 8.4 Hz, 1H), 7.65 (d, *J* = 8.4 Hz, 2H), 7.59 – 7.54 (m, 1H), 7.39 (t, *J* = 7.5 Hz, 1H), 4.18 (s, 2H), 4.06 (q, *J* = 7.1 Hz, 2H), 1.14 (t, *J* = 7.1 Hz, 3H).^{13}C NMR (125 MHz, DMSO-d_6): δ 184.1, 169.9, 154.2, 148.7, 138.6, 135.9, 131.8, 129.5, 129.3, 128.4, 124.5, 124.3, 122.7, 112.9, 61.1, 30.4, 14.6. HRMS (ESI-TOF)(m/z):$[M+H]^+$calcd for $C_{19}H_{15}ClO_4$, 343.0737;found 343.0751

ethyl 2-(2-(4-bromobenzoyl)benzofuran-3-yl)acetate (4d)

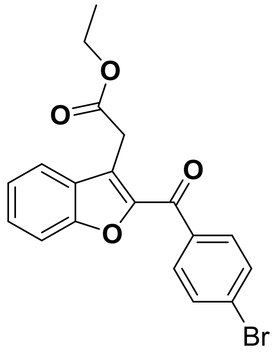

White solid, (0.095 g, 48 % yield) ^{1}H NMR (500 MHz, DMSO-d_6): δ 7.94 (d, J = 8.4 Hz, 2H), 7.88 (d, J = 7.9 Hz, 1H), 7.79 (d, J = 8.3 Hz, 2H), 7.70 (d, J = 8.4 Hz, 1H), 7.57 (t, J = 7.8 Hz, 1H), 7.39 (t, J = 7.5 Hz, 1H), 4.18 (s, 2H), 4.06 (q, J = 7.1 Hz, 2H), 1.14 (t, J = 7.1 Hz, 3H).^{13}C NMR (125 MHz, DMSO-d_6): δ 184.3, 169.9, 154.2, 148.6, 136.2, 132.3, 131.9, 129.5, 128.4, 127.8, 124.5, 124.3, 122.8, 112.9, 61.1, 30.4, 14.6. HRMS (ESI-TOF)(m/z):[M+H]$^+$calcd for $C_{19}H_{15}BrO_4$, 387.0232;found 389.0210

ethyl 2-(2-(4-methylbenzoyl)benzofuran-3-yl)acetate (4e)

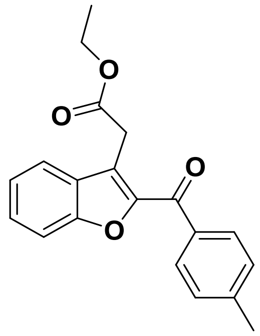

White solid, (0.124 g, 62 % yield), ^{1}H NMR (500 MHz, DMSO-d_6): δ 7.92 (d, J = 8.1 Hz, 2H), 7.86 (d, J = 7.9 Hz, 1H), 7.70 (d, J = 8.4 Hz, 1H), 7.55 (t, J = 7.7 Hz, 1H), 7.38 (m, 3H), 4.16 (s, 2H), 4.06 (q, J = 7.1 Hz, 2H), 2.39 (s, 3H), 1.13 (t, J = 7.1 Hz, 3H).^{13}C NMR (125 MHz, DMSO-d_6): δ 184.9, 170.0, 154.1, 149.1, 144.3, 134.7, 130.1, 129.7, 129.2, 128.5, 124.4, 123.5, 122.6, 112.8, 61, 30.4, 21.7, 14.6 .HRMS (ESI-TOF)(m/z):[M+H]$^+$calcd for$C_{20}H_{18}O_4$, 323.1283;found 323.1289

ethyl 2-(2-([1,1'-biphenyl]-4-carbonyl)benzofuran-3-yl)acetate (4f)

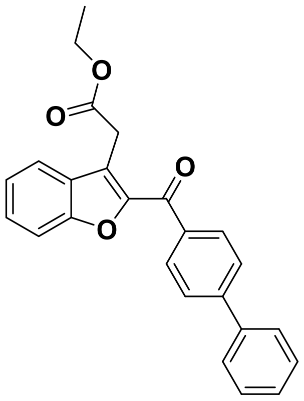

White solid, (0.109g, 55 % yield), ^{1}H NMR (500 MHz, DMSO-d_6): δ 8.11 (d, J = 8.4 Hz, 2H), 7.88 (d, J = 8.4 Hz, 3H), 7.76 (d, J = 7.3 Hz, 2H), 7.73 (d, J = 8.4 Hz, 1H), 7.57 (t, J = 7.8 Hz, 1H), 7.50 (t, J = 7.6 Hz, 2H), 7.40 (dt, J = 11.2, 7.4 Hz, 2H), 4.19 (s, 2H), 4.07 (q, J = 7.1 Hz, 2H), 1.14 (t, J = 7.1 Hz, 3H).^{13}C NMR (125 MHz, DMSO-d_6): δ 184.7, 170.0, 154.2, 149.0, 145.1, 139.3, 136, 130.7, 129.7, 129.3, 129, 128.5, 127.6, 127.4, 124.5, 123.8, 122.7, 112.9, 61.1, 30.4, 14.6. HRMS (ESI-TOF)(m/z):[M+H]$^+$calcd for $C_{25}H_{20}O_4$, 385.1440; found 385.1447.

ethyl 2-(2-(4-methoxybenzoyl)benzofuran-3-yl)acetate (4g)

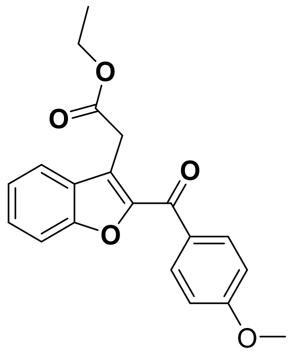

Cream white solid, (0.120 g, 60 % yield), ^{1}H NMR (500 MHz, DMSO-d_6): δ 7.92 (d, J = 8.1 Hz, 2H), 7.86 (d, J = 7.9 Hz, 1H), 7.70 (d, J = 8.4 Hz, 1H), 7.55 (t, J = 7.7 Hz, 1H), 7.38 (m, 3H), 4.16 (s, 2H), 4.06 (q, J = 7.1 Hz, 2H), 2.39 (s, 3H), 1.13 (t, J = 7.1 Hz, 3H).^{13}C NMR (125 MHz, DMSO-d_6): δ 184.9, 170.0, 154.1, 149.1, 144.3, 134.7, 130.1, 129.7, 129.2, 128.5, 124.4, 123.5, 122.6, 112.8, 61.0, 30.4, 21.7, 14.6. HRMS (ESI-TOF)(m/z):[M+H]$^+$calcd for $C_{20}H_{18}O_5$, 338.1154; found 339.1411

1-phenylbenzofuro[2,3-c]pyridin-3-ol (6a)

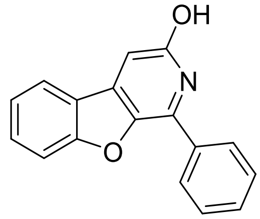

Yellowish white solid, (0.057g, 68 % yield), ^{1}H NMR (500 MHz, $CDCl_3$) δ 8.05 (d, *J* = 7.4 Hz, 2H), 7.93 (d, *J* = 7.4 Hz, 1H), 7.59 (t, *J* = 8.4 Hz, 1H), 7.54-7.50 (m, 3H), 7.46 (t, *J* =7.4 Hz, 1H), 7.35 (t, *J* = 7.5 Hz, 1H), 7.03 (s, 1H). ^{13}C NMR (125 MHz, $CDCl_3$) δ 161.6, 159.3, 141.9, 140.1, 132.0, 131.8, 131.2, 129.8, 128.9, 128.2, 123.3, 122.9, 122.2, 112.3, 103.4. HRMS (ESI-TOF) (m/z): $[M+H]^+$ calcd for $C_{17}H_{11}NO_2$, 262.0868; found 262.0868.

1-(4-fluorophenyl)benzofuro[2,3-c]pyridin-3-ol (6b)

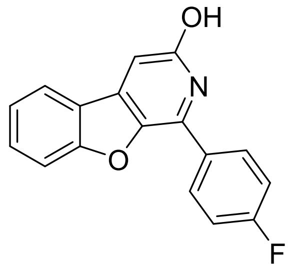

Creamish white solid,(0.071g, 83 % yield), ^{1}H NMR (500 MHz, DMSO-d_6) δ 10.58 (s, 1H), 8.41 (dd, *J* = 9.0, 5.6 Hz, 2H), 8.21 (d, *J* = 7.7 Hz, 1H), 7.77 (d, *J* = 8.3 Hz, 1H), 7.67 (t, *J* = 5.0 Hz 1H), 7.49 - 7.37 (m, 3H), 7.29 (s, 1H). ^{13}C NMR (125 MHz, DMSO-d_6) δ 163.6, 161.7, 158.9, 157.1, 136.2, 130.6, 130.3, 130.2, 123.5, 123, 121.9, 115.8, 115.6, 112.4, 99.2. HRMS (ESI-TOF) (m/z): $[M+H]^+$ calcd for $C_{17}H_{10}FNO_2$, 280.0774; found 280.0774.

1-(4-chlorophenyl)benzofuro[2,3-c]pyridin-3-ol (6c)

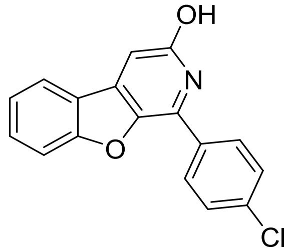

Light yellow solid,(0.070g, 81 % yield), ^{1}H NMR (500 MHz, DMSO-d_6) δ 8.34 (d, *J* = 8.6 Hz, 2H), 8.19 (d, *J* = 7.5 Hz, 1H), 7.75 (d, *J* = 8.4 Hz, 1H), 7.63 (d, *J* = 8.6 Hz, 3H), 7.41 (t, *J* = 7.5 Hz, 1H), 7.31 (s, 1H). ^{13}C NMR (125 MHz, DMSO-d_6) δ 159.4, 157.6, 145.3, 136.8,

135.9, 134.6, 134.4, 131.2, 130.3, 130.1, 129.3, 124.0, 123.5, 122.3, 112.9, 100.3. HRMS (ESI-TOF) (m/z): $[M+H]^+$ calcd for $C_{17}H_{10}ClNO_2$, 296.0478; found 296.0479.

1-(4-bromophenyl)benzofuro[2,3-c]pyridin-3-ol (6d)

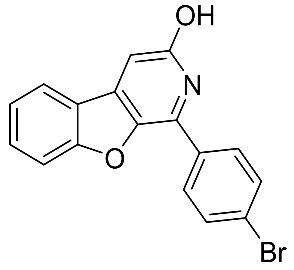

Light Yellow solid, (0.062g, 70 % yield), ^{1}H NMR (500 MHz, DMSO-d_6) δ 10.82 (s, 1H), 8.31 (d, J = 8.4 Hz, 2H), 8.24 (d, J = 7.8 Hz, 1H), 7.83 - 7.77 (m, 3H), 7.68 (t, J = 7.9Hz, 1H), 7.45 (t, J = 7.3Hz, 1H), 7.36 (s, 1H). ^{13}C NMR (125 MHz, DMSO-d_6) δ 159.4, 157.6, 145.3, 136.8, 136, 135, 132.2, 131.2, 130.5, 124, 123.4, 123.3, 122.3, 112.8, 100.2. HRMS (ESI-TOF) (m/z): $[M+H]^+$ calcd for $C_{17}H_{10}BrNO_2$, 339.9973; found 339.9973.

1-(4-methoxyphenyl)benzofuro[2,3-c]pyridin-3-ol (6e)

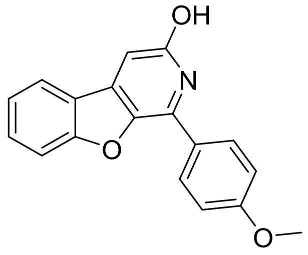

Light yellow solid, (0.060 g, 69 % yield), ^{1}H NMR (500 MHz, $CDCl_3$) δ 8.04 (d, J = 9.0 Hz, 2H), 7.92 (d, J = 7.7 Hz, 1H), 7.60 - 7.55 (m, 1H), 7.50 (d, J = 8.3 Hz, 1H), 7.34 (t, J = 7.4, 7.4 Hz, 1H), 7.05 (d, J = 9.0 Hz, 2H), 6.97 (s, 1H), 3.89 (s, 3H). ^{13}C NMR (125 MHz, $CDCl_3$) δ 162.7, 160.9, 159.4, 140.5, 140.5, 131.2, 131.1, 129.8, 123.4, 123.2, 123.0, 122.2, 114.5, 112.2, 103.4, 55.5. HRMS (ESI-TOF) (m/z): $[M+H]^+$ calcd for $C_{18}H_{13}NO_3$, 292.0973; found 292.0973.

1-(p-tolyl)benzofuro[2,3-c]pyridin-3-ol (6f)

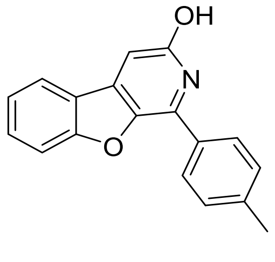

Light yellow solid, (0.057g, 66 % yield), ^{1}H NMR (500 MHz, DMSO-d_6) δ 10.77 (s, 1H), 8.26 (d, J = 8.1 Hz, 2H), 8.21 (d, J = 7.9 Hz, 1H), 7.78 (d, J = 8.3 Hz, 1H), 7.66 (t, J = 7.8 Hz, 1H), 7.44 (t, J = 7.5 Hz, 1H), 7.40 (d, J = 8.4 Hz, 2H), 7.28 (s, 1H), 2.41 (s, 3H). ^{13}C NMR (125 MHz, $CDCl_3$) δ 162.6, 159.4, 141.7, 140.6, 140.3, 131.4, 131, 129.8, 128.1, 123.3, 123.1, 122.1, 112.3, 104.1, 100, 21.6. HRMS (ESI-TOF) (m/z): $[M+H]^+$ calcd for $C_{18}H_{13}NO_2$, 276.1024; found 276.1026.

1-([1,1'-biphenyl]-4-yl)benzofuro[2,3-c]pyridin-3-ol (6g)

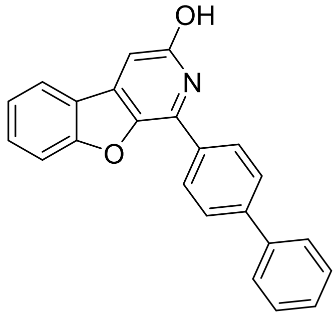

Yellow solid, (0.061g, 69 % yield from method A, 0.25 g, 72% from method B) ^{1}H NMR (500 MHz, $CDCl_3$) δ 8.20 (d, J = 8.0 Hz, 2H), 7.96 (d, J = 7.6 Hz, 1H), 7.75 (d, J = 8.2 Hz, 2H), 7.66 (d, J = 7.3 Hz, 2H), 7.62 - 7.54 (m, 2H), 7.48 (t, J = 7.6 Hz, 2H), 7.38 (dt, J = 14.9, 7.4 Hz, 2H), 7.08 (s, 1H). ^{13}C NMR (125 MHz, $CDCl_3$) δ 161.1, 156, 145.1, 142.6, 140.3, 131.1, 130.8, 128.9, 128.4, 127.9, 127.6, 127.2, 123.3, 122.9, 122.2, 112.3, 103. HRMS (ESI-TOF) (m/z): $[M+H]^+$ calcd for $C_{23}H_{15}NO_2$, 338.1181; found 338.1181.

1-(naphthalen-2-yl)benzofuro[2,3-c]pyridin-3-ol (6h)

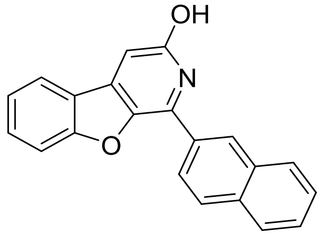

Light yellow solid, (0.055 g, 63 % yield from method A, 0.059g, 68% yield from method B) ^{1}H NMR (500 MHz, DMSO-d_6) δ 8.91 (s, 1H), 8.50 (d, J = 8.6 Hz, 1H), 8.22 (d, J = 7.6 Hz, 1H), 8.10 (d, J = 8.6 Hz, 2H), 8.04 - 7.96 (m, 1H), 7.81 (d, J = 8.0 Hz, 1H), 7.69 (t, J = 7.7 Hz, 1H), 7.64 - 7.57 (m, 2H), 7.46 (t, J = 7.4 Hz, 1H), 7.31 (s, 1H). ^{13}C NMR (125 MHz, DMSO-d_6) δ 159.5, 157.8, 145.6, 143.3, 136.8, 133.8, 133.4, 133.3, 131, 129.2, 128.5, 128.3,

128.1, 127.4, 127, 125.9, 123.9, 123.3, 122.5, 112.8, 99.8. HRMS (ESI-TOF) (m/z): $[M+H]^+$ calcd for $C_{21}H_{13}NO_2$, 312.1024; found 312.1026.

1-(4-(diethylamino)phenyl)benzofuro[2,3-c]pyridin-3-ol (6i)

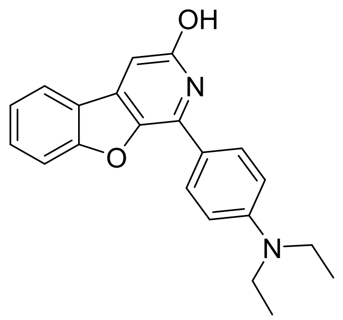

Brown solid, (0.068g, 77 % yield), ^{1}H NMR (500 MHz, DMSO-d_6) δ 10.62 (s, 1H), 8.22 (d, *J* = 6.8 Hz, 2H), 8.17 (d, *J* = 7.8 Hz, 1H), 7.76 (d, *J* = 8.3 Hz, 1H), 7.64 (t, *J* = 7.8 Hz, 1H), 7.42 (t, *J* = 6.2 Hz, 1H), 7.08 (s, 1H), 6.83 (d, *J* = 6.8 Hz, 2H), 3.43 (q, *J* = 7.1, 6.0 Hz, 4H), 1.43 - 0.92 (t, *J* = 7.5 Hz, 6H). ^{13}C NMR (125 MHz, DMSO-d_6) δ 158.9, 157, 148.1, 143.5, 137.4, 135.7, 130.2, 129.4, 123.2, 122.7, 122.2, 121.2, 112.2, 110.9, 96.8, 43.7, 12.5. HRMS (ESI-TOF) (m/z): $[M+H]^+$ calcd for $C_{21}H_{20}N_2O_2$, 333.1603; found 333.1604.

1-(4-(pyrrolidin-1-yl)phenyl)benzofuro[2,3-c]pyridin-3-ol (6*J*)

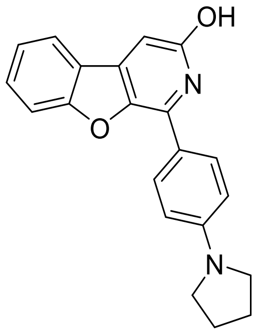

Yellow solid, (0.047 g, 54 % yield), ^{1}H NMR (500 MHz, DMSO-d_6) δ 10.57 (s, 1H), 8.24 (d, *J* = 7.3 Hz, 2H), 8.17 (d, *J* = 7.8 Hz, 1H), 7.76 (d, *J* = 8.3 Hz, 1H), 7.64 (t, *J* = 7.8 Hz, 1H), 7.42 (t, *J* = 7.5 Hz, 1H), 7.08 (s, 1H), 6.71 (d, *J* = 8.9 Hz, 2H), 3.33 (t, *J* = 5.8 Hz, 4H), 1.99 (m, 4H). ^{13}C NMR (125 MHz, DMSO-d_6) δ 158.8, 157.1, 148.2, 143.5, 137.5, 135.8, 130.2, 129.2, 123.2, 122.7, 122.1, 121.4, 112.2, 111.4, 96.8, 79.2, 47.2, 25. HRMS (ESI-TOF) (m/z): $[M+H]^+$ calcd for $C_{21}H_{18}N_2O_2$, 331.1446; found 331.1446.

1-(4-(piperidin-1-yl)phenyl)benzofuro[2,3-c]pyridin-3-ol (6k)

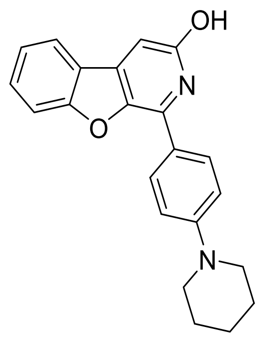

Yellow solid, (0.063g, 72% yield), ^{1}H NMR (500 MHz, DMSO-d_6) δ 8.19 (d, J = 9.0 Hz, 2H), 8.11 (d, J = 7.7 Hz, 1H), 7.69 (d, J = 8.3 Hz, 1H), 7.60 (t, J = 8.5 Hz, 1H), 7.38 (t, J = 7.4 Hz, 1H), 7.06 (s, 1H), 7.04 (d, J = 9.2 Hz, 2H), 3.28 (t, J = 5.4 Hz, 4H), 3.11 - 3.05 (m, 2H), 1.61 (t, J = 4.7 Hz, 4H). ^{13}C NMR (125 MHz, DMSO-d_6) δ 158.6, 156.9, 151.5, 143.6, 136.9, 135.6, 129.9, 129, 126.4, 121.9, 119.3, 114.4, 114.2, 111.9, 99.2, 49.1, 48.4, 24.8. HRMS (ESI-TOF) (m/z): $[M+H]^+$ calcd for $C_{22}H_{20}N_2O_2$, 345.1603; found 345.1604.

1-(4-morpholinophenyl)benzofuro[2,3-c]pyridin-3-ol (6l)

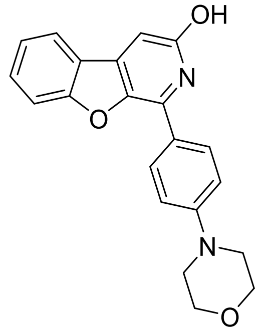

Yellow solid, (0.059g, 68 % yield), ^{1}H NMR (500 MHz, DMSO-d_6) δ 10.70 (s, 1H), 8.27 (d, J = 9.0 Hz, 2H), 8.20 (d, J = 7.7 Hz, 1H), 7.77 (d, J = 8.3 Hz, 1H), 7.65 (t, J = 8.5 Hz, 1H), 7.43 (t, J = 7.5 Hz, 1H), 7.17 (s, 1H), 7.14 (d, J = 9.2 Hz, 2H), 3.77 (t, 4H), 3.25(t, 4H). ^{13}C NMR (125 MHz, DMSO-d_6) δ 159.4, 157.6, 152, 144.5, 137.5, 136.4, 130.8, 129.5, 125.8, 123.7, 123.2, 122.6, 114.8, 112.7, 98.2, 66.5, 48.2. HRMS (ESI-TOF) (m/z): $[M+H]^+$ calcd for $C_{21}H_{18}N_2O_3$, 347.1395; found 347.1395.

1-(4-thiomorpholinophenyl)benzofuro[2,3-c]pyridin-3-ol (6m)

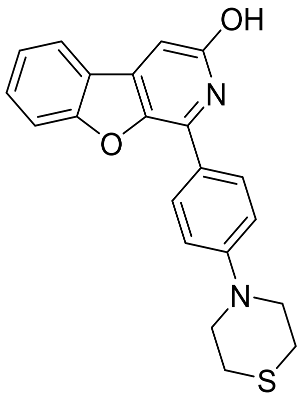

Brown solid, (0.048g, 54 % yield), ^{1}H NMR (500 MHz, DMSO-d_6) δ 10.66 (s, 1H), 8.25 (d, *J* = 8.9 Hz, 2H), 8.19 (d, *J* = 7.5 Hz, 1H), 7.77 (d, *J* = 8.3 Hz, 1H), 7.65 (t, *J* = 7.9 Hz, 1H), 7.43 (t, *J* = 7.5 Hz, 1H), 7.16 (s, 1H), 7.10 (d, *J* = 9.1 Hz, 2H), 3.74 - 3.70 (m, 4H), 2.71 - 2.65 (m, 4H). ^{13}C NMR (125 MHz, DMSO-d_6) δ 158.8, 157, 150.3, 143.9, 135.8, 130.2, 129.2, 124.4, 123.2, 122.7, 122, 114.8, 112.2, 97.5, 50.1, 25. HRMS (ESI-TOF) (m/z): $[M+H]^+$ calcd for $C_{21}H_{18}N_2O_2S$, 363.1167; found 363.1169.

6-methoxy-1-phenylbenzofuro[2,3-c]pyridin-3-ol (7a)

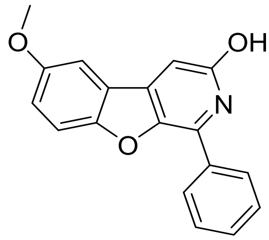

Yellowish white solid, (0.066 g, 77 % yield), ^{1}H NMR (500 MHz, DMSO-d_6) δ 8.34 (d, *J* = 8.4 Hz, 1H), 7.75 (d, *J* = 2.6 Hz, 1H), 7.66 (d, *J* = 8.9 Hz, 1H), 7.58 (t, *J* = 8.4 Hz, 2H), 7.49 (t, *J* = 7.9 Hz, 1H), 7.27 (s, 1H), 7.24 (dd, *J* = 8.9, 2.8 Hz, 1H), 3.89 (s, 3H). ^{13}C NMR (125 MHz, DMSO-d_6) δ 167.9, 159.2, 156.4, 152.5, 146.0, 137.3, 135.8, 129.6, 129.0, 128.6, 123, 119.3, 113.4, 106.1, 99.7, 56.5. HRMS (ESI-TOF) (m/z): $[M+H]^+$ calcd for $C_{18}H_{13}NO_3$, 292.0973; found 292.0974.

1-(4-fluorophenyl)-6-methoxybenzofuro[2,3-c]pyridin-3-ol (7b)

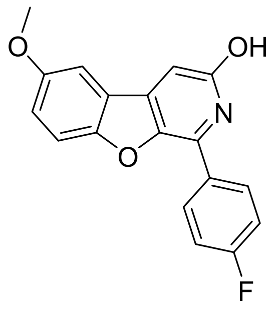

Creamish White solid, (0.070 g, 81 % yield), ^{1}H NMR (500 MHz, DMSO-d_6) δ 10.71 (s, 1H), 8.42 - 8.36 (m, 2H), 7.78 (s, 1H), 7.68 (d, *J* = 8.9 Hz, 1H), 7.42 (t, *J* = 8.9 Hz, 2H), 7.29 (s, 1H), 7.23 (d, *J* = 9.0 Hz, 1H), 3.87 (s, 3H). ^{13}C NMR (125 MHz, DMSO-d_6) δ 163.1 (d, *J* = 247.5 Hz), 159.1, 156.2, 152.3, 145.8, 137, 136.2, 132.3, 130.7 (d, *J* = 8.4 Hz), 122.9, 119.3, 116.1 (d, *J* = 21.7 Hz), 113.4, 105.8, 99.6, 56.4. HRMS (ESI-TOF) (m/z): [M+H$^+$] calcd for $C_{18}H_{12}FNO_3$, 310.0879; found 310.0877.

1-(4-chlorophenyl)-6-methoxybenzofuro[2,3-c]pyridin-3-ol (7c)

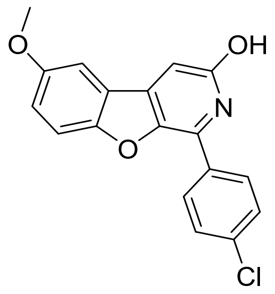

Light yellow solid, (0.072 g, 83 % yield), ^{1}H NMR (500 MHz, DMSO-d_6) δ 8.37 (d, *J* = 8.3 Hz, 2H), 7.76 (s, 1H), 7.64 (m, 3H), 7.30 (s, 1H), 7.25 (d, *J* = 10.5 Hz, 1H), 3.89 (s, 3H). ^{13}C NMR (125 MHz, DMSO-d_6) δ 158.5, 155.7, 151.7, 145.5, 136.5, 135.3, 134.1, 133.8, 129.6, 128.6, 122.3, 118.8, 112.9, 105.3, 99.5, 55.8. HRMS (ESI-TOF) (m/z): [M+H]$^+$ calcd for $C_{18}H_{12}ClNO_3$, 326.0584; found 326.0586.

6-methoxy-1-(4-methoxyphenyl)benzofuro[2,3-c]pyridin-3-ol (7d)

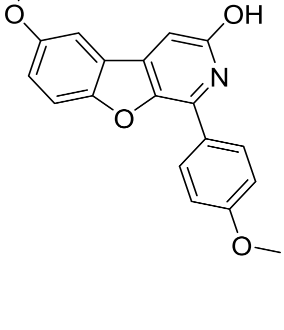

Yellow solid, (0.078g, 90 % yield), ^{1}H NMR (500 MHz, DMSO-d_6) δ 10.65 (s, 1H), 8.31 (d, J = 8.9 Hz, 2H), 7.77 (s, 1H), 7.68 (d, J = 8.9 Hz, 1H), 7.23 (d, J = 9.1 Hz, 1H), 7.21 (s, 1H), 7.15 (d, J = 8.1 Hz, 2H), 3.87 (s, 3H), 3.85 (s, 3H) , ^{13}C NMR (125 MHz, DMSO-d_6) δ 160.0, 158.5, 155.6, 151.7, 129.5, 127.7, 122.5, 118.7, 114, 112.9, 105.2, 98.1, 55.8, 55.2. HRMS (ESI-TOF) (m/z): $[M+H]^+$ calcd for $C_{19}H_{15}NO_4$, 322.1079; found 322.1078.

1-([1,1'-biphenyl]-4-yl)-6-methoxybenzofuro[2,3-c]pyridin-3-ol (7e)

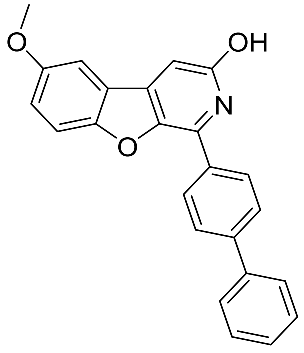

Yellow solid, (0.068 g, 77% yield), ^{1}H NMR (500 MHz, DMSO-d_6) δ 10.49 (s, 1H), 8.46 (t, J = 6.7 Hz, 2H), 7.92 - 7.85 (m, 2H), 7.81 - 7.73 (m, 3H), 7.69 (t, J = 7.9Hz, 1H), 7.51 (q, J = 6.7, 6.3 Hz, 2H), 7.41 (d, J = 7.2 Hz, 1H), 7.31 - 7.23 (m, 2H), 3.90 (s, 3H). ^{13}C NMR (125 MHz, DMSO-d_6) δ 159.2, 156.4, 152.5, 146.2, 141.3, 140.1, 137.0, 134.9, 129.5, 129.1, 128.2, 127.3, 127.2, 123.0, 119.3, 113.4, 106.2, 99.7, 56.6. HRMS (ESI-TOF) (m/z): $[M+H]^+$ calcd for $C_{24}H_{17}NO_3$, 368.1286; found 368.1288.

6-methoxy-1-(naphthalen-2-yl)benzofuro[2,3-c]pyridin-3-ol (7f)

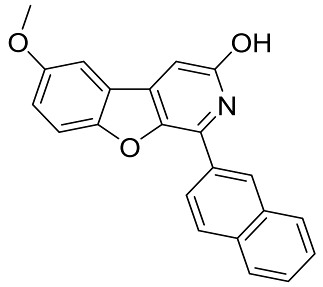

Yellow solid, (0.054 g, 62 % yield), ^{1}H NMR (500 MHz, $CDCl_3$) δ 8.76 (s, 1H), 8.34 (d, J = 7.7 Hz, 1H), 8.02 (s, 1H), 7.99 - 7.94 (m, 1H), 7.88 (s, 1H), 7.63 - 7.54 (m, 3H), 7.29 (s, 1H), 7.18 (s, 1H), 5.96 (d, J = 5.7 Hz, 1H), 3.95 (s, 3H). ^{13}C NMR (125 MHz, $CDCl_3$) δ 171.1, 162.8, 156.9, 146.3, 136.3, 136.1, 134.1, 133, 129.2, 128.7, 128.3, 127.9, 127.8, 126.9, 123.8, 122, 119.9, 116.1, 106.3, 96.4, 91.9, 56. HRMS (ESI-TOF) (m/z): $[M+H]^+$ calcd for $C_{22}H_{15}NO_3$, 342.1130; found 343.0981.

1-(4-(diethylamino)phenyl)-6-methoxybenzofuro[2,3-c]pyridin-3-ol (7g)

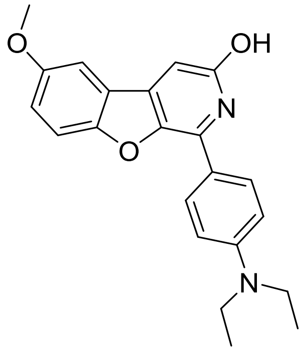

Dark brown solid, (0.063 g, 71 % yield), ^{1}H NMR (500 MHz, $CDCl_3$) δ 7.90 (d, *J* = 9.3 Hz, 2H), 7.36 (d, *J* = 9.0 Hz, 1H), 7.26 (s, 1H), 7.13 (d, *J* = 9.2 Hz, 1H), 6.81 - 6.70 (m, 3H), 3.88 (s, 3H), 3.41 (q, *J* = 7.5 Hz, 4H), 1.22 - 1.17 (t, 6H). ^{13}C NMR (125 MHz, $CDCl_3$) δ 166.8, 164.0, 162.5, 155.9, 154.3, 148.9, 141.2, 139.9, 130.8, 129.2, 122.5, 120, 112.8, 111.5, 105, 101.8, 100, 56.1, 44.5, 12.6. HRMS (ESI-TOF) (m/z): $[M+H]^+$ calcd for $C_{22}H_{22}N_2O_3$, 363.1708; found 363.1707.

6-methoxy-1-(4-(pyrrolidin-1-yl)phenyl)benzofuro[2,3-c]pyridin-3-ol (7h)

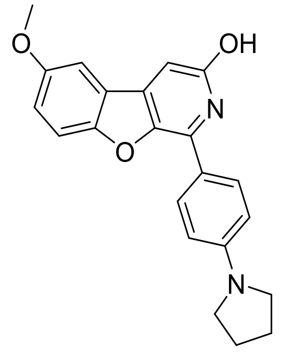

Dark yellow solid, (0.051 g, 58 % yield), ^{1}H NMR (500 MHz, DMSO-*d₆*) δ 8.23 (d, *J* = 9.0 Hz, 2H), 7.69 (s, 1H), 7.63 (d, *J* = 8.9 Hz, 1H), 7.23 (d, *J* = 2.9 Hz, 1H), 7.03 (s, 1H), 6.72 (d, *J* = 9.2 Hz, 2H), 3.89 (s, 3H), 3.36 (d, *J* = 6.1 Hz, 4H), 2.02 (s, 4H). ^{13}C NMR (125 MHz, DMSO-d_6) δ 156.3, 149.0, 129.7, 122.3, 120.0, 118.9, 113.2, 112.0, 106.1, 95.8, 56.6, 47.8, 25.4. HRMS (ESI-TOF) (m/z): $[M+H]^+$ calcd for $C_{22}H_{20}N_2O_3$, 361.1552; found 361.1554.

6-methoxy-1-(4-(piperidin-1-yl)phenyl)benzofuro[2,3-c]pyridin-3-ol (7i)

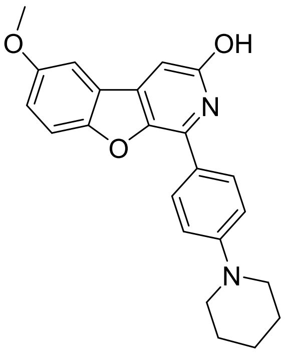

Dark brown solid, (0.069g, 78 % yield)^{1}H NMR (500 MHz, DMSO-*d6*) δ 8.23 (d, *J* = 6.4 Hz, 2H), 7.69 (s, 1H), 7.62 (d, *J* = 8.9 Hz, 1H), 7.22 (d, *J* = 9.0 Hz, 1H), 7.13 - 7.03 (m, 3H), 3.89 (s, 3H), 3.32 (s, 4H), 1.66 (s, 4H), 1.62 (s, 2H). ^{13}C NMR (125 MHz, DMSO-*d6*) δ 158.2, 155.4, 151.5, 151.3, 135.7, 128.7, 126.6, 122.3, 119.7, 118.1, 114.1, 112.3, 105.2, 96.9, 55.7, 48.3, 24.6, 23.5. HRMS (ESI-TOF) (m/z): $[M+H]^+$ calcd for $C_{23}H_{22}N_2O_3$, 375.1708; found 375.1709.

6-methoxy-1-(4-morpholinophenyl)benzofuro[2,3-c]pyridin-3-ol (7*J*)

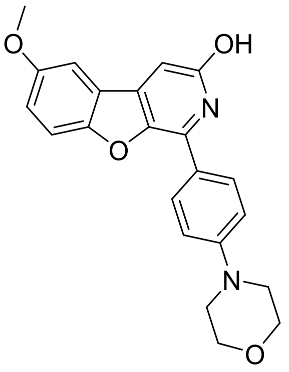

Yellow solid, (0.048g, 70 % yield), ^{1}H NMR (500 MHz, $CDCl_3$) δ 8.01 (d, *J* = 9.0 Hz , 2H), 7.40 (d, *J* = 9.1 Hz, 1H), 7.33 (s, 1H), 7.17 (d, *J* = 9.4 Hz, 1H), 7.04 (d, *J* = 9.0 Hz, 2H), 6.87 (s, 1H), 3.91 (s, 3H), 3.89 (t, 5H), 3.30 (t, 4H). ^{13}C NMR (125MHz, DMSO-*d6*) δ 156.1, 154.7, 152.0, 148.8, 145.5, 133.3, 129.7, 121.6, 121.2, 119.7, 114.5, 113.5, 105.9, 101.8, 64.9, 61.1, 56.1. HRMS (ESI-TOF) (m/z): $[M+H]^+$ calcd for $C_{22}H_{20}N_2O_4$, 377.1501 ; found 377.1503.

6-methoxy-1-(4-thiomorpholinophenyl)benzofuro[2,3-c]pyridin-3-ol (7k)

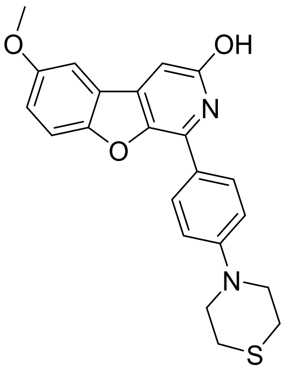

Yellow solid, (0.051 g, 62 % yield), ^{1}H NMR (500 MHz, DMSO-*d_6*) δ 10.56 (s, 1H), 8.24 (d, *J* = 9.0 Hz, 2H), 7.75 (s, 1H), 7.66 (d, *J* = 9.0 Hz, 1H), 7.22 (d, *J* = 11.8 Hz, 1H), 7.14 (s, 1H), 7.09 (d, *J* = 9.2 Hz, 2H), 3.87 (s, 3H), 3.75 - 3.67 (m, 4H), 2.72 - 2.65 (m, 4H). ^{13}C NMR (125 MHz, DMSO-*d_6*) δ 158.1, 155.4, 151.5, 150.2, 144.3, 128.7, 122.2, 118, 114.5, 112.2, 105.2, 96.9, 90.2, 55.7, 49.8, 24.9. HRMS (ESI-TOF) (m/z): $[M+H]^+$ calcd for $C_{22}H_{20}N_2O_3S$, 393.1273; found 393.1272

6-bromo-1-phenylbenzofuro[2,3-c]pyridin-3-ol (8a)

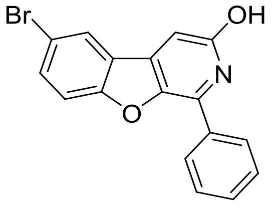

Light orange solid, (0.263 g, 75 % yield), ^{1}H NMR (500 MHz, DMSO-*d_6*) δ 10.85 (s, 1H), 8.53 (d, *J* = 9.0 Hz, 1H), 8.32 (t, *J* = 9.1 Hz, 2H), 7.79 (t, *J* = 9.7 Hz, 2H), 7.58 (d, *J* = 7.2 Hz, 2H), 7.50 (d, *J* = 8.9 Hz, 1H), 7.34 (s, 1H). ^{13}C NMR (125 MHz, DMSO-*d_6*) δ 159, 156, 145.2, 137.2, 135.1, 133, 130.4, 129.4, 128.7, 128.1, 125.6, 124.2, 115.3, 114.5, 99.6. HRMS (ESI-TOF) (m/z): $[M+H]^+$ calcd for $C_{17}H_{10}BrNO_2$, 339.9973; found 339.9974.

7-(diethylamino)-1-phenylbenzofuro[2,3-c]pyridin-3-ol (9a)

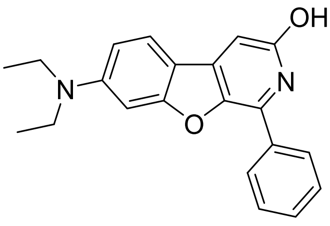

Yellow solid, (0.120 g, 55 % yield), ^{1}H NMR (500 MHz, DMSO-*d_6*) δ 10.41 (s, 1H), 8.30 (d, *J* = 7.6 Hz, 2H), 7.84 (d, *J* = 8.7 Hz, 1H), 7.54 (t, *J* = 7.7 Hz, 2H), 7.46 (t, *J* = 7.1 Hz, 1H),

6.92 (s, 1H), 6.85 (s, 1H), 6.78 (d, J = 9.0 Hz, 1H), 3.56 - 3.36 (m, 4H), 1.19 (t, J = 7.0 Hz, 6H). ^{13}C NMR (125 MHz, DMSO-d_6) δ 161.1, 159.6, 151.2, 129.2, 128.8, 128.5, 123.5, 110.3, 109.5, 98.1, 93.8, 44.8, 12.9. HRMS (ESI-TOF) (m/z): $[M+H]^+$ calcd for $C_{21}H_{20}N_2O_2$, 333.1603; found 333.1605.

7-(diethylamino)-1-(4-fluorophenyl)benzofuro[2,3-c]pyridin-3-ol (9b)

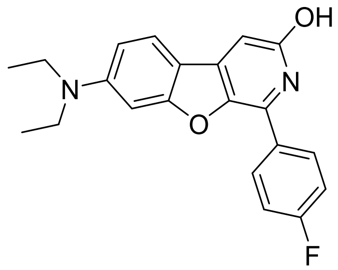

Yellow crystalline solid, (0.141g, 64 % yield), ^{1}H NMR (500 MHz, DMSO-d_6) δ 10.53 (s, 1H), 8.35 (t, J = 6.7 Hz, 2H), 7.86 (d, J = 8.9 Hz, 1H), 7.43 - 7.37 (m, 2H), 6.97 (s, 1H), 6.86 (s, 1H), 6.77 (d, J = 9.0 Hz, 1H), 3.46 (q, J = 6.9 Hz, 4H), 1.15 (t, J = 7.0 Hz, 6H). ^{13}C NMR (125 MHz, DMSO-$d6$) δ 163.9, 161.9, 160.9, 159.5, 150.7, 144.6, 138, 134, 132.3, 130.6 (d, J = 8.5 Hz), 123.8, 115.9 (d, J = 21.7 Hz), 109.7, 109.1, 98, 93.2, 44.8, 12.8. HRMS (ESI-TOF) (m/z): $[M+H]^+$ calcd for $C_{21}H_{19}FN_2O_2$, 351.1509; found 351.1509.

7-(diethylamino)-1-(4-(piperidin-1-yl)phenyl)benzofuro[2,3-c]pyridin-3-ol (9c)

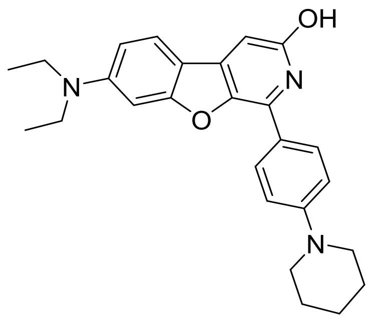

Dark brown solid, (0.066 g, 74 % yield), ^{1}H NMR (500 MHz, $CDCl_3$) δ 7.85 (d, J = 8.9 Hz, 2H), 7.62 (d, J = 8.6 Hz, 1H), 7.03 (d, J = 9.0 Hz, 2H), 6.65 - 6.55 (m, 3H), 3.45 (q, J = 7.2, 6.9 Hz, 4H), 3.34 - 3.30 (m, 4H), 1.71 (s, 4H), 1.65 (s, 2H), 1.23 (d, J = 7.2 Hz, 6H). ^{13}C NMR (125 MHz, $CDCl_3$) δ 162.8, 162.4, 152.5, 151.4, 142.2, 139.8, 128.4, 127.8, 123.6, 119.7, 115.5, 115.1, 109.9, 108.7, 100.2, 93.1, 49.3, 45, 25.5, 14, 12.5. HRMS (ESI-TOF) (m/z): $[M+H]^+$ calcd for $C_{26}H_{29}N_3O_2$, 416.2338; found 416.2338.

6-nitro-1-phenyl-3,4-dihydrobenzofuro[2,3-c]pyridin-3-ol (IIIa)

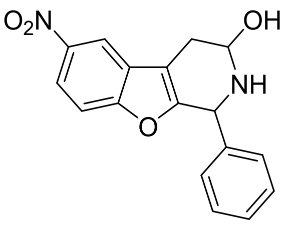

Crystalline white solid,^{1}H NMR (500 MHz, DMSO-d_6) δ 8.65 - 8.58 (m, 2H), 8.23 - 8.15 (m, 1H), 7.76 (dd, *J* = 9.1, 3.9 Hz, 1H), 7.41 - 7.39 (m, 3H), 7.35 (dd, *J* = 6.2, 5.7, 2.8 Hz, 1H), 5.90 - 5.87 (m, 1H), 3.91 - 3.55 (m, 2H). ^{13}C NMR (125 MHz, DMSO-d_6) δ 167.1, 165.8, 162.2, 157.8, 152.3, 143.7, 139.7, 128.8, 128.4, 127.3, 127.2, 120.3, 116.4, 112.2, 110.5, 107.1, 54.7, 27.6.

6-methoxy-1-(p-tolyl)-4a,9a-dihydrobenzofuro[2,3-c]pyridin-3-ol (IIIb)

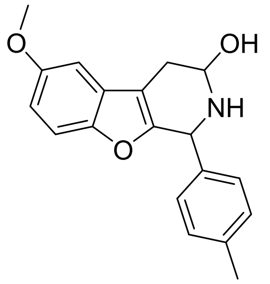

Cream white solid,^{1}H NMR (500 MHz, DMSO-d_6) δ 8.50 (s, 1H), 7.36 (d, *J* = 8.9 Hz, 1H), 7.23 (d, *J* = 8.1 Hz, 2H), 7.18 (d, *J* = 8.1 Hz, 2H), 7.11 (d, *J* = 2.6 Hz, 1H), 6.84 (dd, *J* = 9.0, 2.7 Hz, 1H), 5.72 (s, 1H), 3.78 (s, 3H), 3.67 (dd, *J* = 20.9, 3.6 Hz, 1H), 3.54 (dd, *J* = 20.8, 3.6 Hz, 1H), 2.28 (s, 3H). ^{13}C NMR (125 MHz, DMSO-d_6) δ 167.6, 155.7, 149.8, 149.6, 137.5, 129.2, 127.2, 127.1, 112.6, 111.6, 109.1, 102.5, 55.6, 54.7, 27.9, 20.6.

8. Spectral details :

1. 1-phenylbenzofuro[2,3-c]pyridin-3-ol (6a)

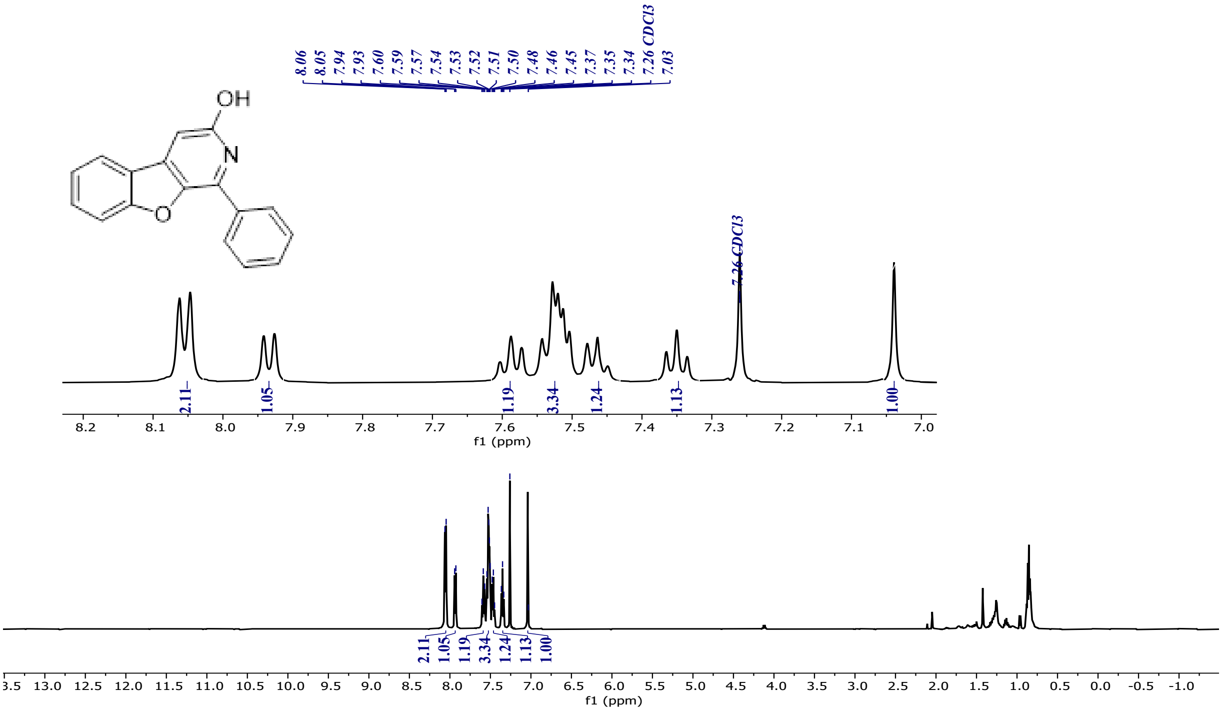

Figure 1. ^{1}H NMR (500 MHz, $CDCl_3$)spectrum of 1-phenylbenzofuro[2,3-c]pyridin-3-ol (**6a**).

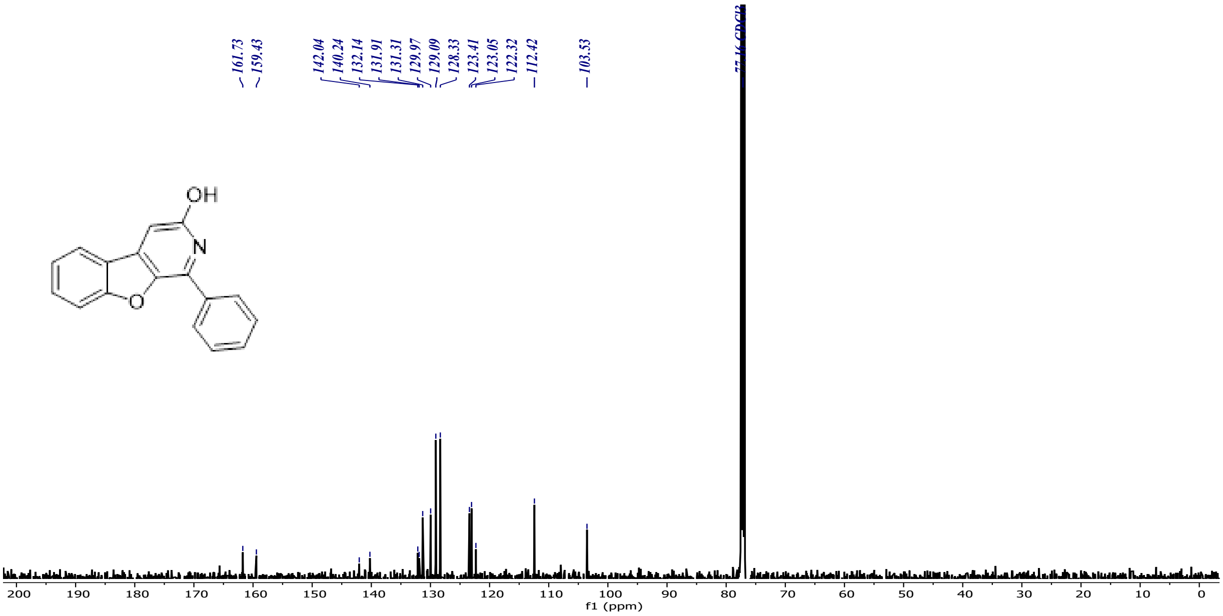

Figure 2. ^{13}C NMR (125 MHz, $CDCl_3$) spectrum of 1-phenylbenzofuro[2,3-c]pyridin-3-ol (**6a**).

2. 1-(4-fluorophenyl)benzofuro[2,3-c]pyridin-3-ol (6b)

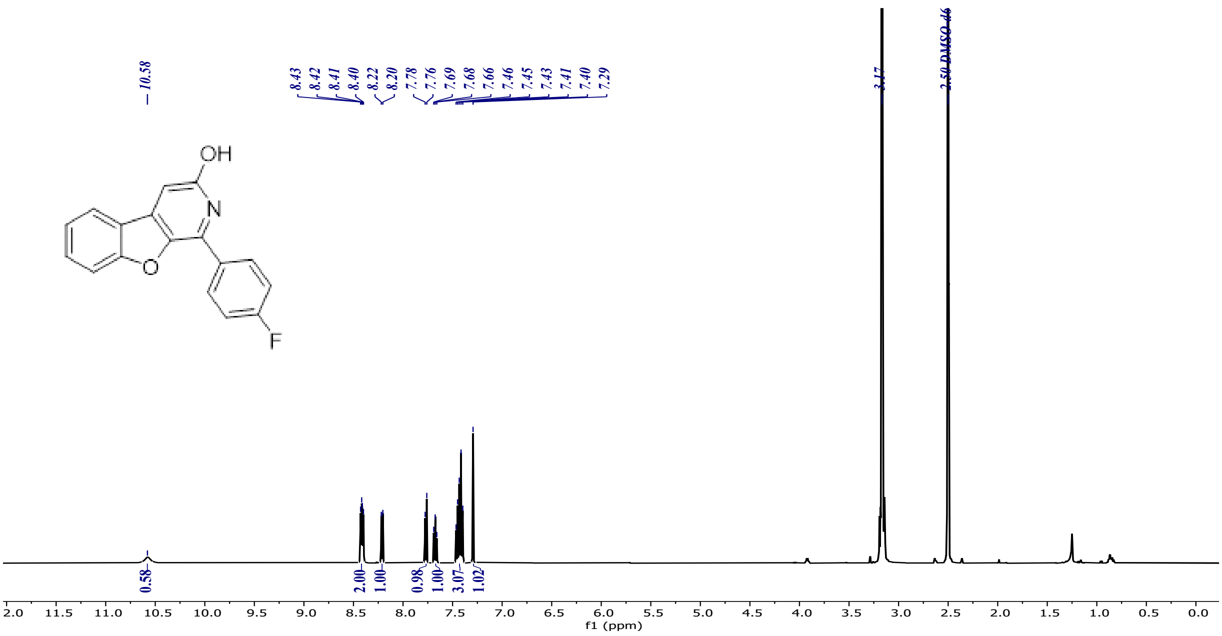

Figure 3. ^{1}H NMR (500 MHz, DMSO-d_6) spectrum of 1-(4-fluorophenyl)benzofuro[2,3-c]pyridin-3-ol (**6b**)

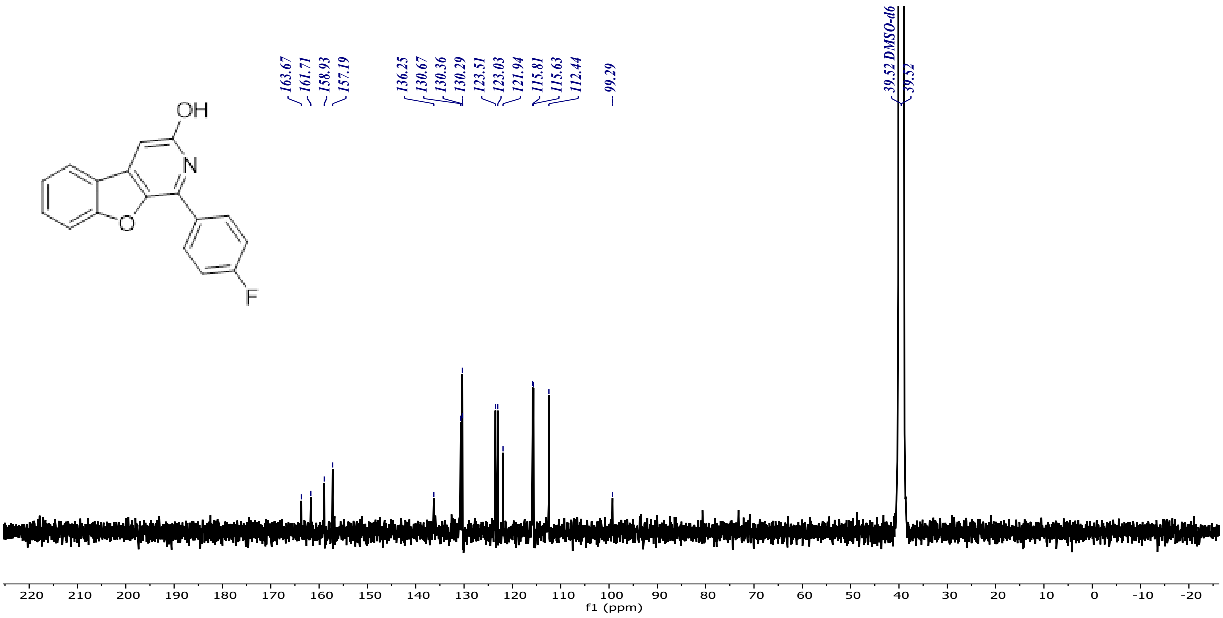

Figure 4. ^{13}C NMR (125 MHz, DMSO-d_6) spectrum of 1-(4-fluorophenyl)benzofuro[2,3-c]pyridin-3-ol (**6b**)

3. 1-(4-chlorophenyl)benzofuro[2,3-c]pyridin-3-ol (6c)

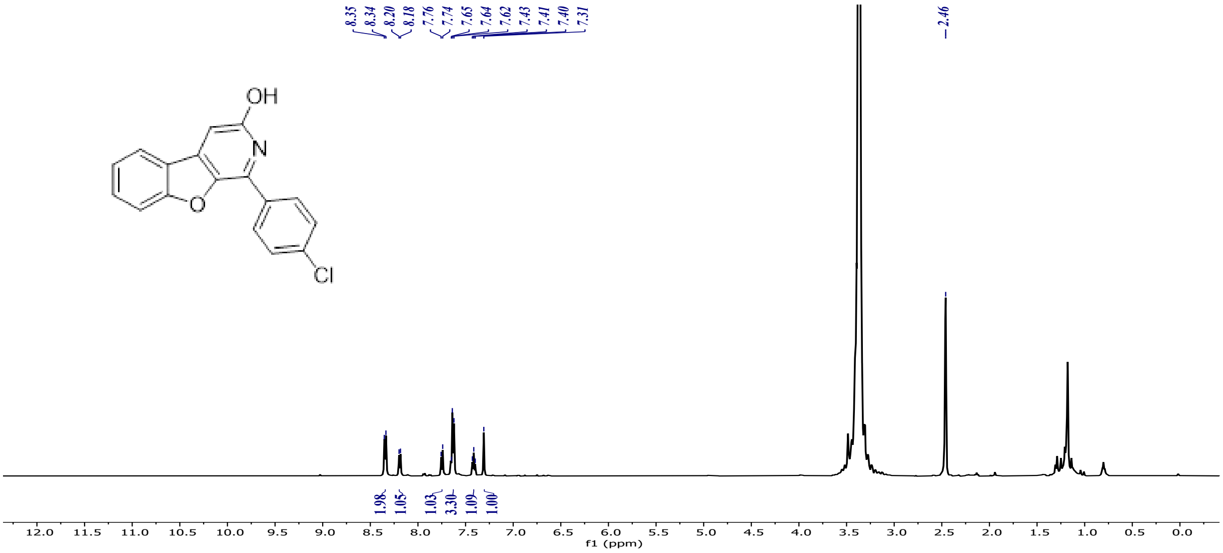

Figure 5. ^{1}H NMR(500 MHz, DMSO-d_6) spectrum of 1-(4-chlorophenyl)benzofuro[2,3-c]pyridin-3-ol (**6c**)

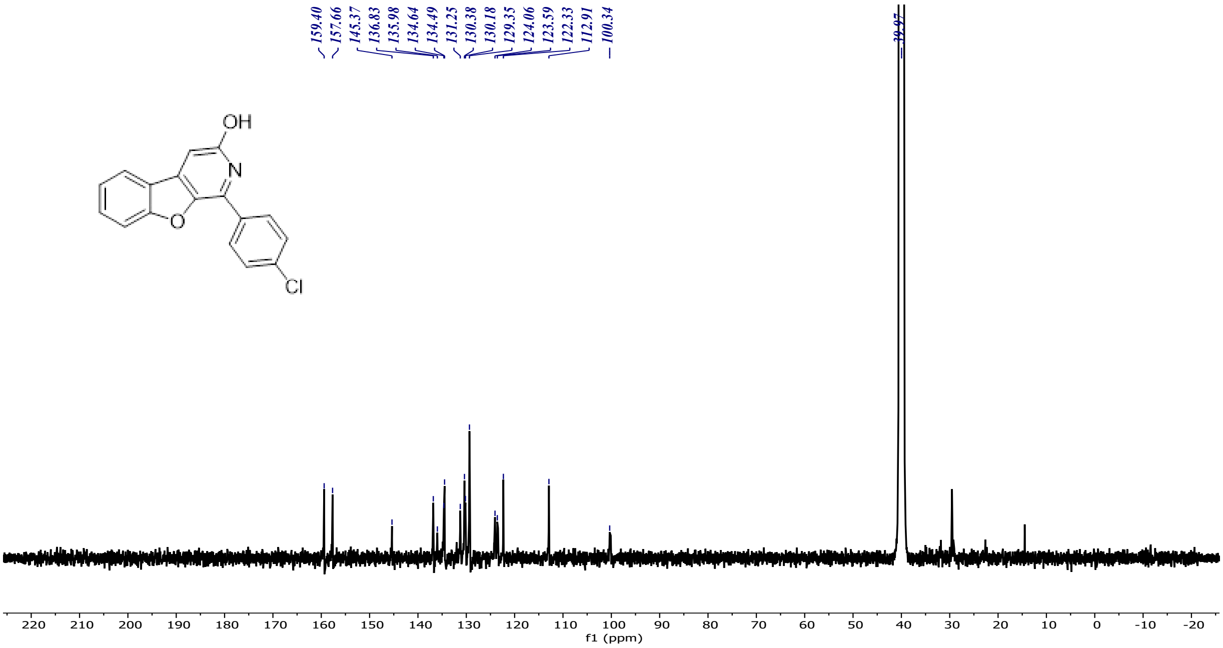

Figure 6. ^{13}C NMR (125 MHz, DMSO-d_6) spectrum of 1-(4-chlorophenyl)benzofuro[2,3-c]pyridin-3-ol (**6c**)

4. 1-(4-bromophenyl)benzofuro[2,3-c]pyridin-3-ol (6d)

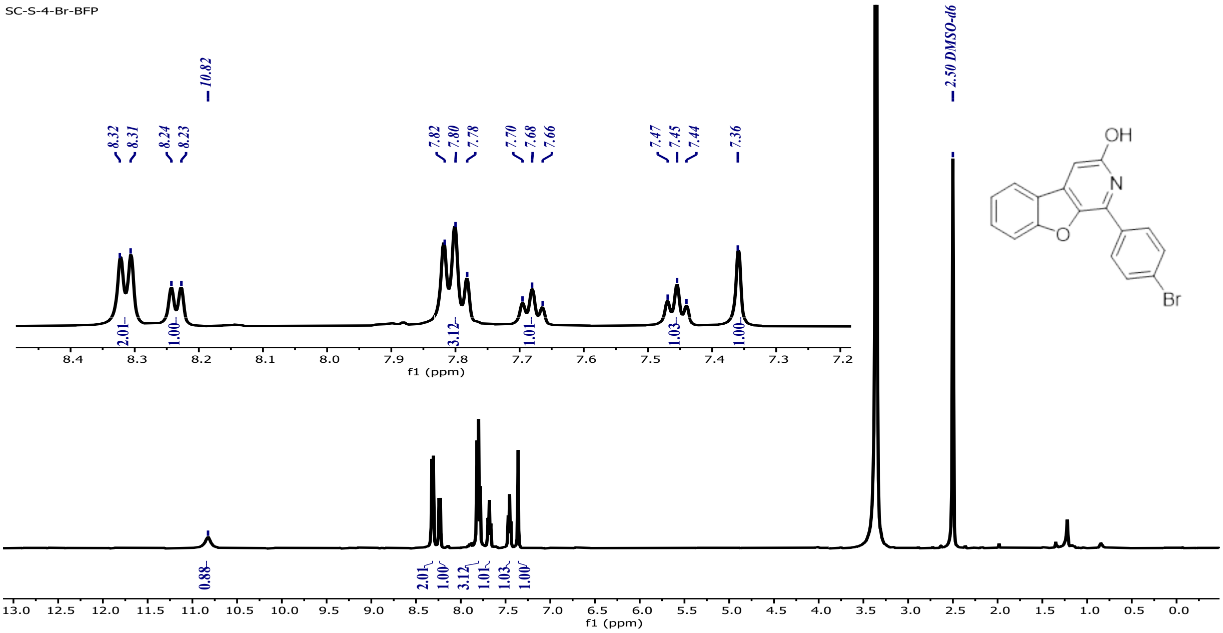

Figure 7. ¹H NMR (500 MHz, DMSO-d_6) spectrum of 1-(4-bromophenyl)benzofuro[2,3-c]pyridin-3-ol (**6d**)

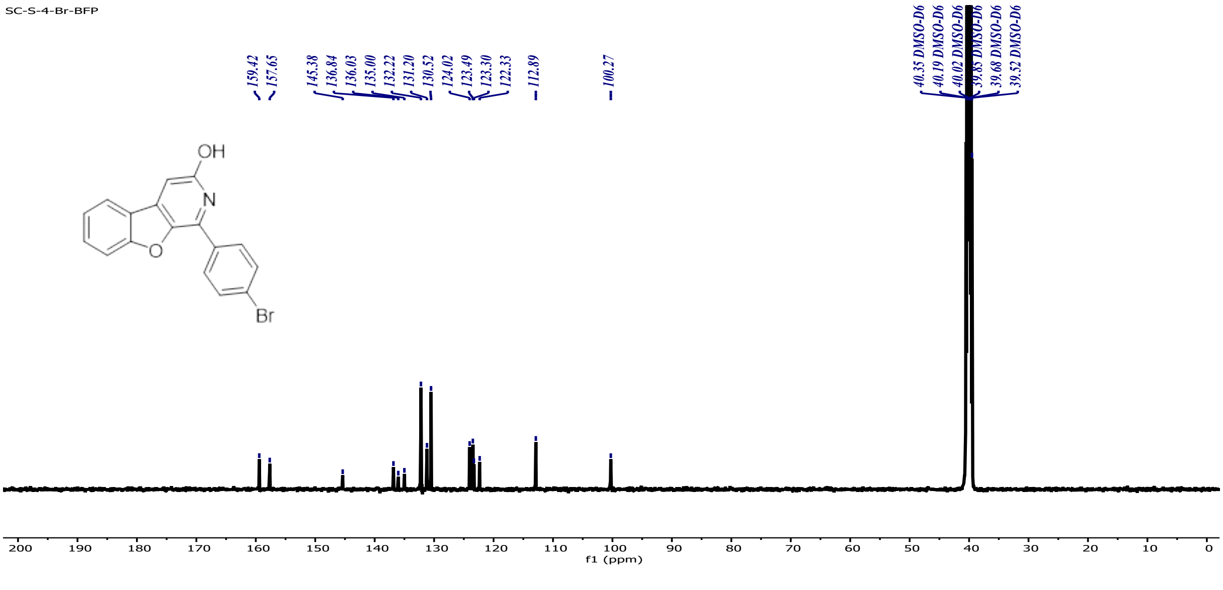

Figure 8. ^{13}C NMR(125 MHz, DMSO-d_6) spectrum of 1-(4-bromophenyl)benzofuro[2,3-c]pyridin-3-ol (**6d**)

5. 1-(4-methoxyphenyl)benzofuro[2,3-c]pyridin-3-ol (6e)

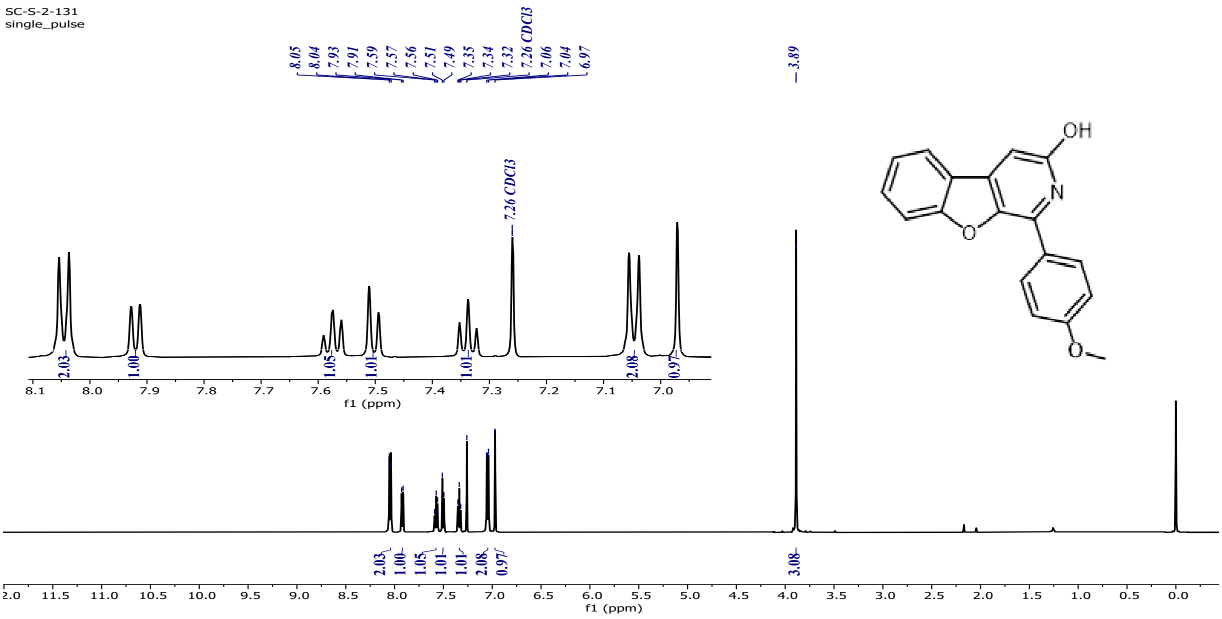

Figure 9. ^{1}H NMR (500 MHz, $CDCl_3$) spectrum of 1-(4-methoxyphenyl)benzofuro[2,3-c]pyridin-3-ol (**6e**)

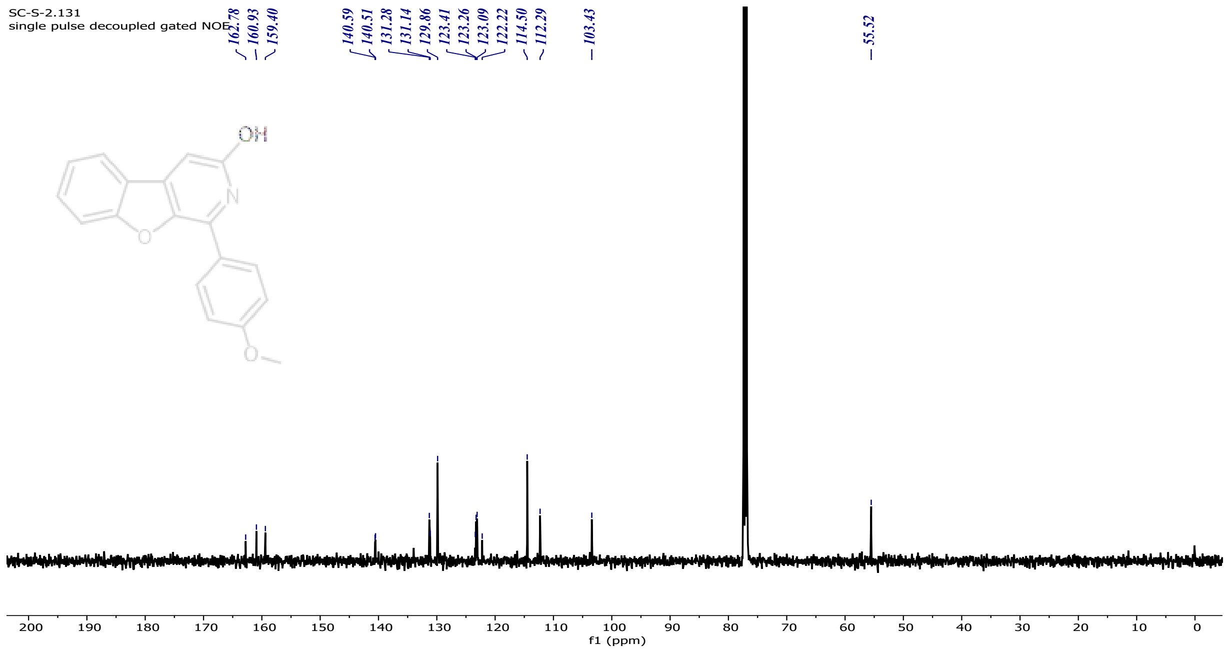

Figure 10. ^{13}C NMR (125 MHz, $CDCl_3$)spectrum of 1-(4-methoxyphenyl)benzofuro[2,3-c]pyridin-3-ol (**6e**)

6. 1-(p-tolyl)benzofuro[2,3-c]pyridin-3-ol (6f)

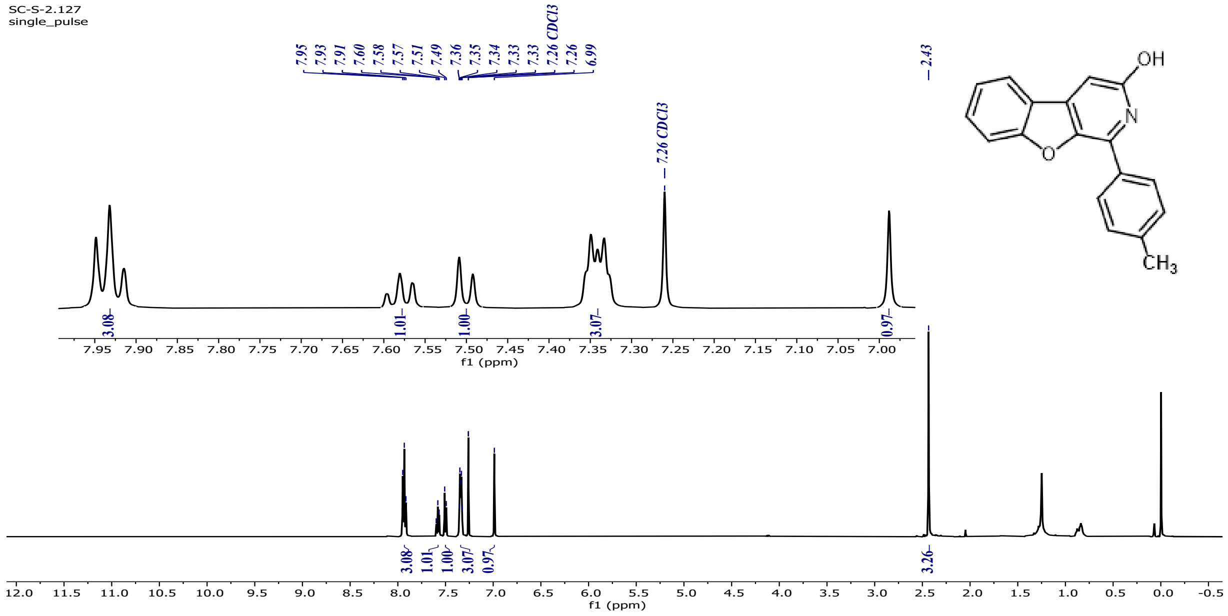

Figure 11. ^{1}H NMR (500 MHz, $CDCl_3$)spectrum of 1-(p-tolyl)benzofuro[2,3-c]pyridin-3-ol (**6f**).

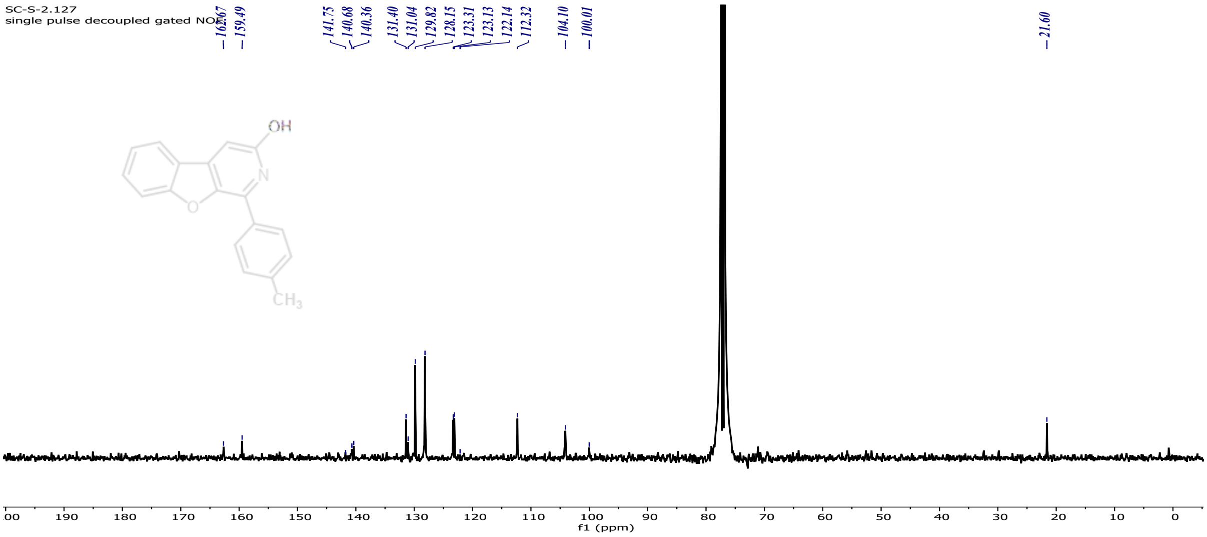

Figure 12. ^{13}C NMR (125 MHz, $CDCl_3$)spectrum of 1-(p-tolyl)benzofuro[2,3-c]pyridin-3-ol (**6f**).

7. 1-([1,1'-biphenyl]-4-yl)benzofuro[2,3-c]pyridin-3-ol (6g)

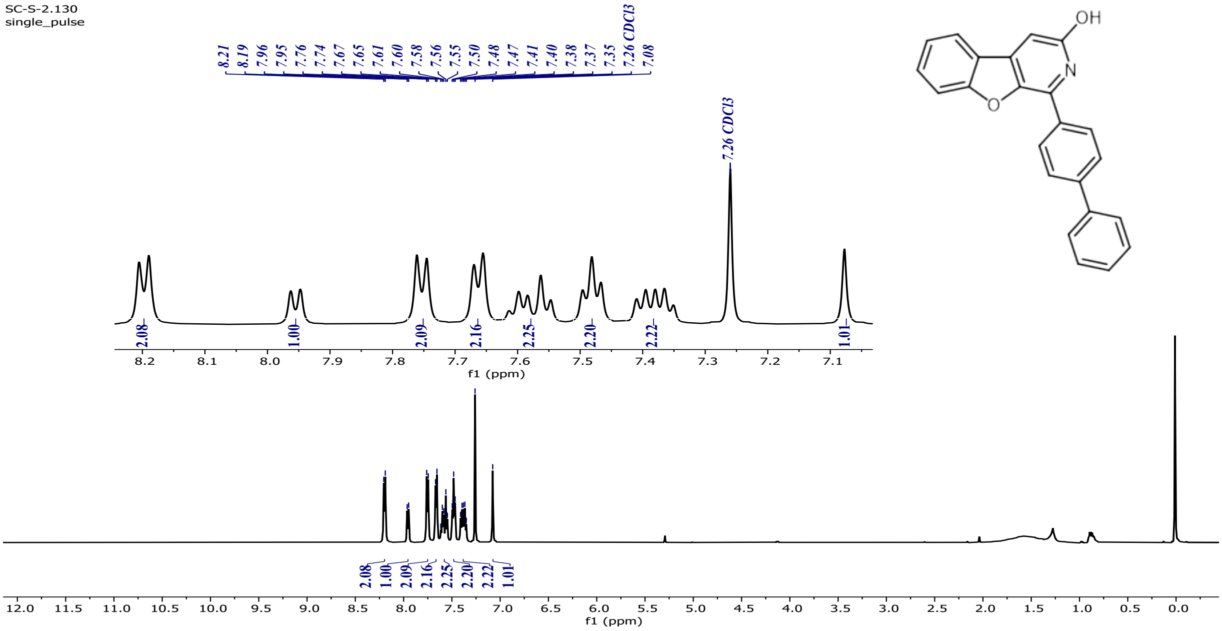

Figure 13. ^{1}H NMR (500 MHz, $CDCl_3$)spectrum of 1-([1,1'-biphenyl]-4-yl)benzofuro[2,3-c]pyridin-3-ol. (**6g**)

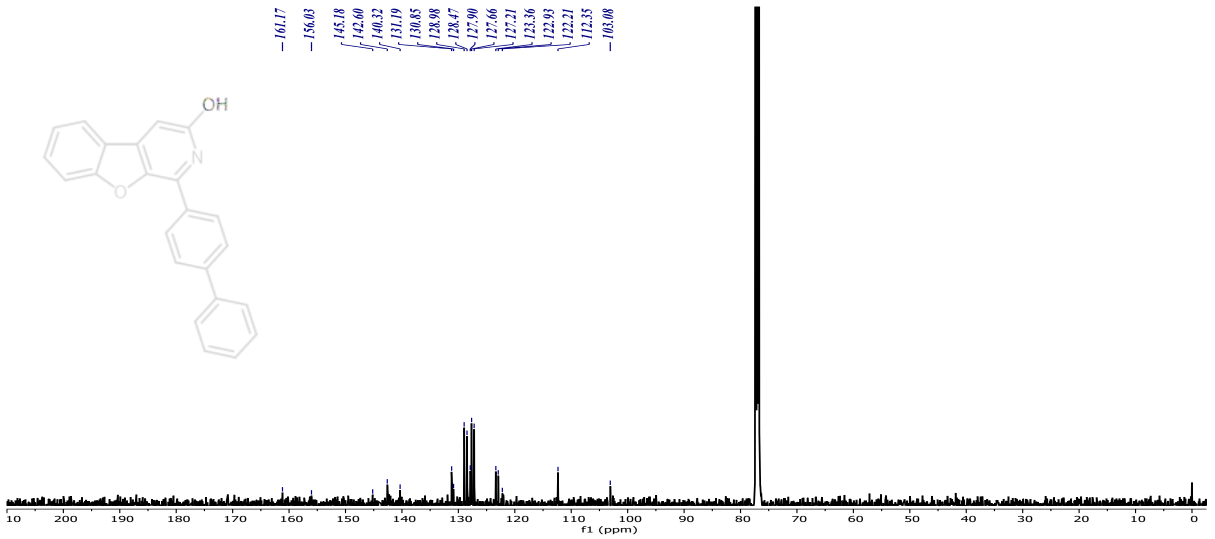

Figure 14. ^{13}C NMR (125 MHz, $CDCl_3$)spectrum of 1-([1,1'-biphenyl]-4-yl)benzofuro[2,3-c]pyridin-3-ol. (**6g**)

8. 1-(naphthalen-2-yl)benzofuro[2,3-c]pyridin-3-ol (6h)

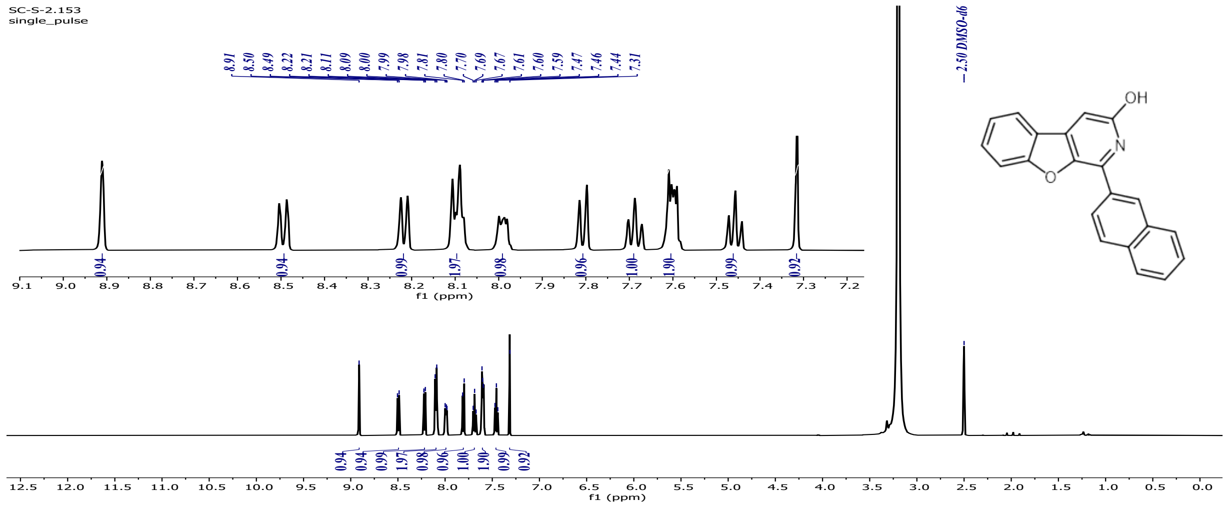

Figure 15. ^{1}H NMR (500 MHz, DMSO-d_6) spectrum of 1-(naphthalen-2-yl)benzofuro[2,3-c]pyridin-3-ol. (**6h**)

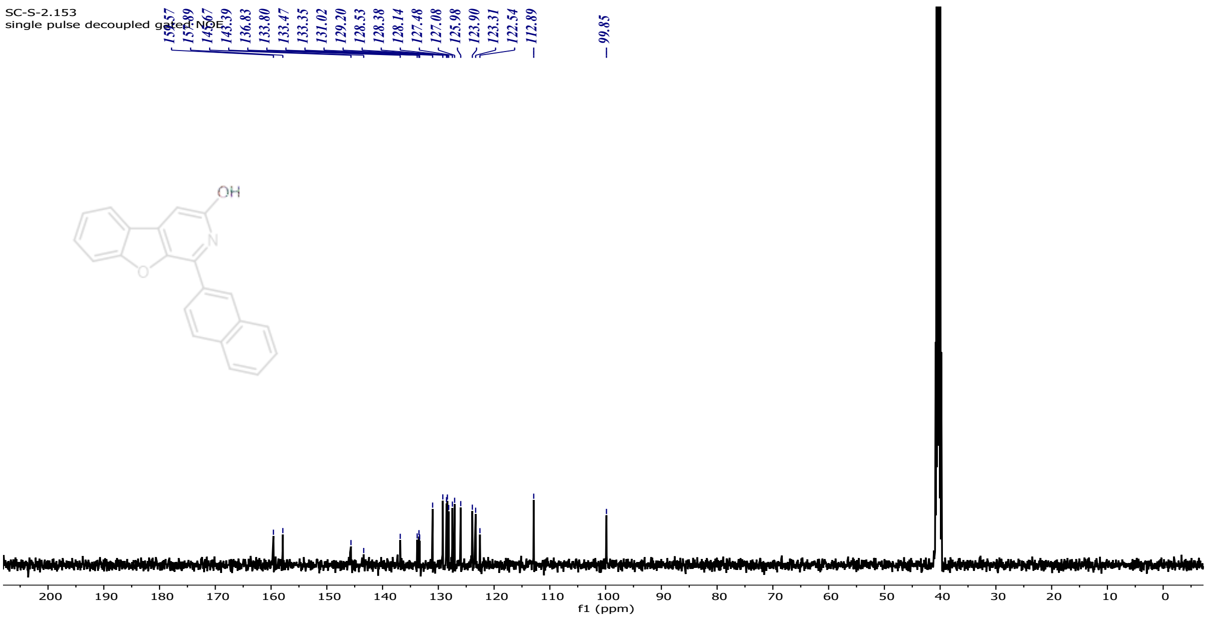

Figure 16. ^{13}C NMR (125 MHz, DMSO-d_6) spectrum of 1-(naphthalen-2-yl)benzofuro[2,3-c]pyridin-3-ol. (**6h**)

9. 1-(4-(diethylamino)phenyl)benzofuro[2,3-c]pyridin-3-ol (6i)

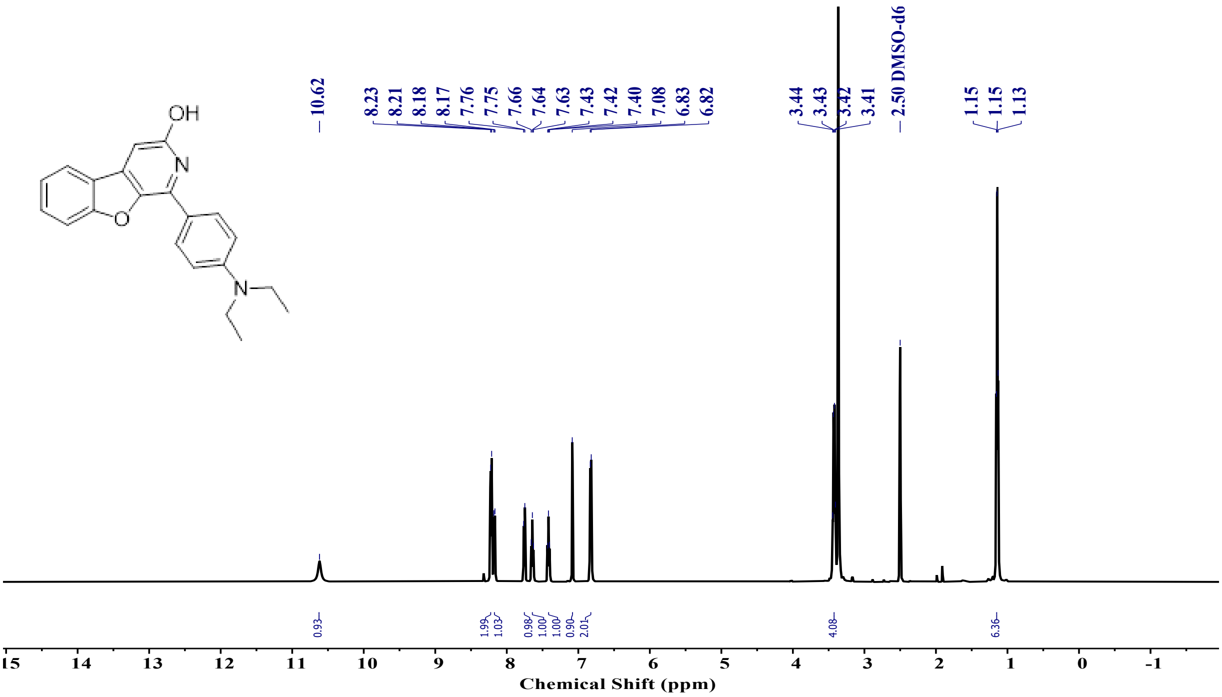

Figure 17. ^1H NMR(500 MHz, DMSO-d_6) spectrum of 1-(4-(diethylamino)phenyl)benzofuro[2,3-c]pyridin-3-ol (**6i**)

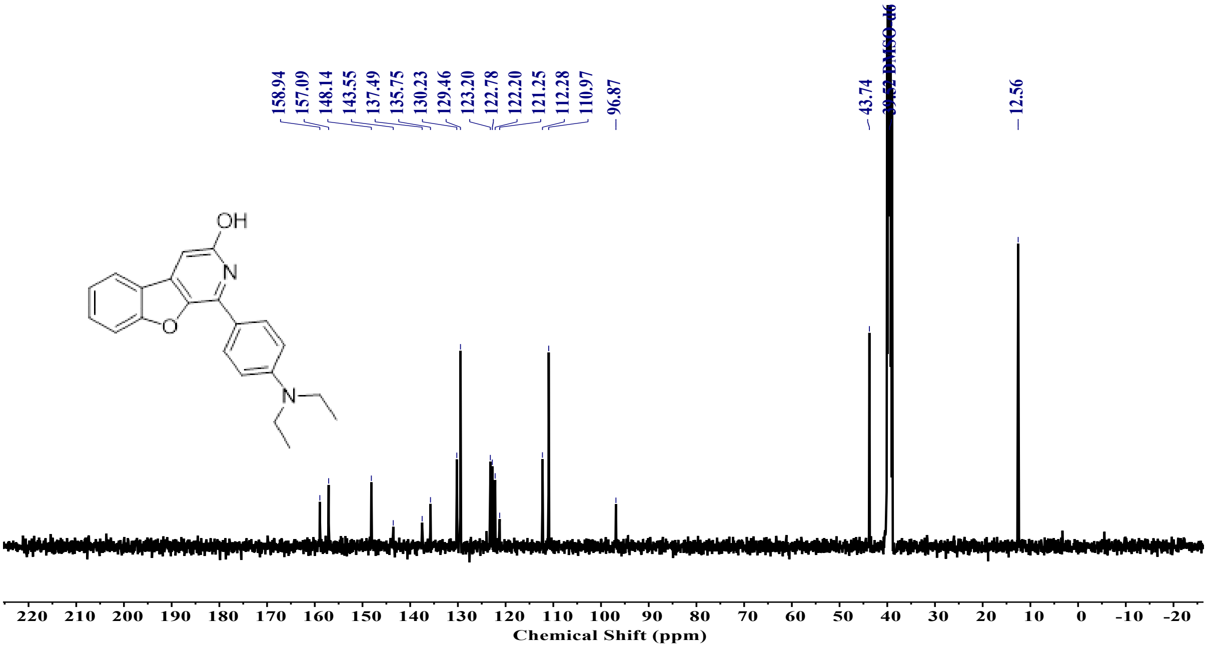

Figure18. ^{13}C NMR (125 MHz, DMSO-d_6) spectrum of 1-(4-(diethylamino)phenyl)benzofuro[2,3-c]pyridin-3-ol (**6i**)

10. 1-(4-(pyrrolidin-1-yl)phenyl)benzofuro[2,3-c]pyridin-3-ol (6*J*)

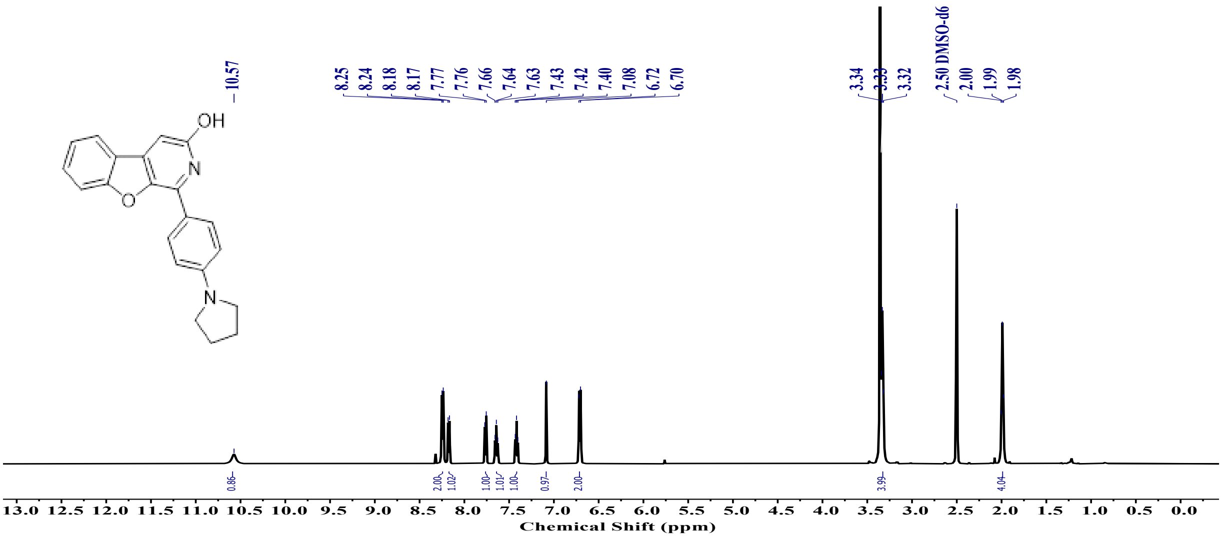

Figure 19. ^{1}H NMR (500 MHz, DMSO-d_6) spectrum of 1-(4-(pyrrolidin-1-yl)phenyl)benzofuro[2,3-c]pyridin-3-ol (***6J***)

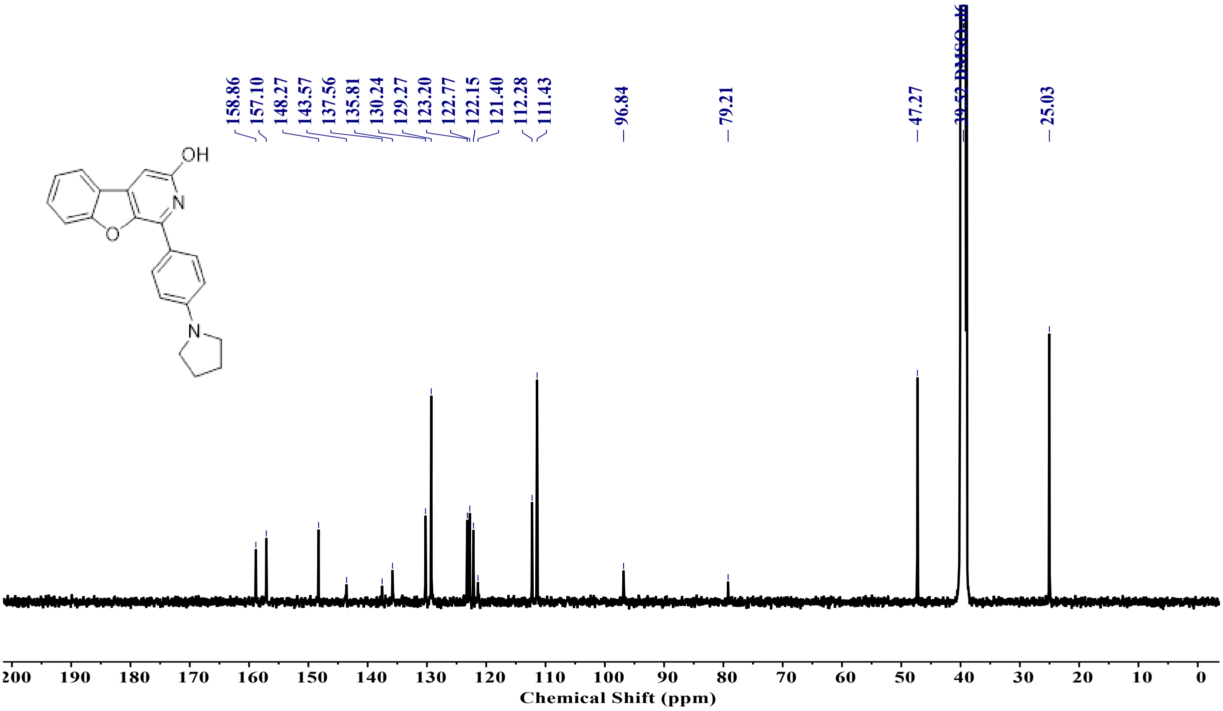

Figure 20. ^{13}C NMR (125 MHz, DMSO-d_6) spectrum of 1-(4-(pyrrolidin-1-yl) phenyl)benzofuro[2,3-c]pyridin-3-ol (***6J***)

11. 1-(4-morpholinophenyl)benzofuro[2,3-c]pyridin-3-ol (6l)

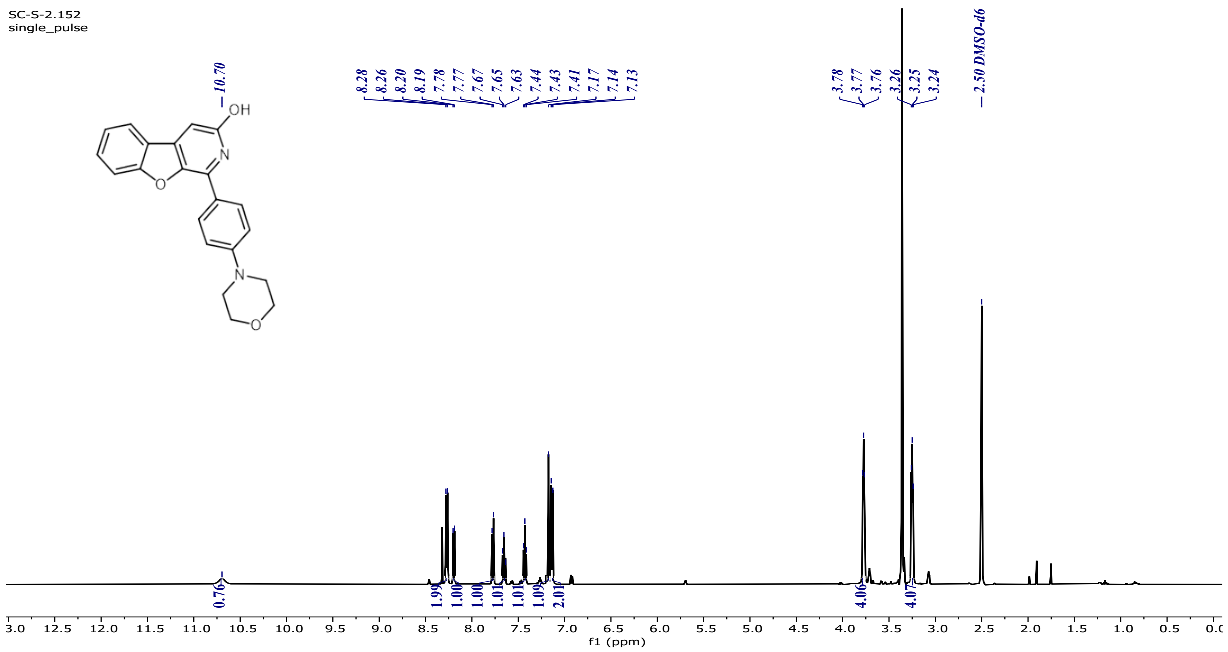

Figure 21. ^{1}H NMR (500 MHz, DMSO-d_6) spectrum of 1-(4-morpholinophenyl)benzofuro[2,3-c]pyridin-3-ol (**6l**).

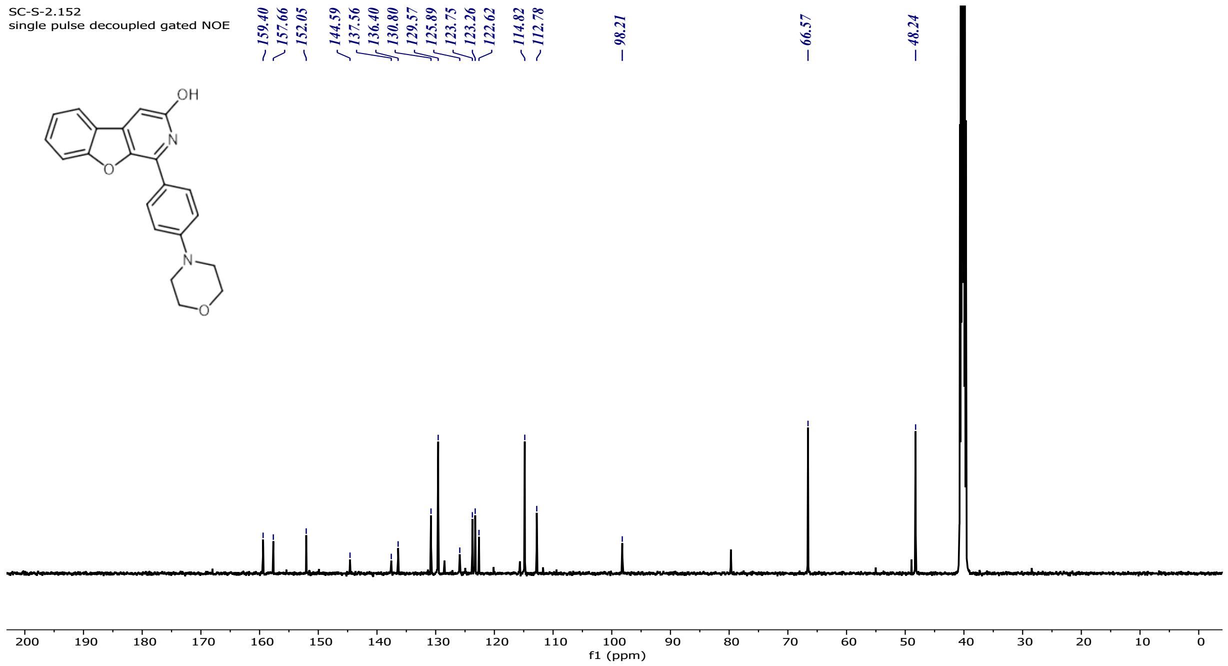

Figure 22. ^{13}C NMR (125 MHz, DMSO-d_6) spectrum of 1-(4-morpholinophenyl)benzofuro[2,3-c]pyridin-3-ol (**6l**).

12. 1-(4-thiomorpholinophenyl)benzofuro[2,3-c]pyridin-3-ol (6m)

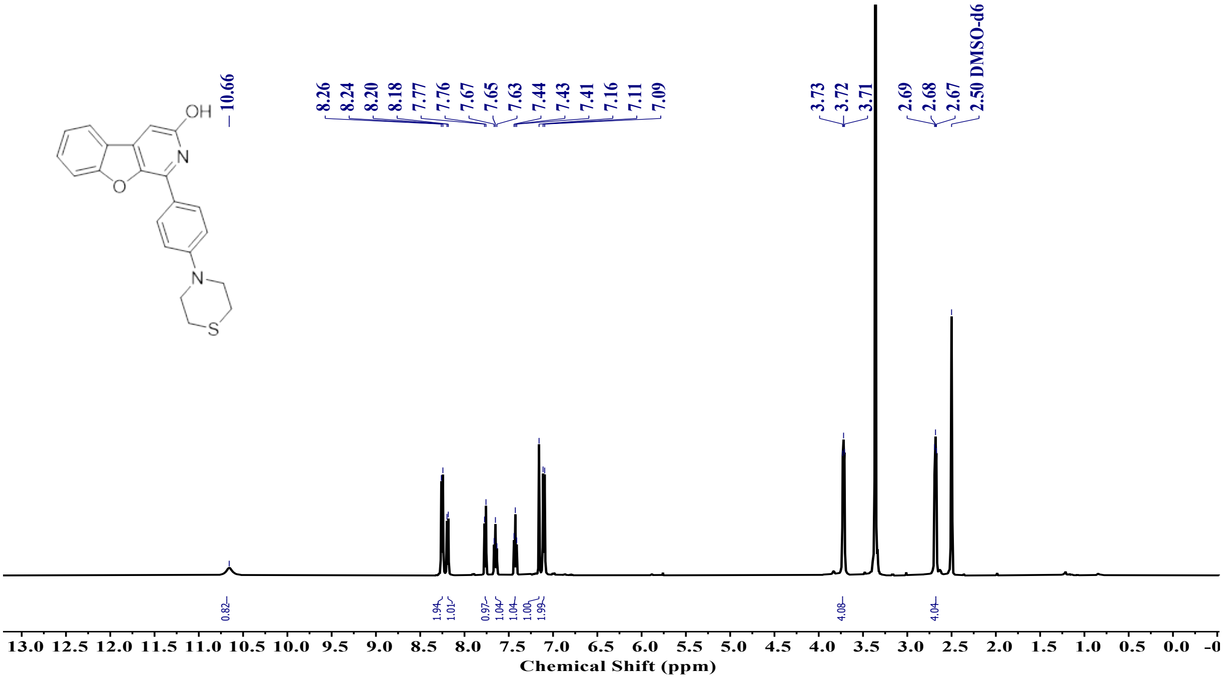

Figure 23. ^{1}H NMR (500 MHz, DMSO-d_6) spectrum of 1-(4-thiomorpholinophenyl)benzofuro[2,3-c]pyridin-3-ol (**6m**)

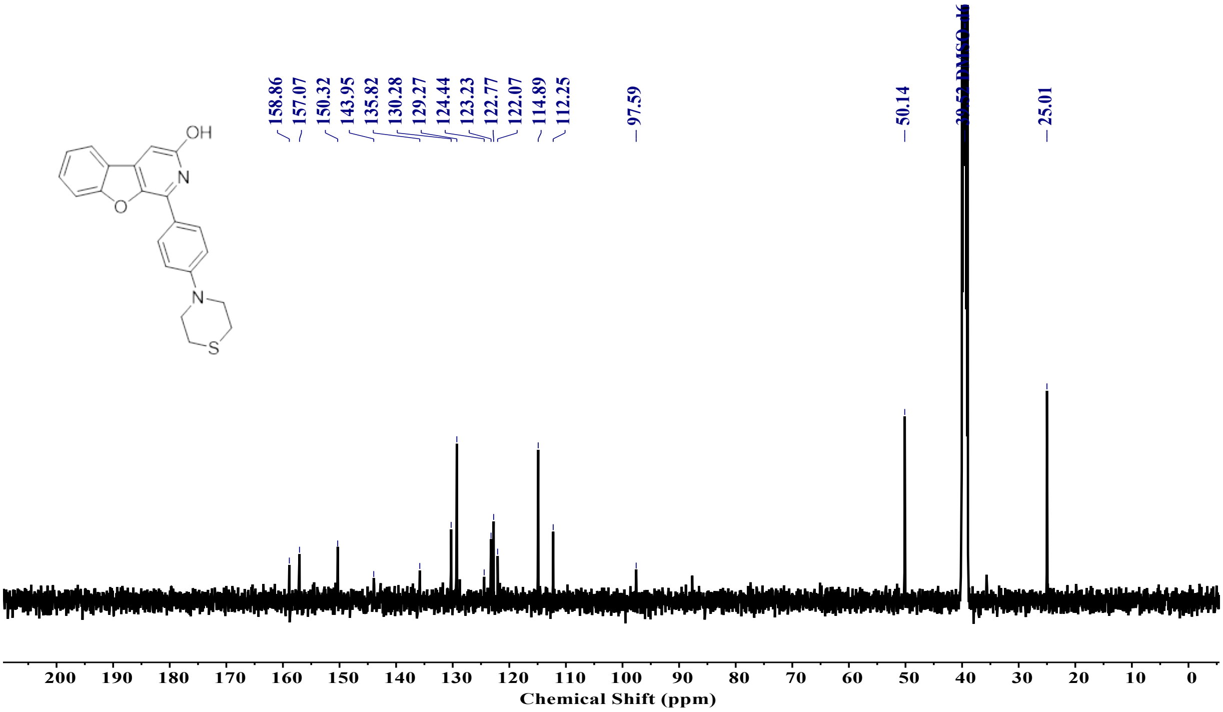

Figure 24. ^{13}C NMR (125 MHz, DMSO-d_6) spectrum of 1-(4-thiomorpholinophenyl)benzofuro[2,3-c]pyridin-3-ol (**6m**)

13. 6-methoxy-1-phenylbenzofuro[2,3-c]pyridin-3-ol (7a)

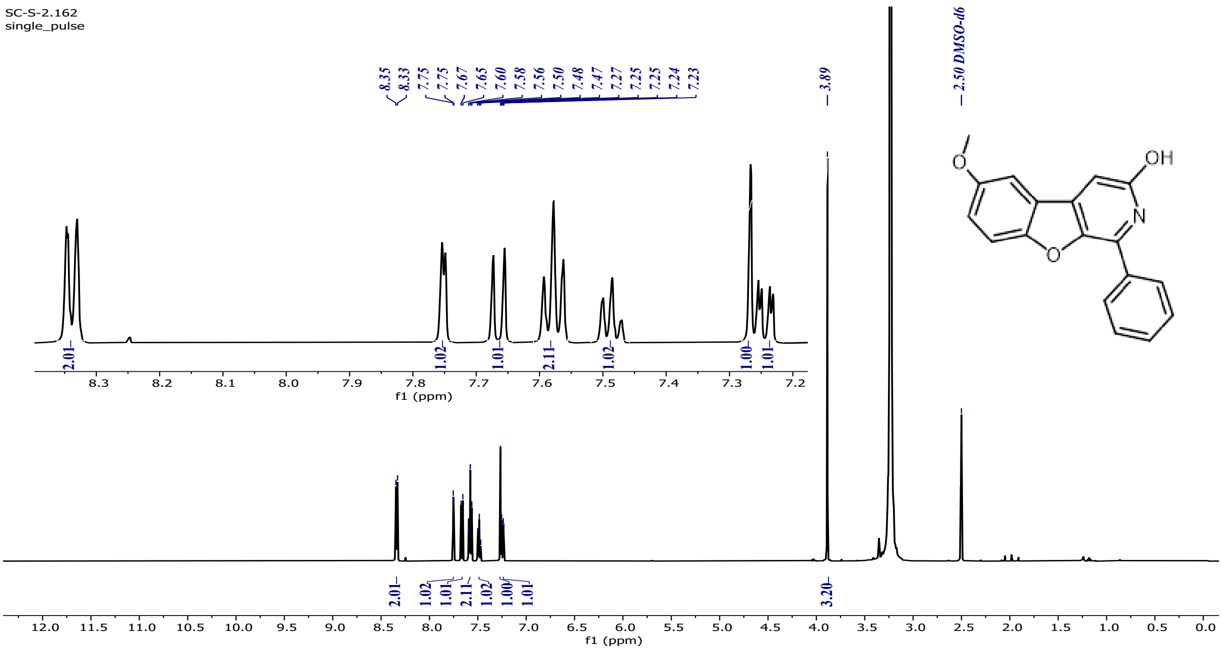

Figure 25. ^{1}H NMR (500 MHz, DMSO-d_6) spectrum of 6-methoxy-1-phenylbenzofuro[2,3-c]pyridin-3-ol (**7a**).

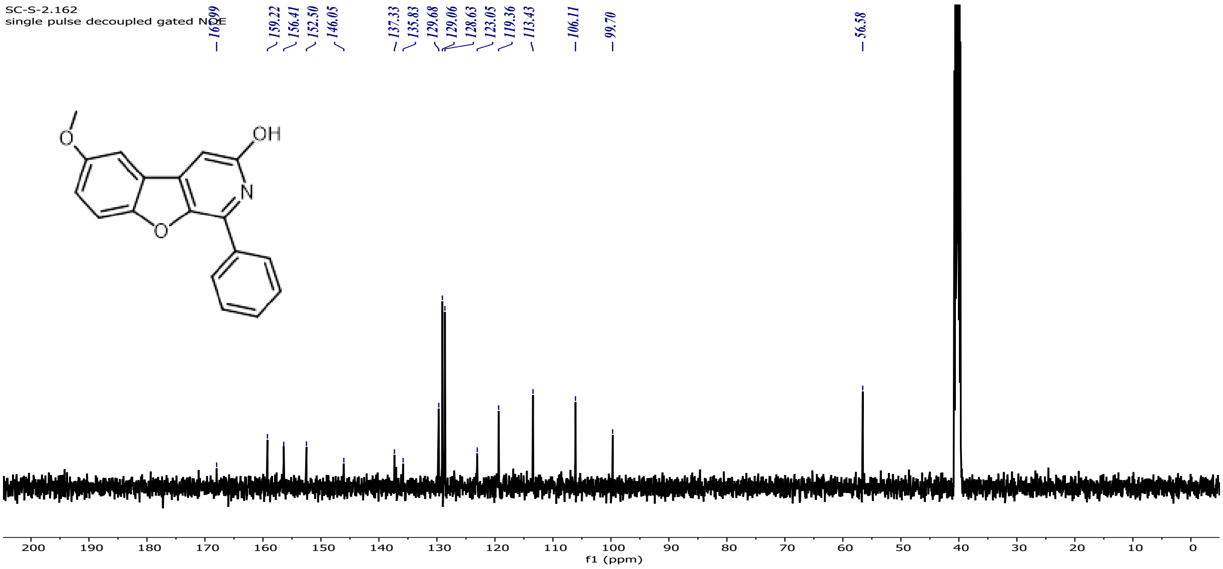

Figure 26. ^{13}C NMR (125 MHz, DMSO-d_6) spectrum of 6-methoxy-1-phenylbenzofuro[2,3-c]pyridin-3-ol (**7a**).

14. 1-(4-fluorophenyl)-6-methoxybenzofuro[2,3-c]pyridin-3-ol (7b)

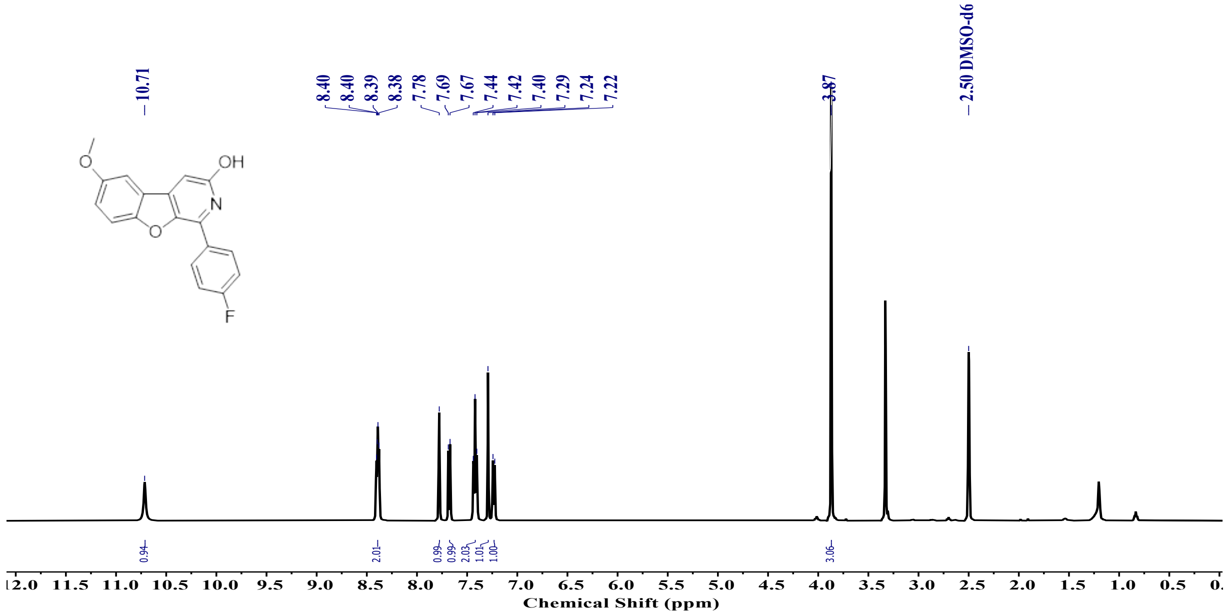

Figure 27. ^{1}H NMR (500 MHz, DMSO-d_6) spectrum of 1-(4-fluorophenyl)-6-methoxybenzofuro[2,3-c]pyridin-3-ol (**7b**).

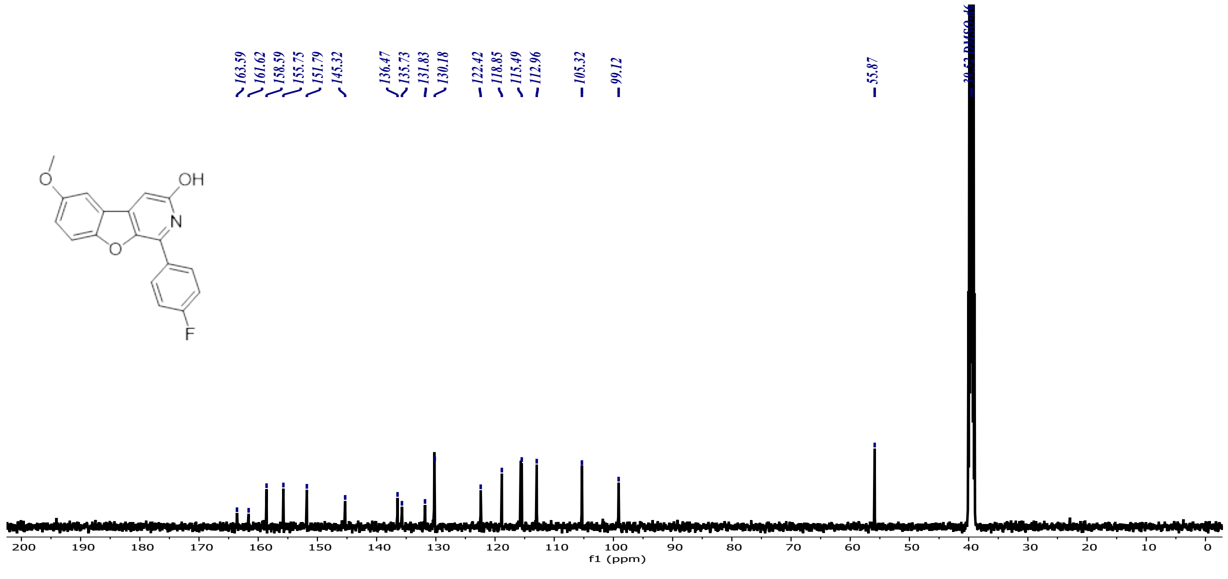

Figure 28. ^{13}C NMR (125 MHz, DMSO-d_6) spectrum of 1-(4-fluorophenyl)-6-methoxybenzofuro[2,3-c]pyridin-3-ol (**7b**).

15. 1-(4-chlorophenyl)-6-methoxybenzofuro[2,3-c]pyridin-3-ol (7c)

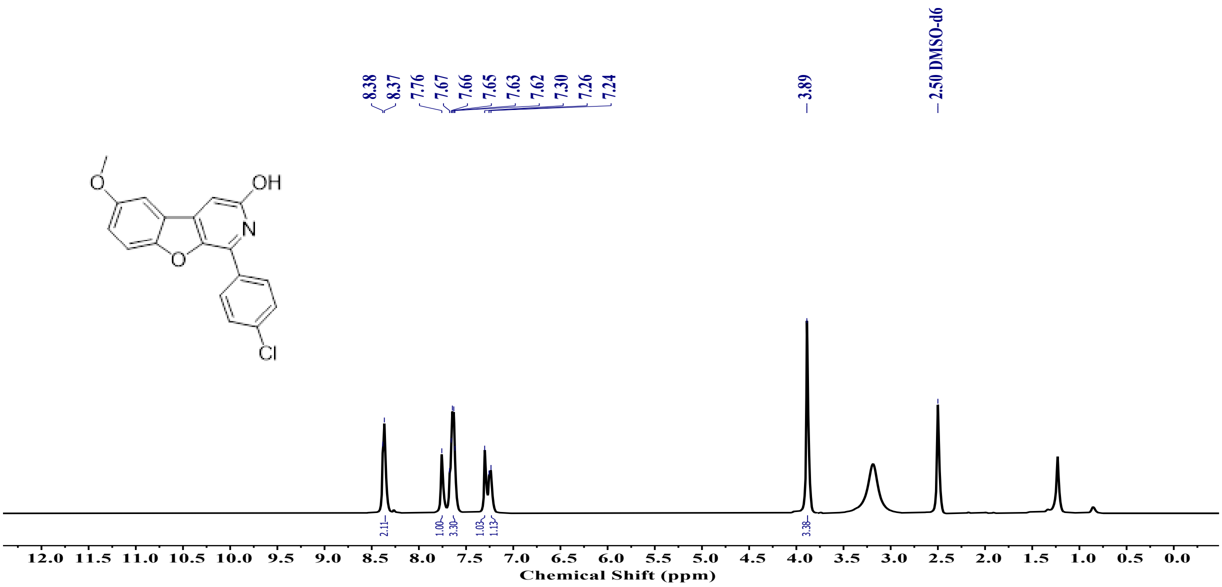

Figure 29. ^{1}H NMR (500 MHz, DMSO-d_6) spectrum of 1-(4-chlorophenyl)-6-methoxybenzofuro[2,3-c]pyridin-3-ol (**7c**)

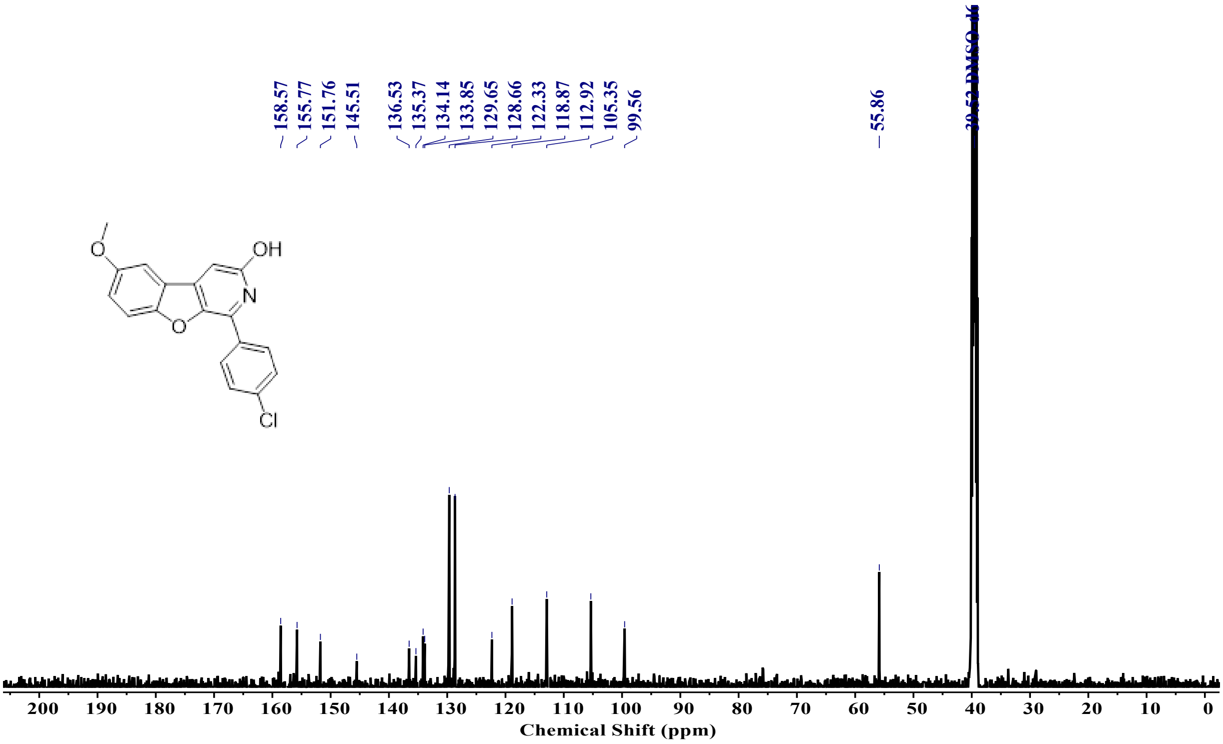

Figure 30. ^{13}C NMR (125 MHz, DMSO-d_6) spectrum of 1-(4-chlorophenyl)-6-methoxybenzofuro[2,3-c]pyridin-3-ol (**7c**)

16. 6-methoxy-1-(4-methoxyphenyl)benzofuro[2,3-c]pyridin-3-ol (7d)

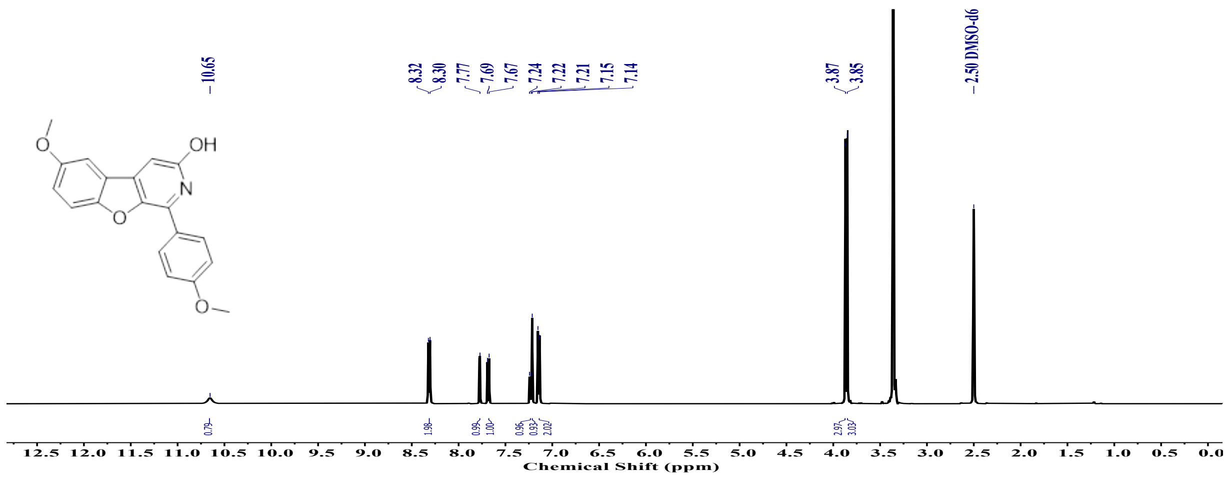

Figure 31. ^{1}H NMR (500 MHz, DMSO-d_6) spectrum of 6-methoxy-1-(4-methoxyphenyl)benzofuro[2,3-c]pyridin-3-ol(**7d**).

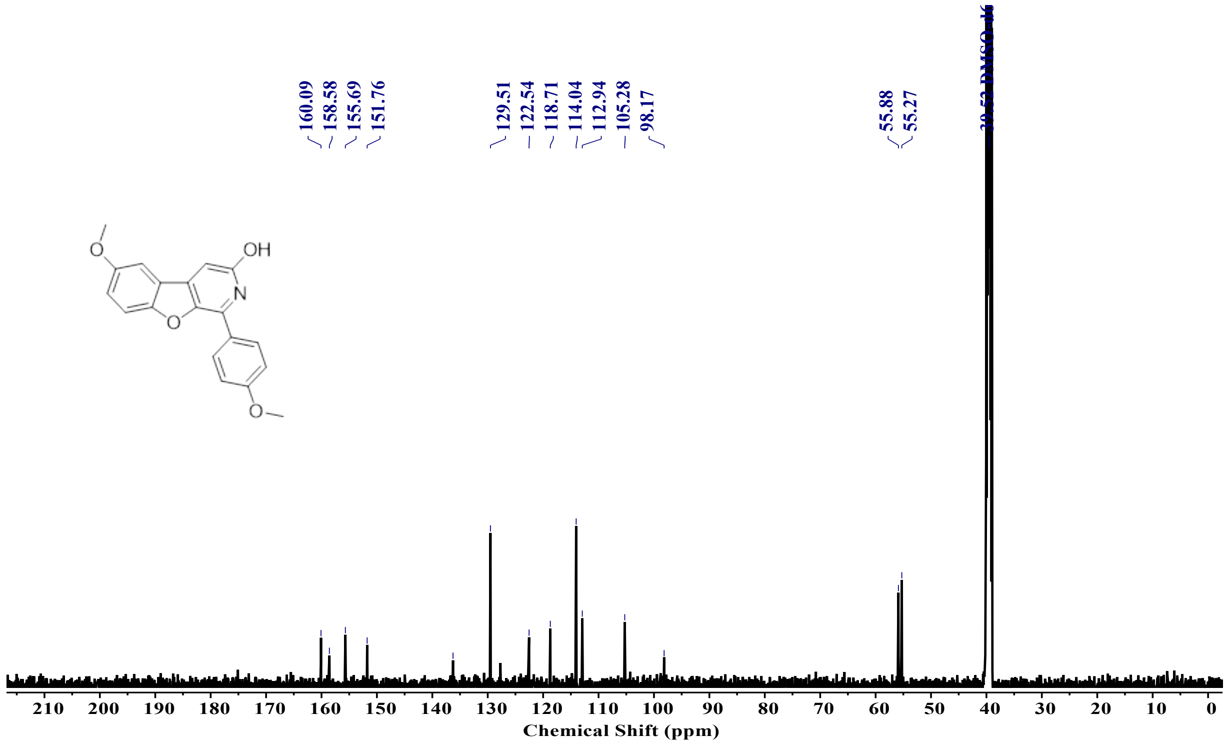

Figure 32. ^{13}C NMR (125 MHz, DMSO-d_6) spectrum of 6-methoxy-1-(4-methoxyphenyl)benzofuro[2,3-c]pyridin-3-ol (**7d**) .

17. 1-([1,1'-biphenyl]-4-yl)-6-methoxybenzofuro[2,3-c]pyridin-3-ol (7e)

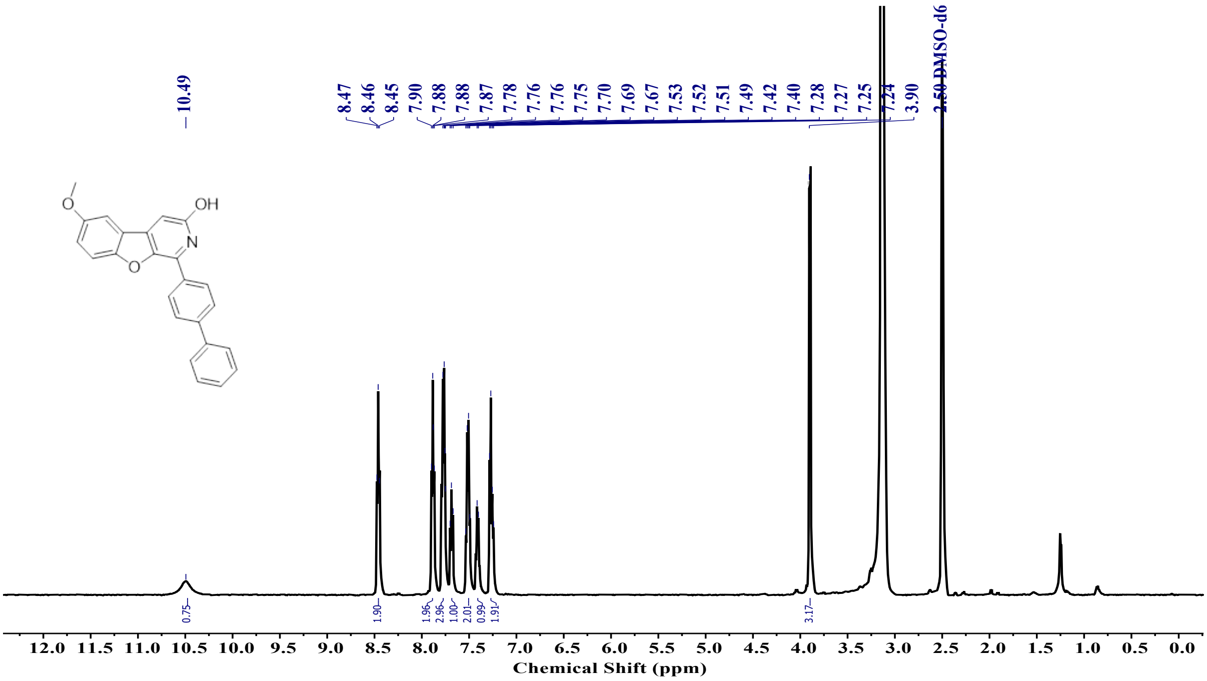

Figure 33. ^{1}H NMR(500 MHz, DMSO-d_6) spectrum of 1-([1,1'-biphenyl]-4-yl)-6-methoxybenzofuro[2,3-c]pyridin-3-ol (**7e**)

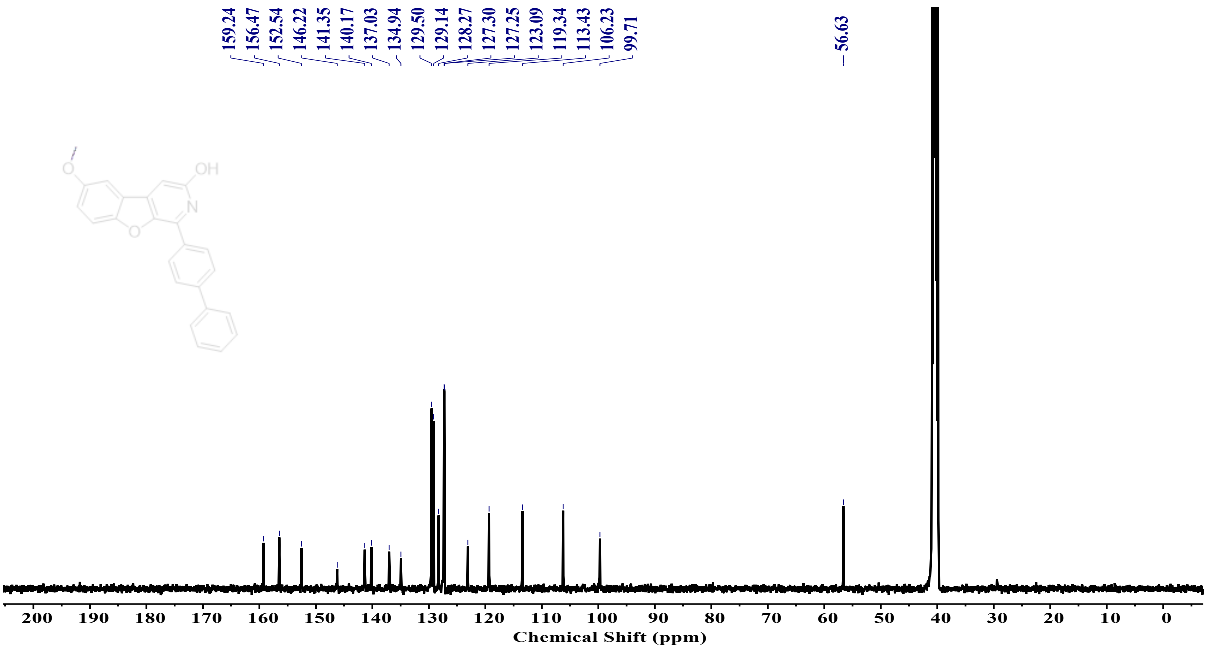

Figure 34. ^{13}C NMR (125 MHz, DMSO-d_6) spectrum of 1-([1,1'-biphenyl]-4-yl)-6-methoxybenzofuro[2,3-c]pyridin-3-ol (**7e**)

18. 6-methoxy-1-(naphthalen-2-yl)benzofuro[2,3-c]pyridin-3-ol (7f)

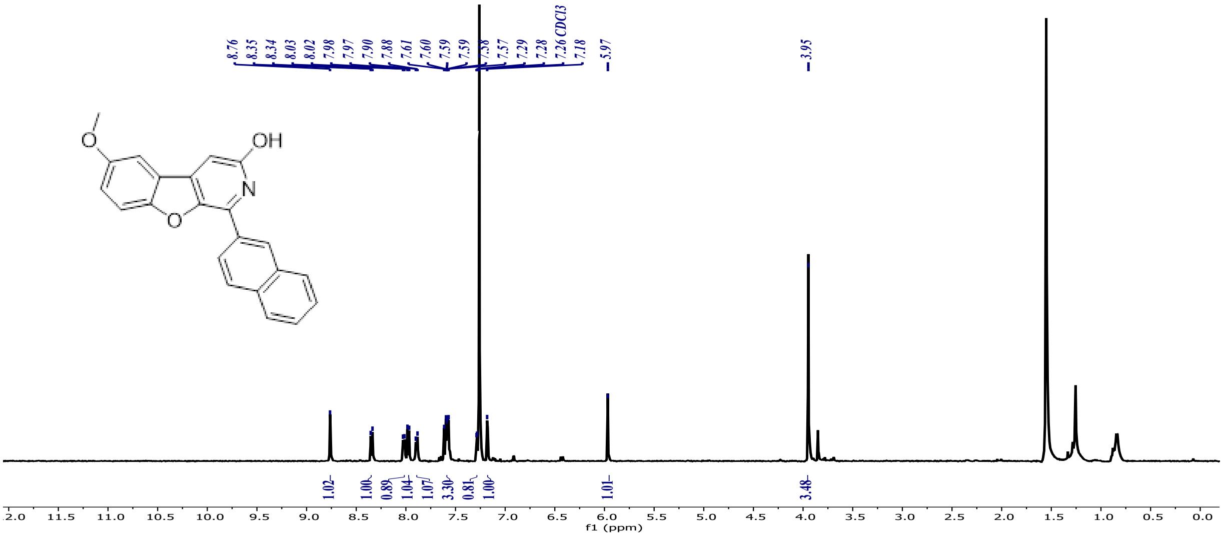

Figure 35. ^{1}H NMR (500 MHz, $CDCl_3$) spectrum of 6-methoxy-1-(naphthalen-2-yl)benzofuro[2,3-c]pyridin-3-ol (**7f**)

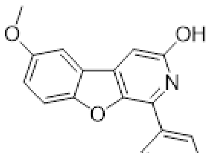

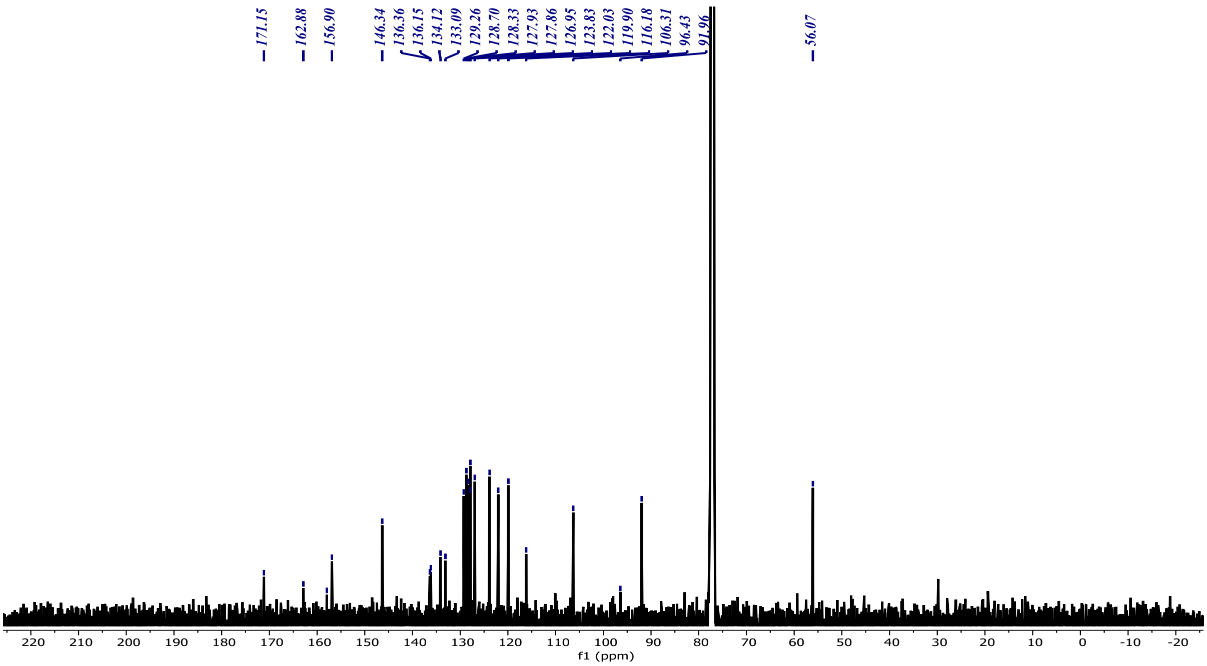

Figure 36. ^{13}C NMR (125 MHz, $CDCl_3$)spectrum of 6-methoxy-1-(naphthalen-2-yl)benzofuro[2,3-c]pyridin-3-ol (**7f**)

19. 1-(4-(diethylamino)phenyl)-6-methoxybenzofuro[2,3-c]pyridin-3-ol (7g)

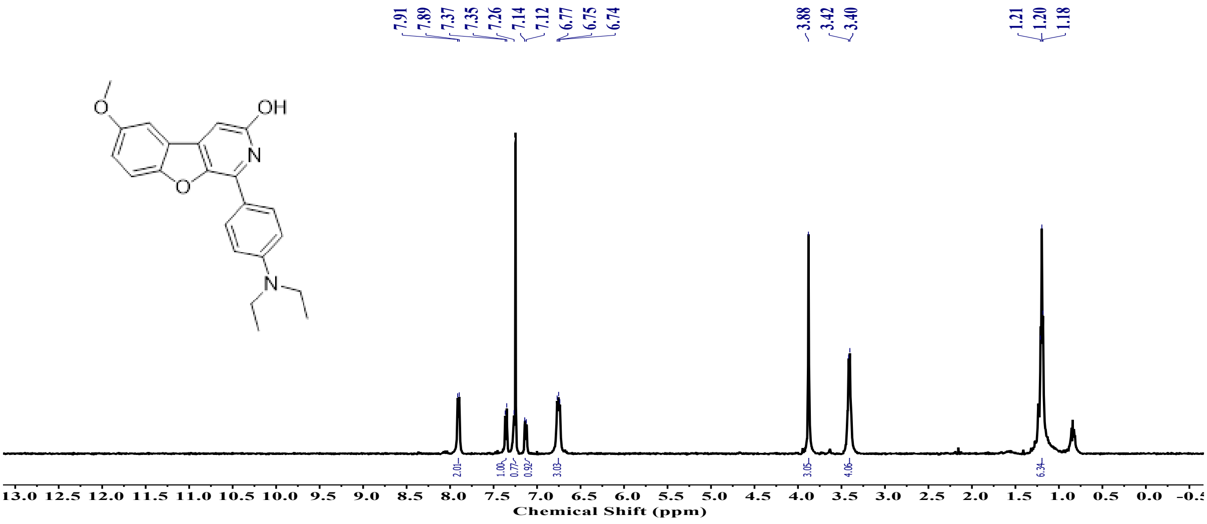

Figure 37. ^{1}H NMR (500 MHz, $CDCl_3$) spectrum of 1-(4-(diethylamino)phenyl)-6-methoxybenzofuro[2,3-c]pyridin-3-ol (**7g**)

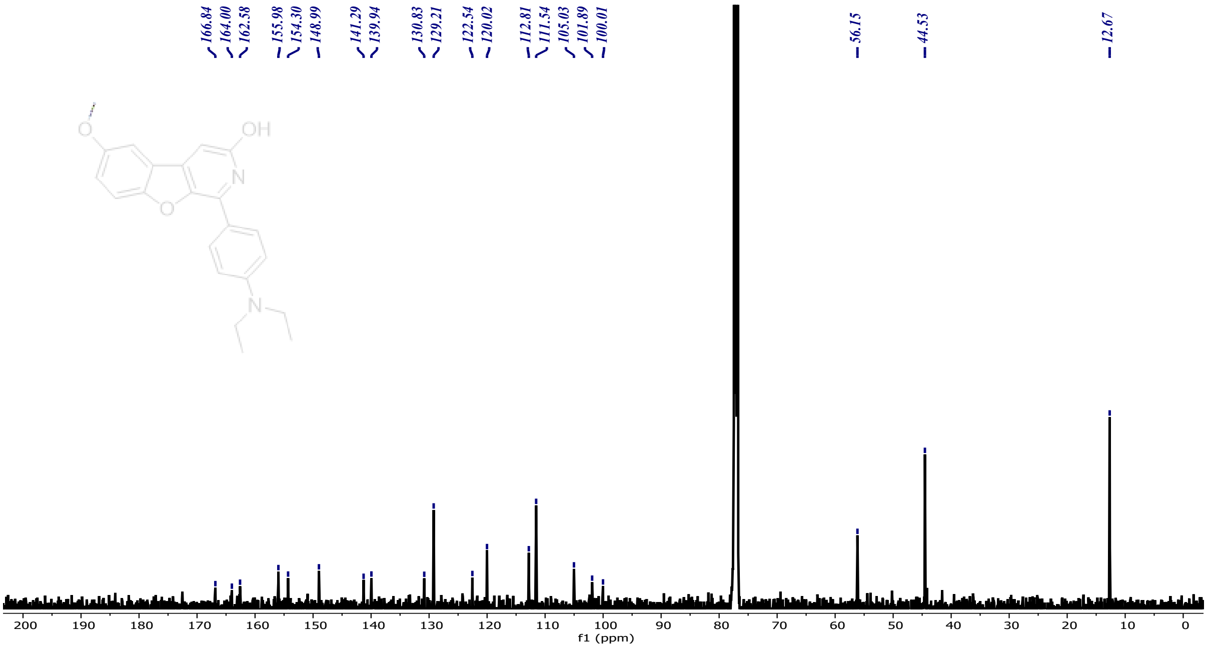

Figure 38. ^{13}C NMR (125 MHz, $CDCl_3$)spectrum of 1-(4-(diethylamino)phenyl)-6-methoxybenzofuro[2,3-c]pyridin-3-ol (**7g**)

20. 6-methoxy-1-(4-(pyrrolidin-1-yl)phenyl)benzofuro[2,3-c]pyridin-3-ol (7h)

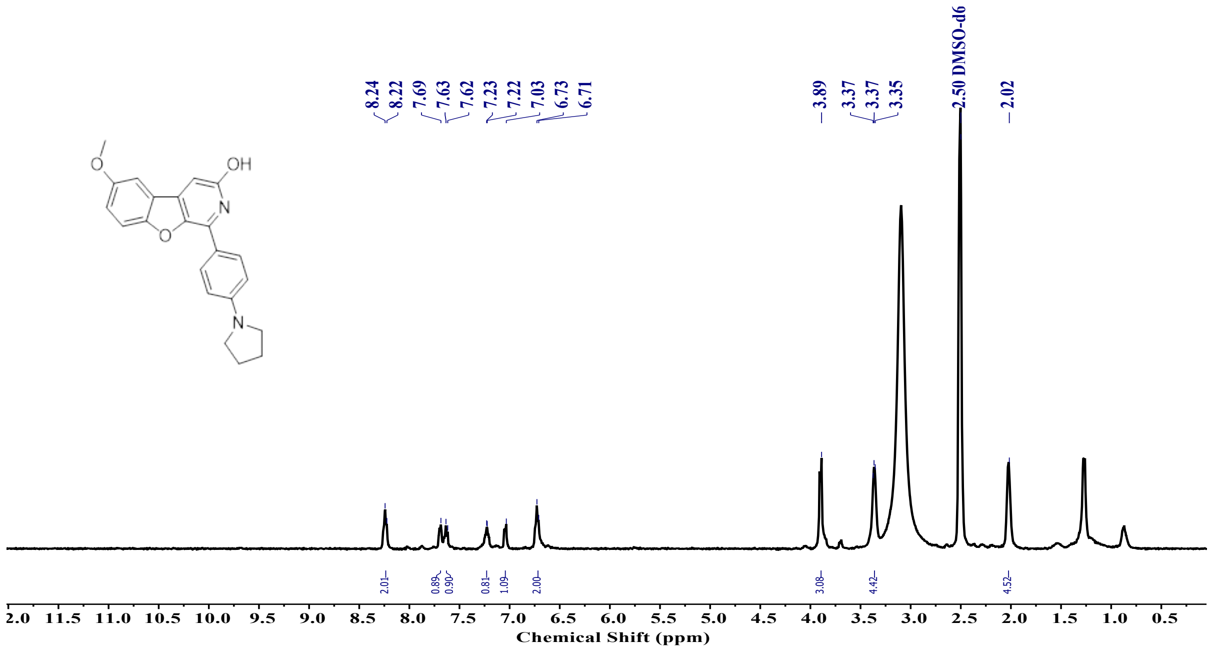

Figure 39. ^{1}H NMR (500 MHz, DMSO-d_6) spectrum of 6-methoxy-1-(4-(pyrrolidin-1-yl)phenyl)benzofuro[2,3-c]pyridin-3-ol (**7h**)

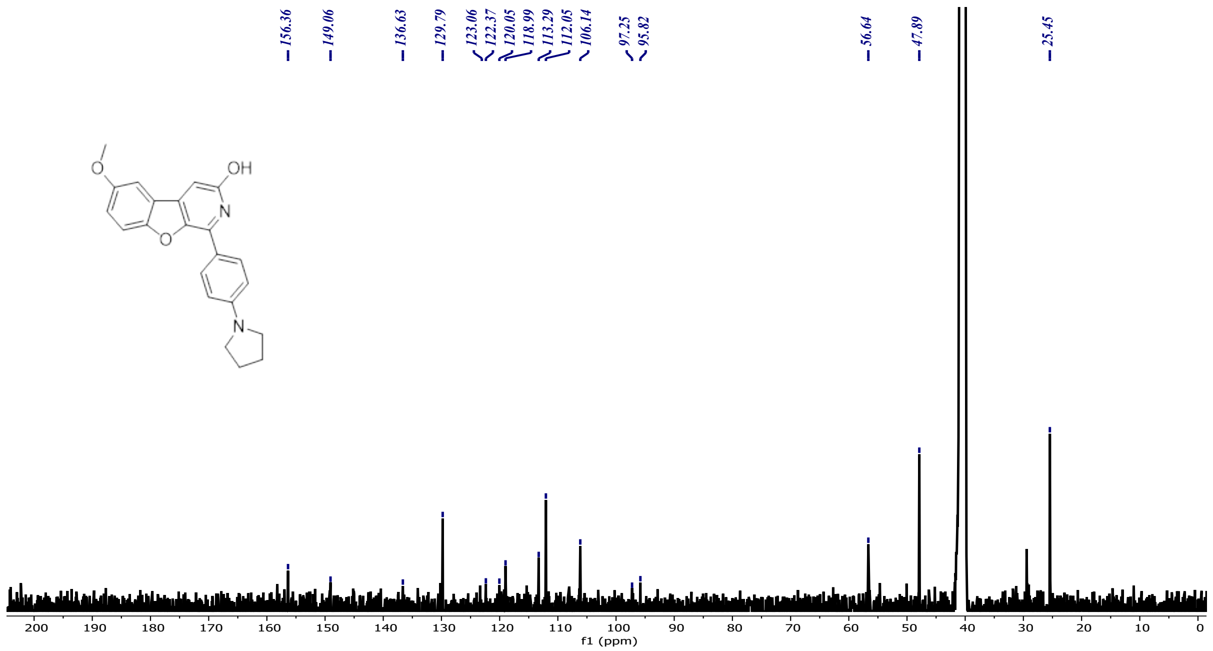

Figure 40. ^{13}C NMR (125 MHz, DMSO-d_6) spectrum of 6-methoxy-1-(4-(pyrrolidin-1-yl)phenyl)benzofuro[2,3-c]pyridin-3-ol (**7h**)

21. 6-methoxy-1-(4-(piperidin-1-yl)phenyl)benzofuro[2,3-c]pyridin-3-ol (7i)

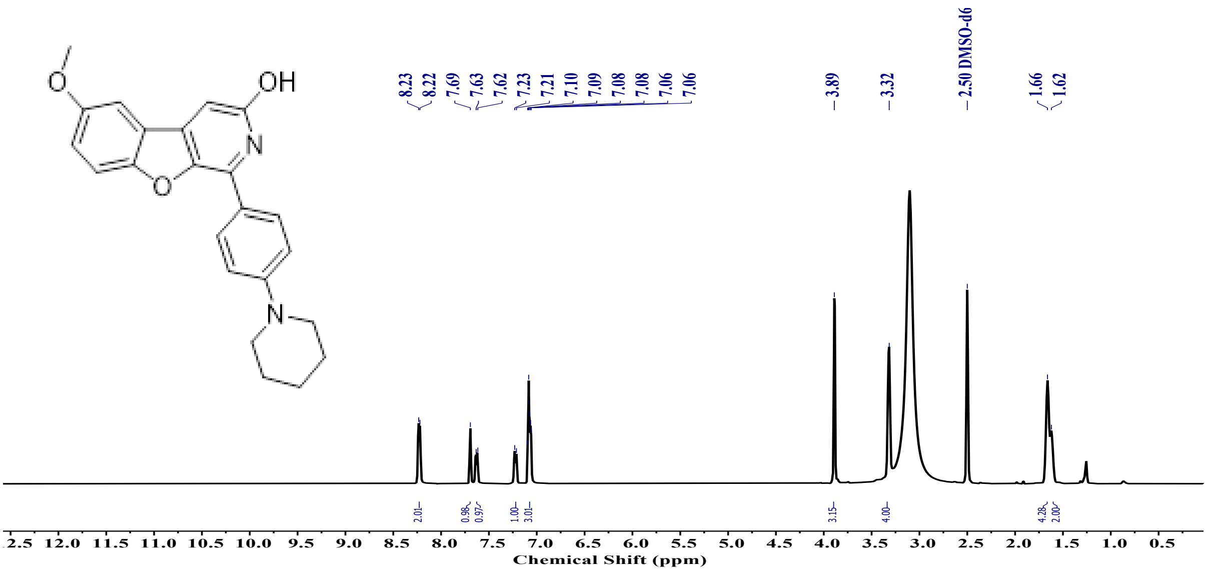

Figure 41. ^{1}H NMR (500 MHz, DMSO-d_6) spectrum of 6-methoxy-1-(4-(piperidin-1-yl)phenyl)benzofuro[2,3-c]pyridin-3-ol (**7i**)

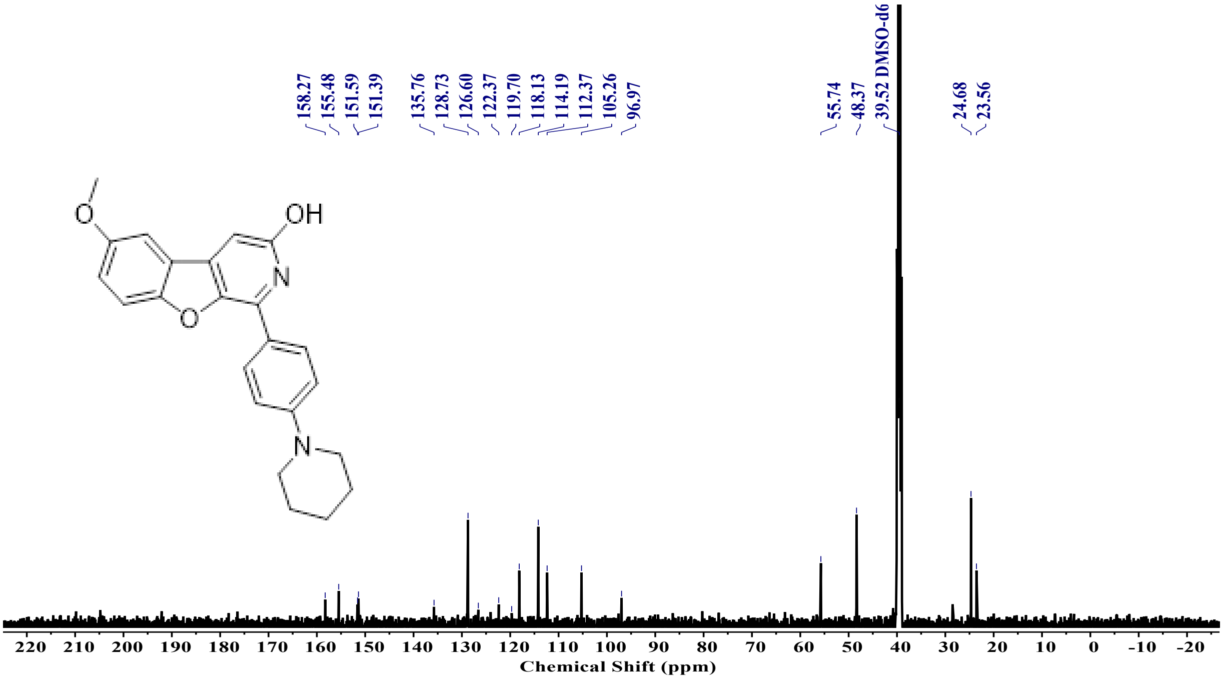

Figure 42. ^{13}C NMR (125 MHz, DMSO-d_6) spectrum of 6-methoxy-1-(4-(piperidin-1-yl)phenyl)benzofuro[2,3-c]pyridin-3-ol (**7i**)

22. 6-methoxy-1-(4-morpholinophenyl)benzofuro[2,3-c]pyridin-3-ol (7*J*)

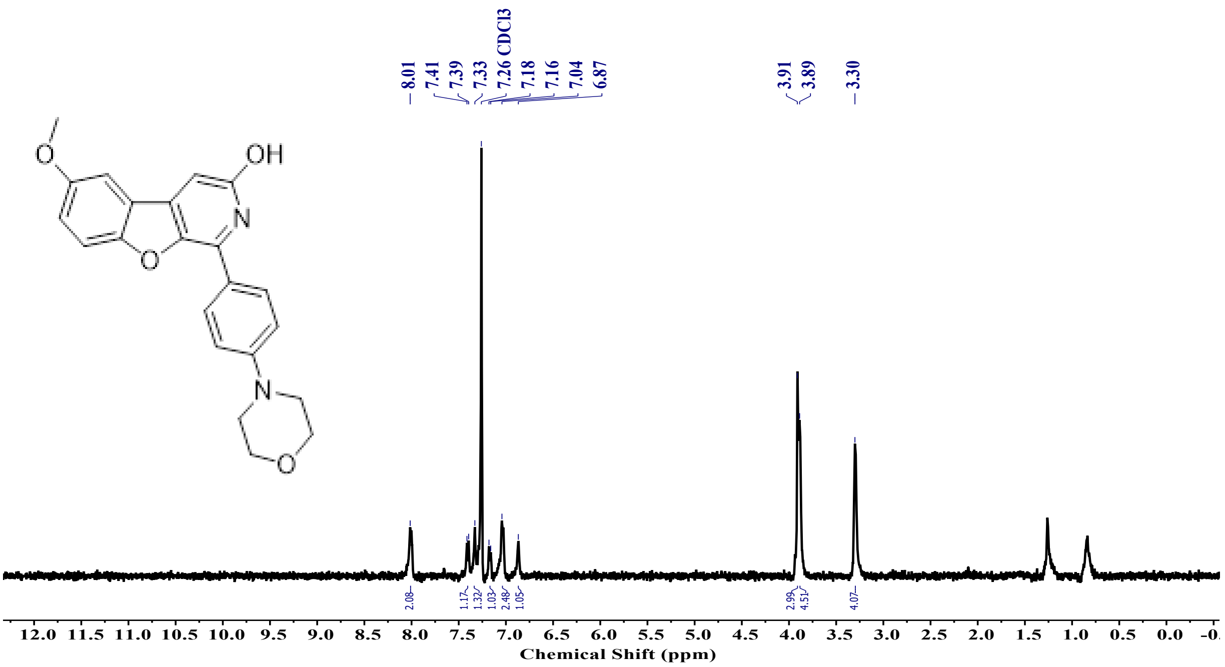

Figure 43. ^{1}H NMR (500 MHz, $CDCl_3$)spectrum of 6-methoxy-1-(4-morpholinophenyl)benzofuro[2,3-c]pyridin-3-ol (**7*J***)

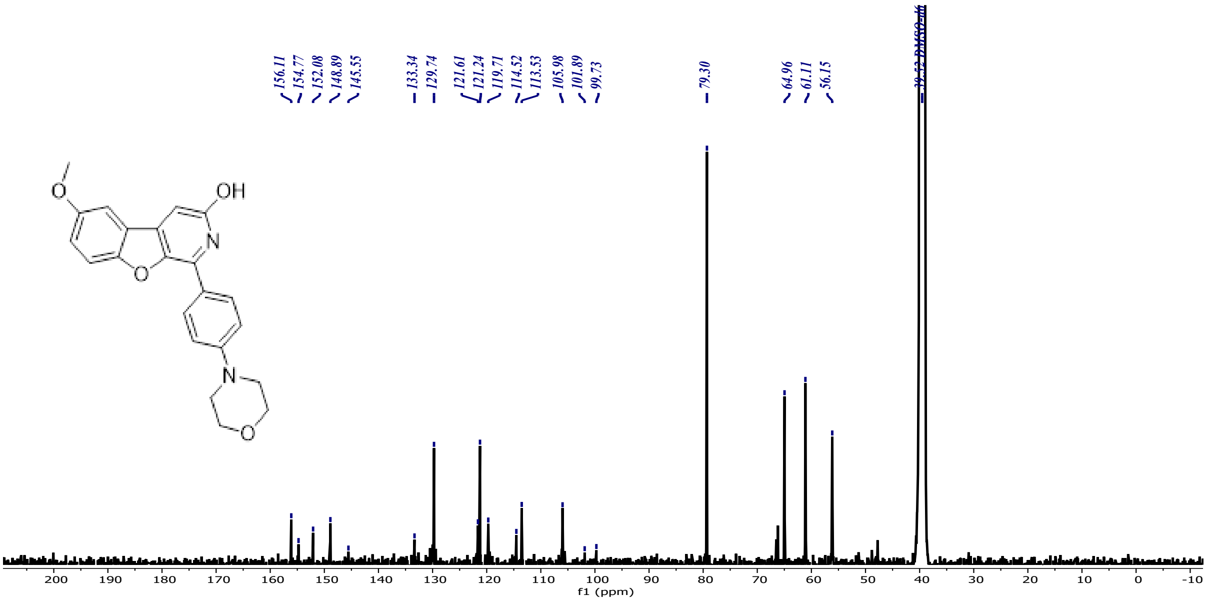

Figure 44. ^{13}C NMR (125 MHz, $CDCl_3$)spectrum of 6-methoxy-1-(4-morpholinophenyl)benzofuro[2,3-c]pyridin-3-ol (**7*J***)

23. 6-methoxy-1-(4-thiomorpholinophenyl)benzofuro[2,3-c]pyridin-3-ol (7k)

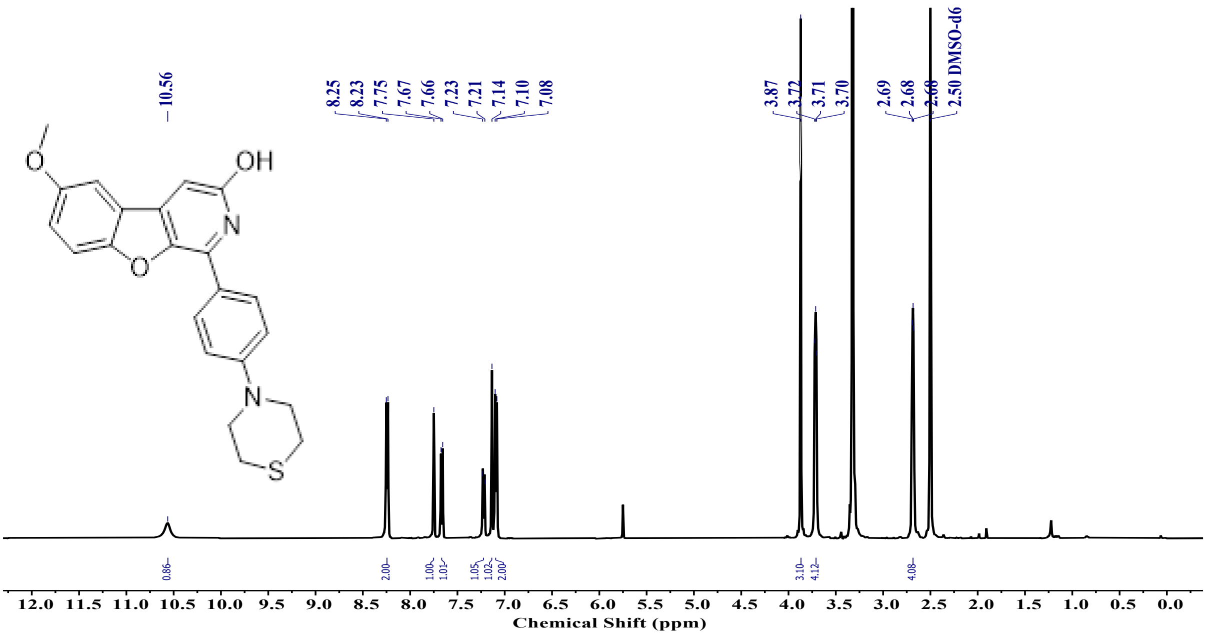

Figure 45. ^{1}H NMR (500 MHz, DMSO-d_6) spectrum of 6-methoxy-1-(4-thiomorpholinophenyl)benzofuro[2,3-c]pyridin-3-ol (**7k**)

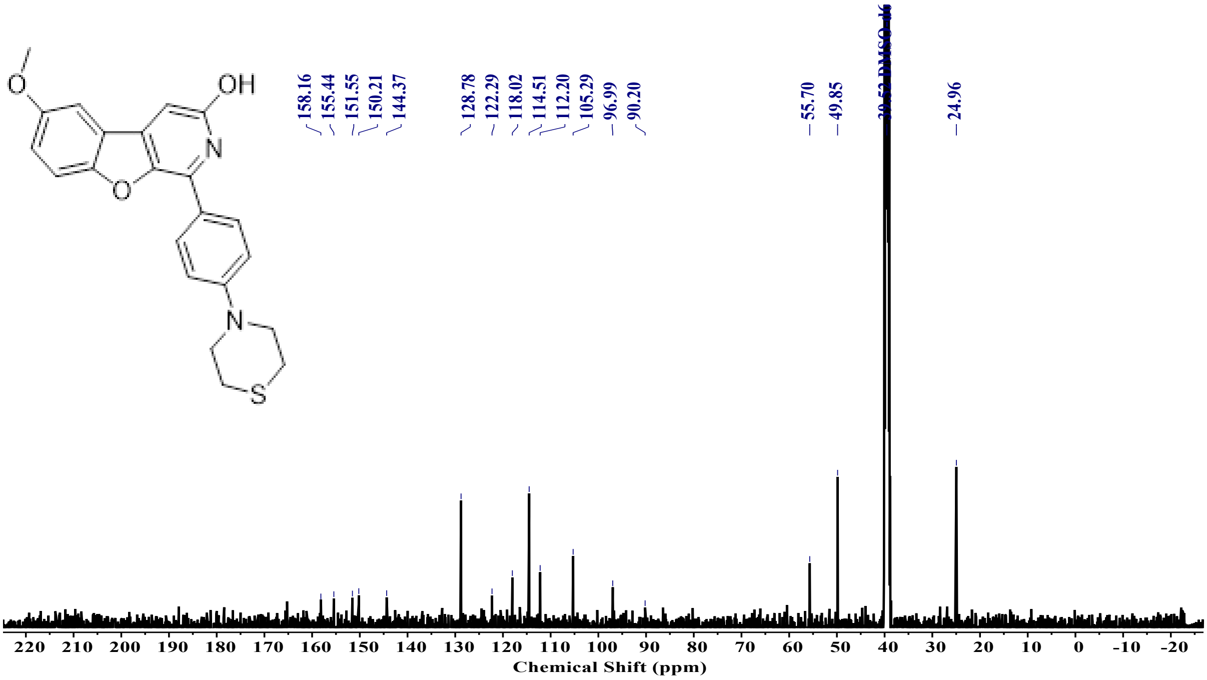

Figure 46. ^{13}C NMR (125 MHz, DMSO-d_6) spectrum of 6-methoxy-1-(4-thiomorpholinophenyl)benzofuro[2,3-c]pyridin-3-ol (**7k**)

24. 6-bromo-1-phenylbenzofuro[2,3-c]pyridin-3-ol (8a)

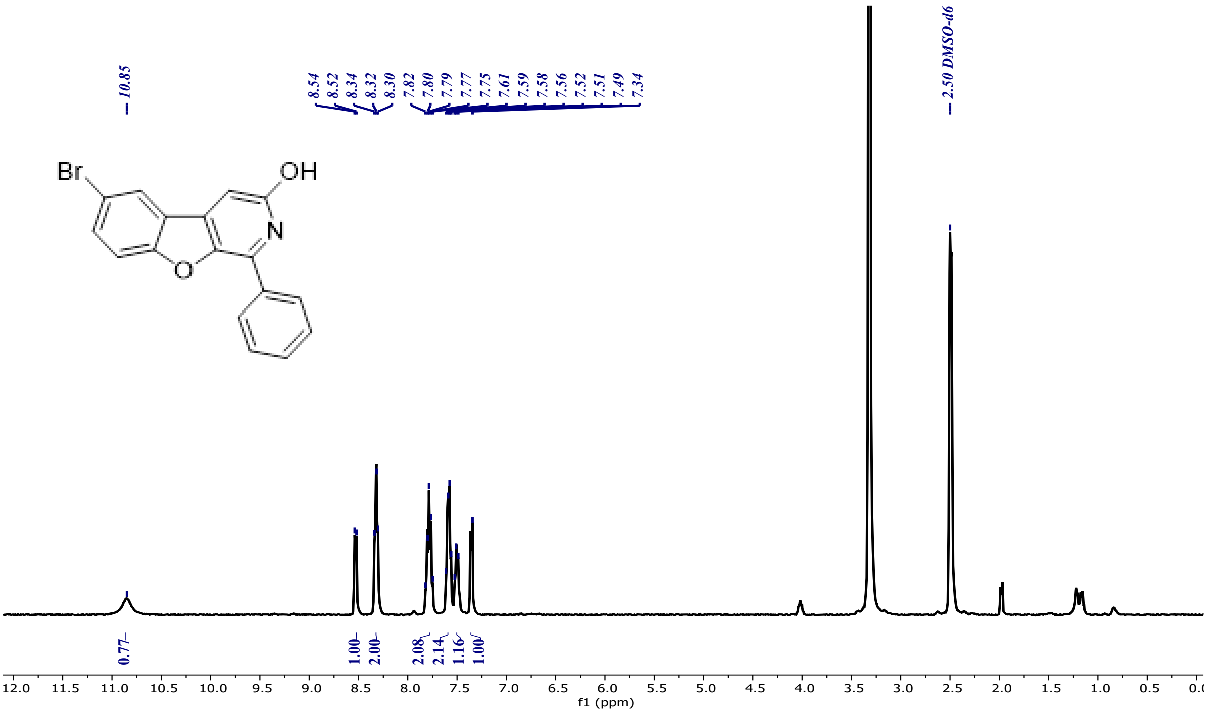

Figure 47. ^{1}H NMR (500 MHz, DMSO-d_6) spectrum of 6-bromo-1-phenylbenzofuro[2,3-c]pyridin-3-ol (**8a**).

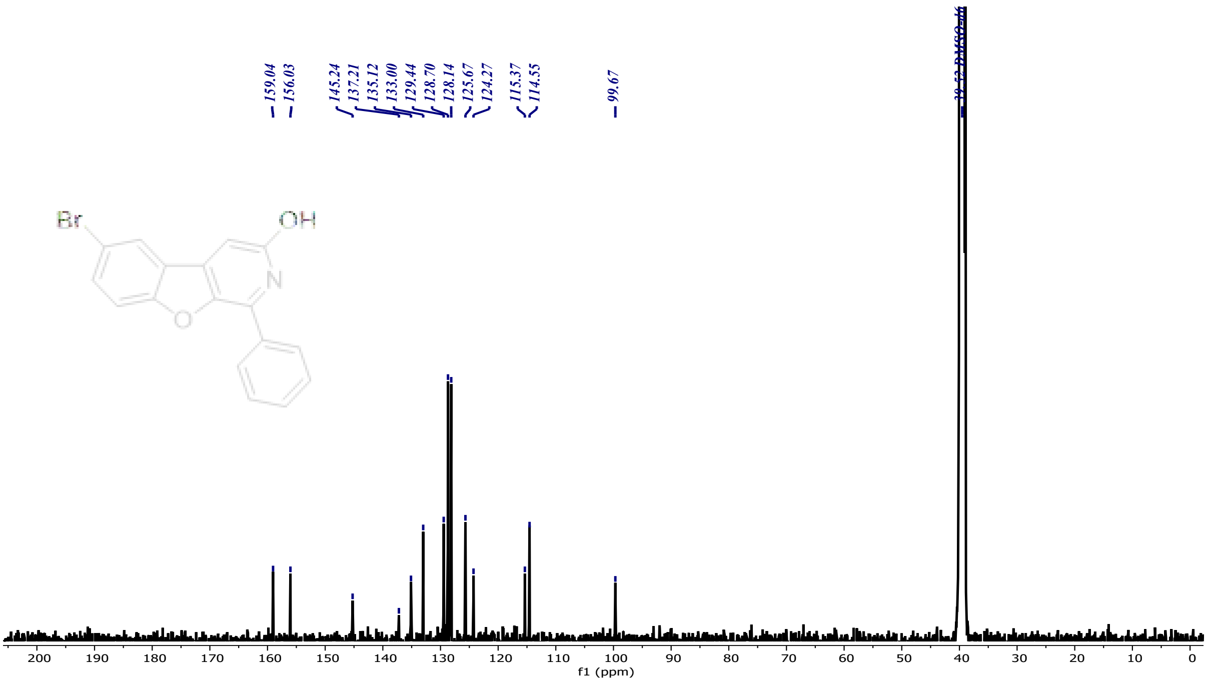

Figure 48. ^{13}C NMR (125 MHz, DMSO-d_6) spectrum of 6-bromo-1-phenylbenzofuro[2,3-c]pyridin-3-ol (**8a**).

25. 7-(diethylamino)-1-phenylbenzofuro[2,3-c]pyridin-3-ol (9a)

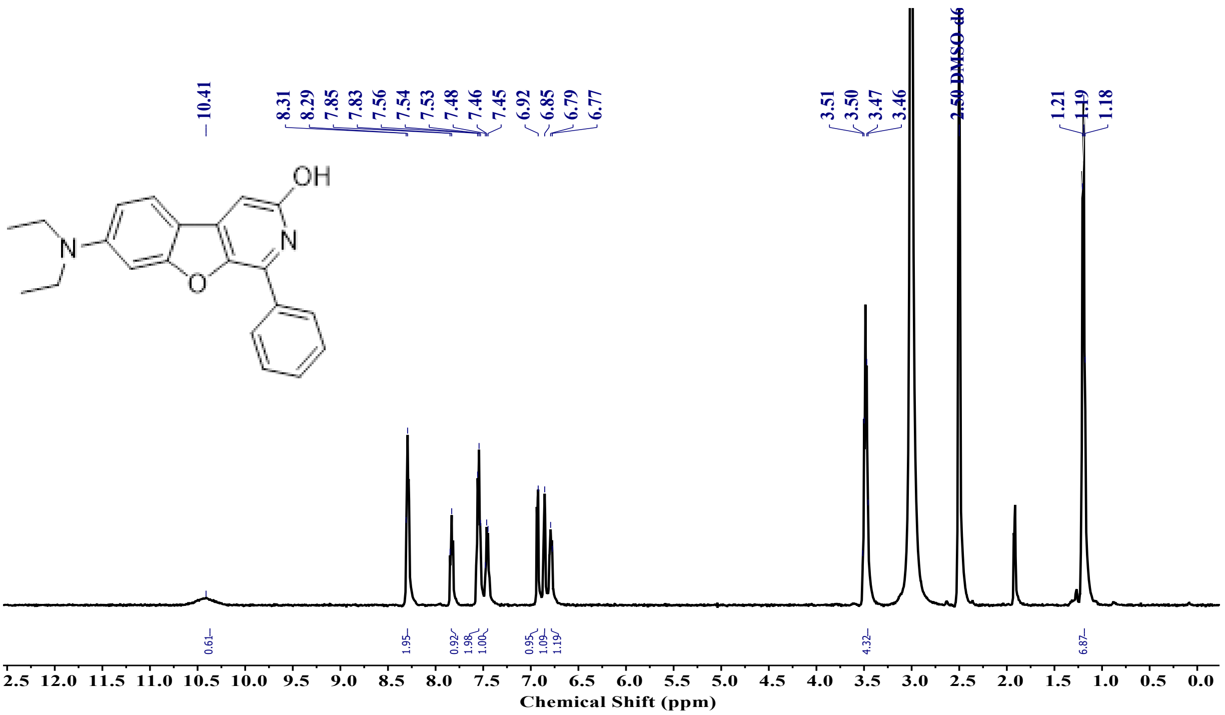

Figure 49. ^{1}H NMR (500 MHz, DMSO-d_6) spectrum of 7-(diethylamino)-1-phenylbenzofuro[2,3-c]pyridin-3-ol (**9a**)

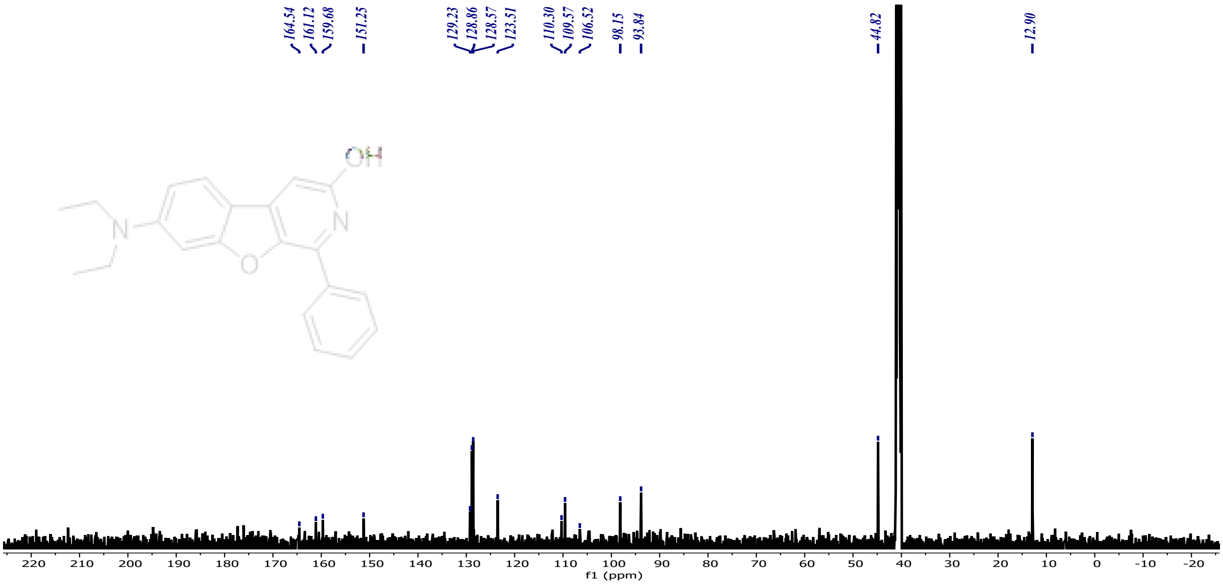

Figure 50. ^{13}C NMR (125 MHz, DMSO-d_6) spectrum of 7-(diethylamino)-1-phenylbenzofuro[2,3-c]pyridin-3-ol (**9a**)

26. 7-(diethylamino)-1-(4-fluorophenyl)benzofuro[2,3-c]pyridin-3-ol (9b)

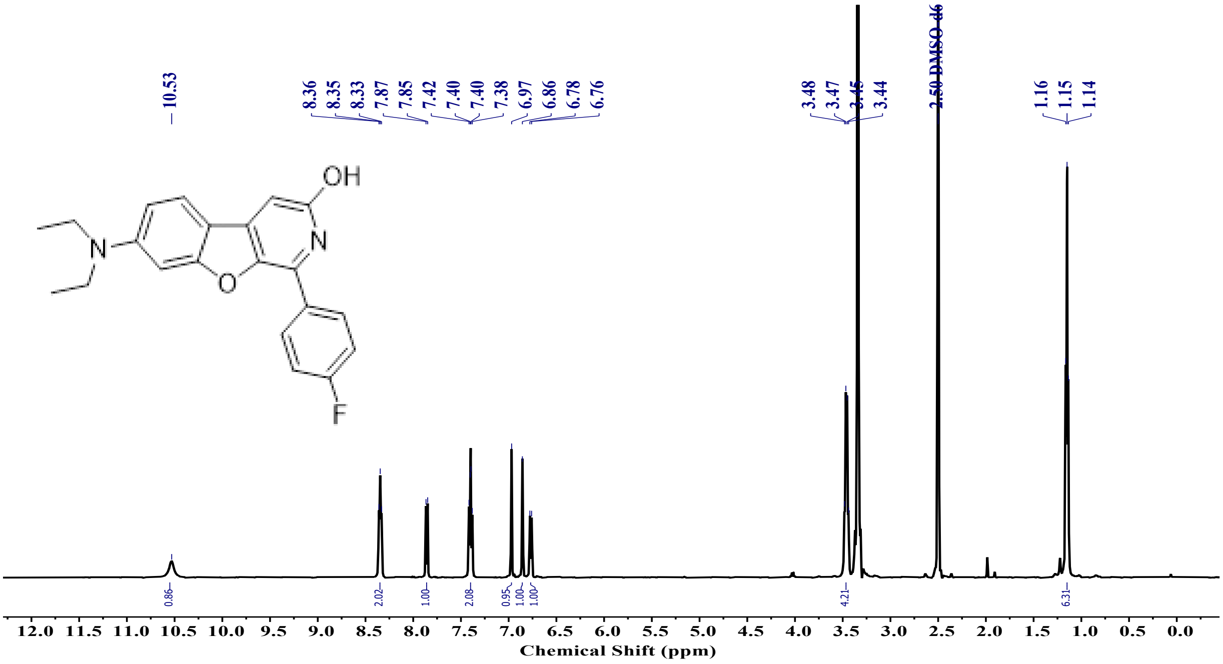

Figure 51. ^{1}H NMR (500 MHz, DMSO-d_6) spectrum of 7-(diethylamino)-1-(4-fluorophenyl)benzofuro[2,3-c]pyridin-3-ol (**9b**)

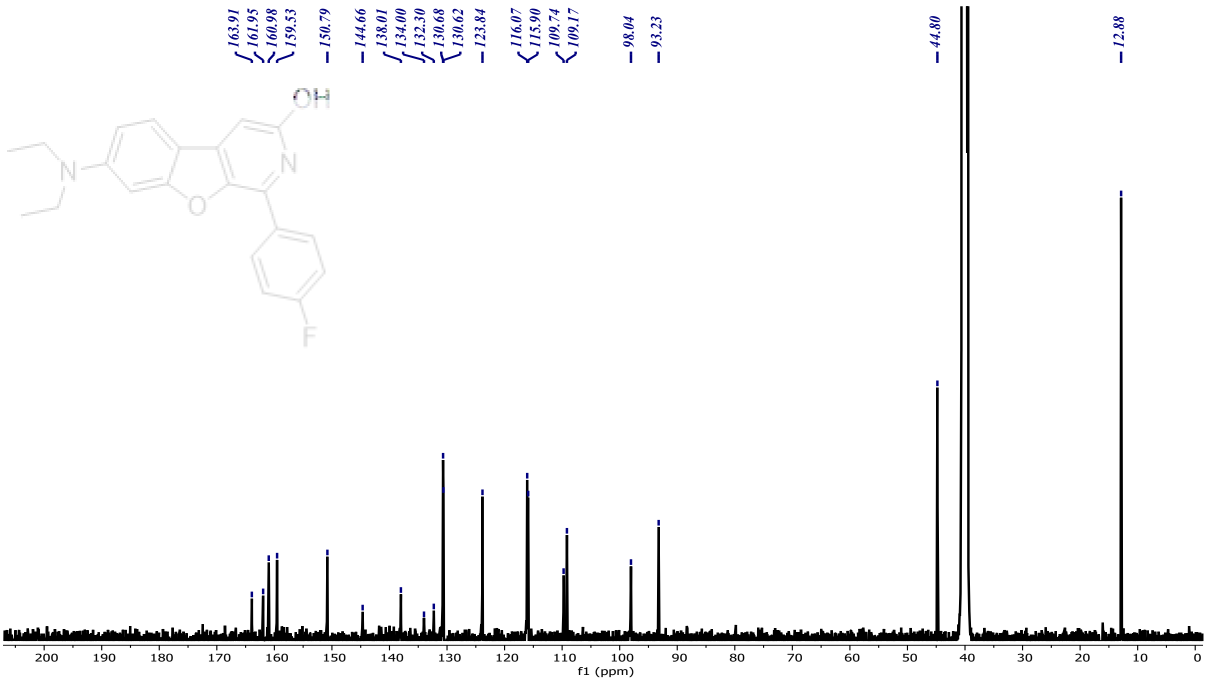

Figure 52. ^{13}C NMR (125 MHz, DMSO-d_6) spectrum of 7-(diethylamino)-1-(4-fluorophenyl)benzofuro[2,3-c]pyridin-3-ol (**9b**)

27. 7-(diethylamino)-1-(4-(piperidin-1-yl)phenyl)benzofuro[2,3-c]pyridin-3-ol (9c)

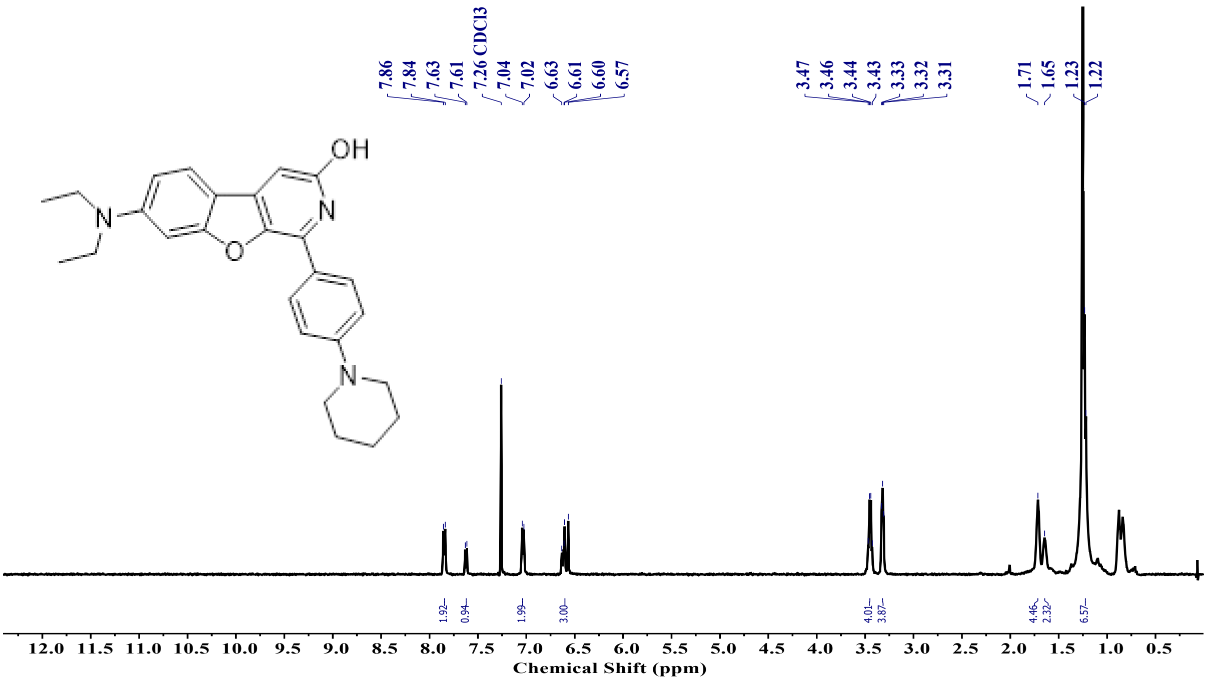

Figure 53. ^1H NMR (500 MHz, $CDCl_3$) spectrum of 7-(diethylamino)-1-(4-(piperidin-1-yl)phenyl) benzofuro -[2,3-c]pyridin-3-ol (**9c**)

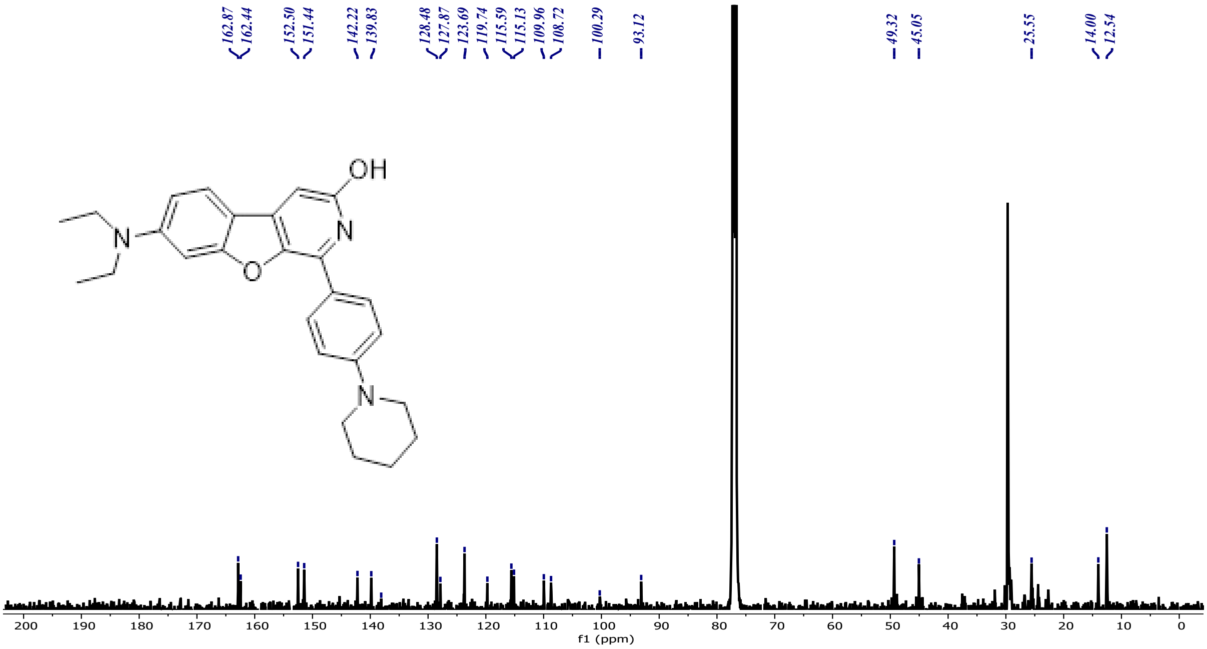

Figure 54. ^{13}C NMR (125 MHz, $CDCl_3$)spectrum of 7-(diethylamino)-1-(4-(piperidin-1-yl)phenyl) benzofuro -[2,3-c]pyridin-3-ol (**9c**)

Table 01 : Photophysical data of 6*J* in different solvents excitation at same λ_{abs} (386 nm).

Sr. no.	Solvent	λ_{exi} (nm)[a]	λ_{emi} (nm)[b]	Stokes shift (CM^{-1})
1	Chloroform ($CHCl_3$)[c]	386	437	3023
2	Tetrahydrofuran (THF)[c]	386	470	4630
3	Acetone	386	473	4765
4	1,4-Dioxane	386	470	4630
5	Dimethyl sulfoxide (DMSO)	386	488	5414
**6**	**Methanol (MeOH)**	**386**	**518**	6601
7	Ethanol (EtOH)	386	508	6221
8	2-Propanol	386	501	5946
9	1-Butanol	386	501	5946
10	1-Octanol	386	495	5704

[a]Excitation and [b]Emission recorded for **6*J***, 5 µM concentration in different solvents; at T = 25 °C; excitation and emission slit width of 5;5 nm, 600 V. [c]except for solvent $CHCl_3$ and THF at 570V.

Photoluminescence quantum yield (Φ_{PL}) measurement procedure.

The quinine sulphate in 0.1 M H_2SO_4 was cross-calibrated using the other two standards, Fluorescein in 0.1 M NaOH and 2-aminopyridine in 0.1 M H_2SO_4. This was achieved by calculating the quantum yield of each standard sample relative to the others. In this manner, the calculated Φ_{PL} of quinine sulphate[3] (reported Φ_{PL} of 0.54 at 360 nm in 0.1 M H_2SO_4), Fluorescein[4] (reported Φ_{PL} of 0.79 at 490 nm in 0.1 M NaOH) and 2-aminopyridine[5] (reported Φ_{PL} of 0.60 at 310 nm in 0.1 M H_2SO_4) are 0.534±0.04, 0.793±0.05 and 0.60±0.05, respectively. For quantum-yield measurement of test samples, the absorption and fluorescence spectra of BFPYOLs solution in DMSO were recorded in the following concentrations: 0 (blank), 1,2,3,4, and 5 µM. The integrated PL intensity and absorbance intensity were recorded and put in the equation (1) with quinine sulphate parameters as standard.

To calculate the relative quantum yield of all the test compounds, a single point method was used with reference quinine sulphate (reported Φ_{PL} of 0.54 at 360 nm in 0.1 M H_2SO_4). The following equation was used to calculate the quantum yield:

$$\mathbf{\Phi = \Phi_R \times I_s/I_R \times A_R/A_S \times \eta_s^2/ \eta_R^2} \quad \ldots\ldots\ldots\ldots(1)$$

where, Φ refers to the quantum yield of the sample, Φ_R refers to the quantum yield of reference, I_S & I_R being the measured integrated emission intensity (area under the curve), A_R & A_S refer to the absorbance of reference & sample, η is the refractive index. In order to minimise the reabsorption effects, absorbance intensity was kept below 0.1 at the excitation wavelength in the 10 mm fluorescence cuvette. An excitation and emission slit width of 5 nm at 600 V was used.

❖ **The molar absorption coefficient (ε) calculation for BFPYOLs in DMSO solvent.**

Molar absorption coefficient calculation for **6a**

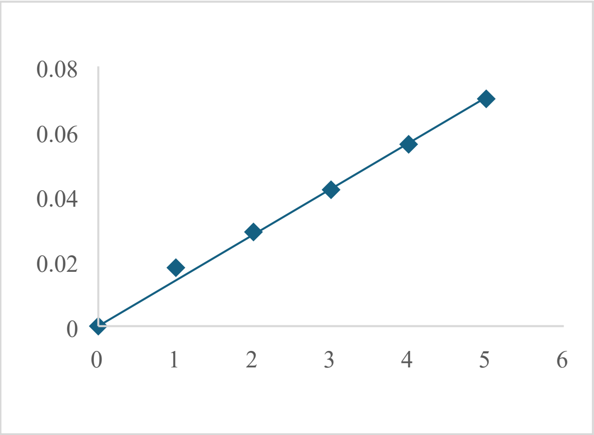

Molar absorption coefficient calculation for **6c**

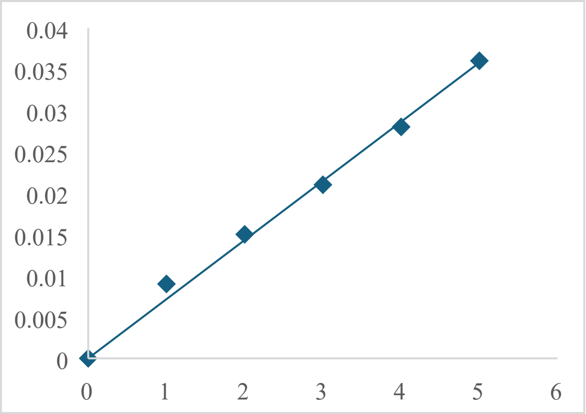

Molar absorption coefficient calculation for **6d**

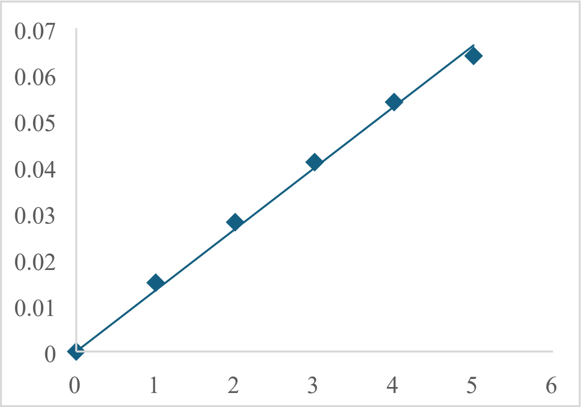

Molar absorption coefficient calculation for **6e**

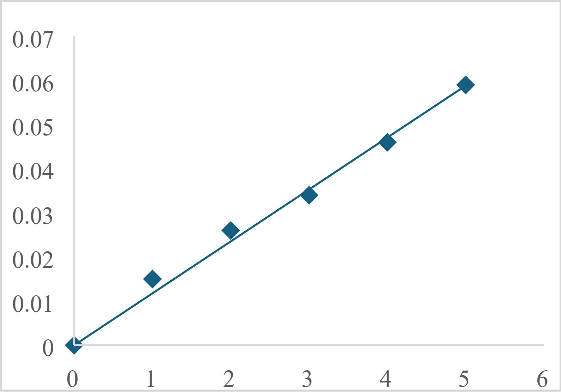

Molar absorption coefficient calculation for **6f**

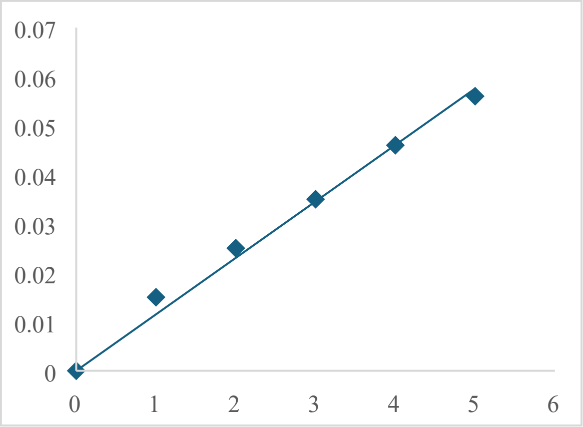

Molar absorption coefficient calculation for **6g**

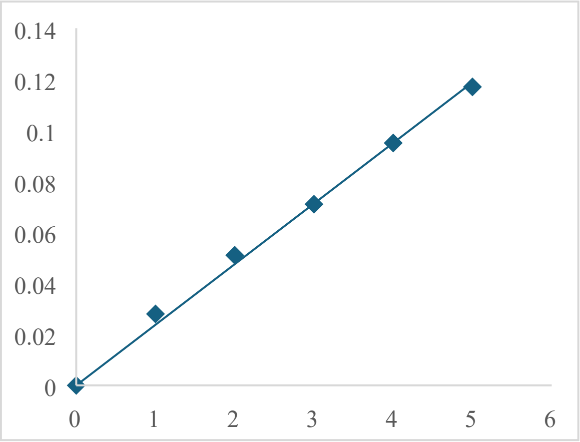

Molar absorption coefficient calculation for **6h**

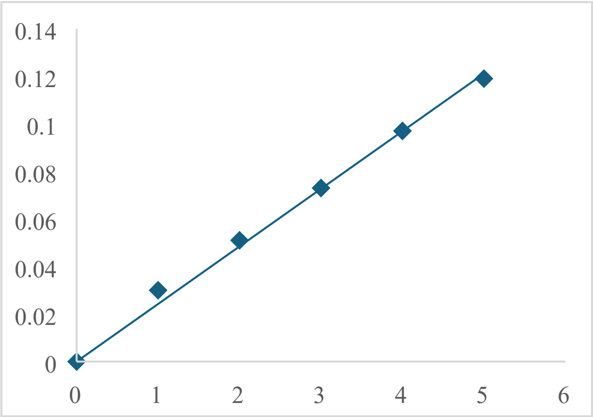

Molar absorption coefficient calculation for **6I**

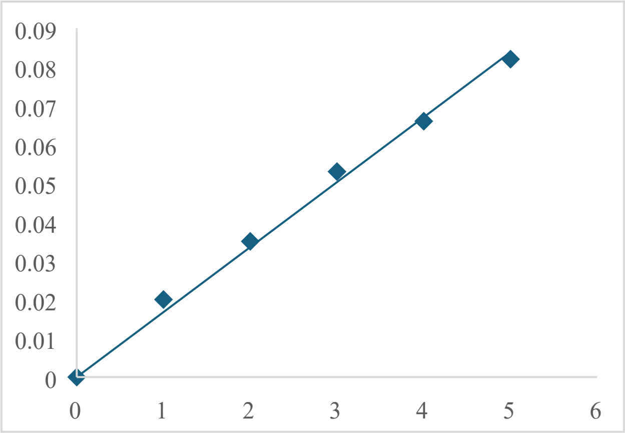

Molar absorption coefficient calculation for **6*J***

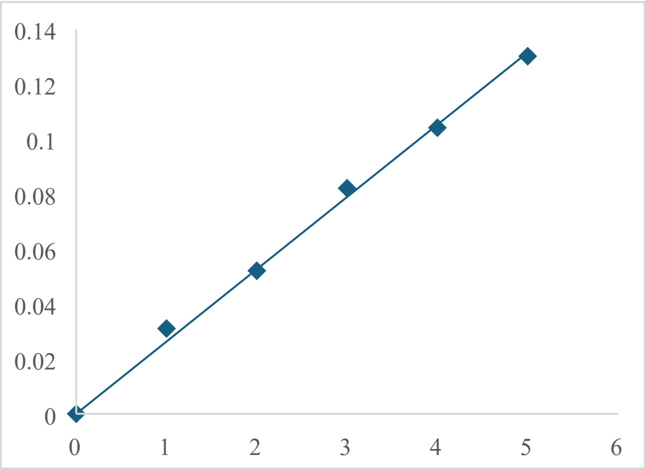

Molar absorption coefficient calculation for **6K**

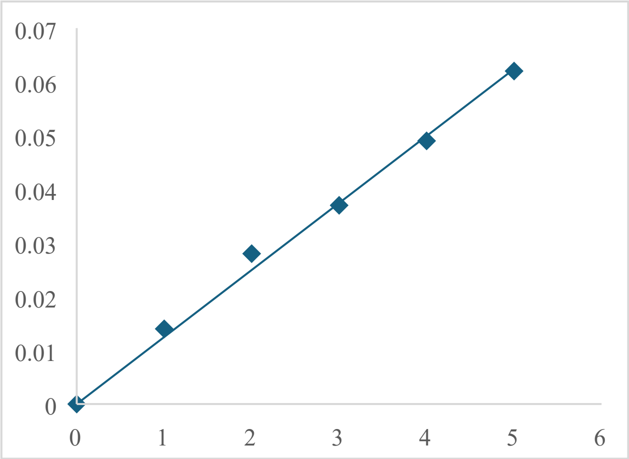

Molar absorption coefficient calculation for **6L**

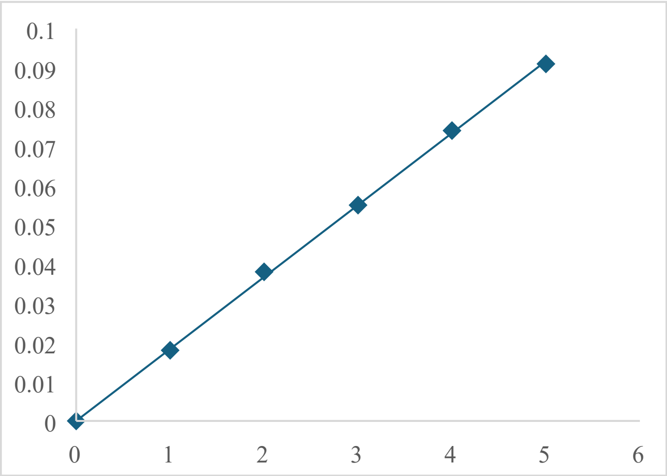

Molar absorption coefficient calculation for **6M**

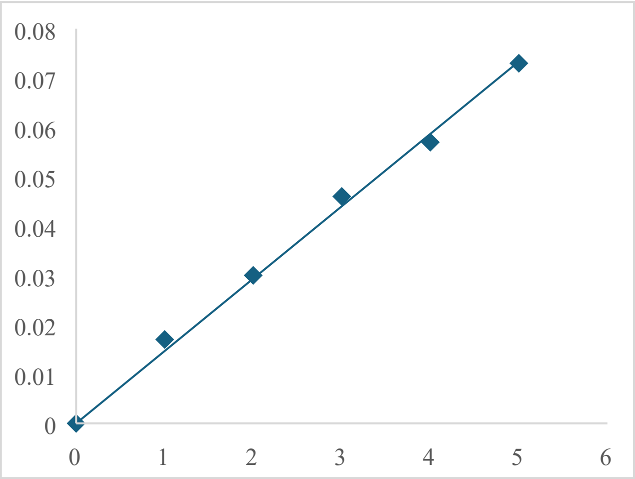

Molar absorption coefficient calculation for **7a**

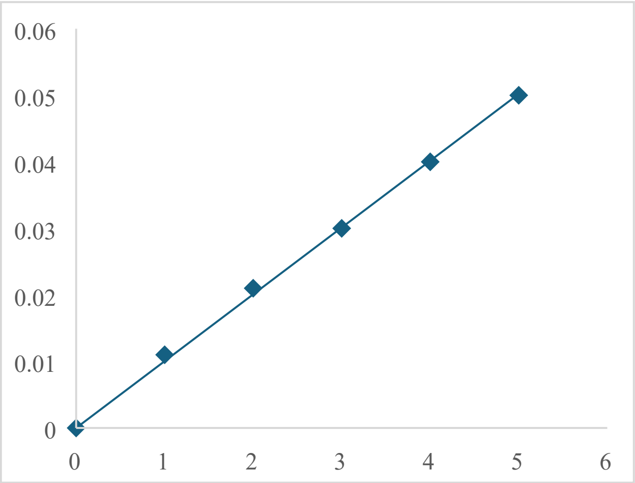

Molar absorption coefficient calculation for **7b**

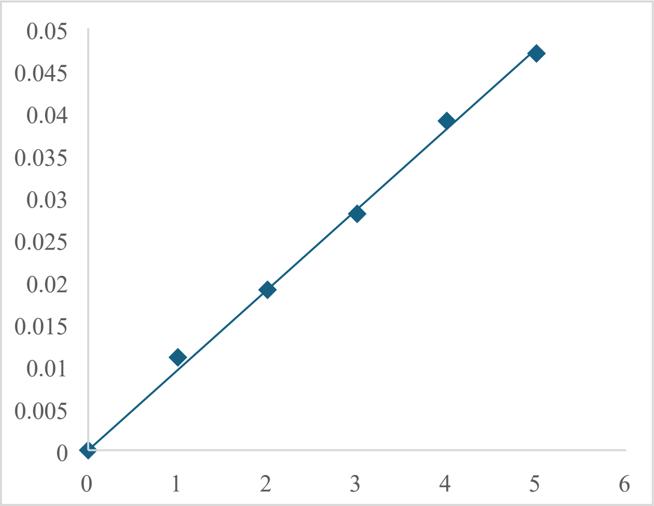

Molar absorption coefficient calculation for **7c**

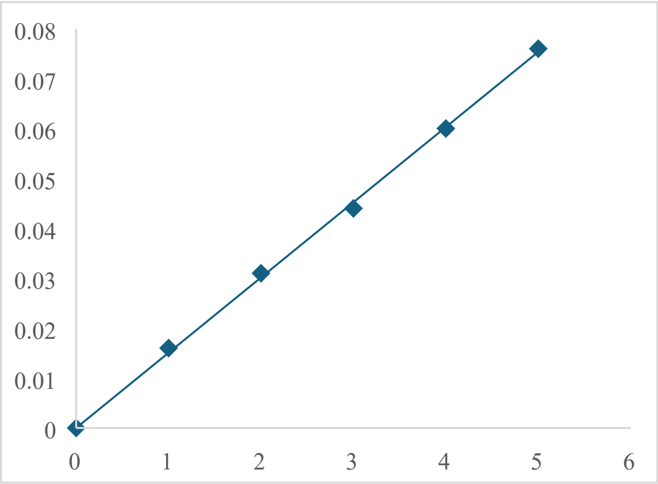

Molar absorption coefficient calculation for **7d**

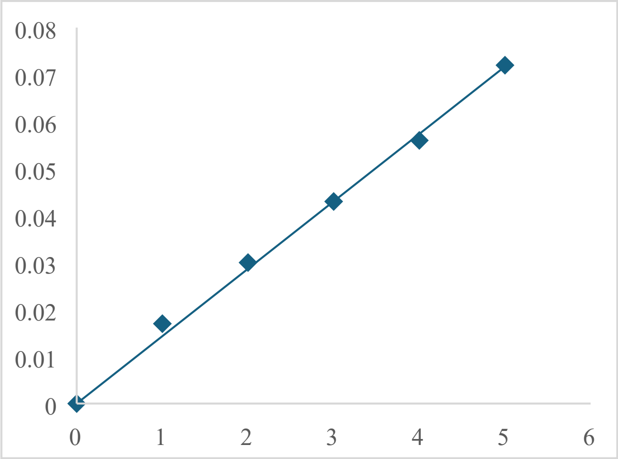

Molar absorption coefficient calculation for **7e**

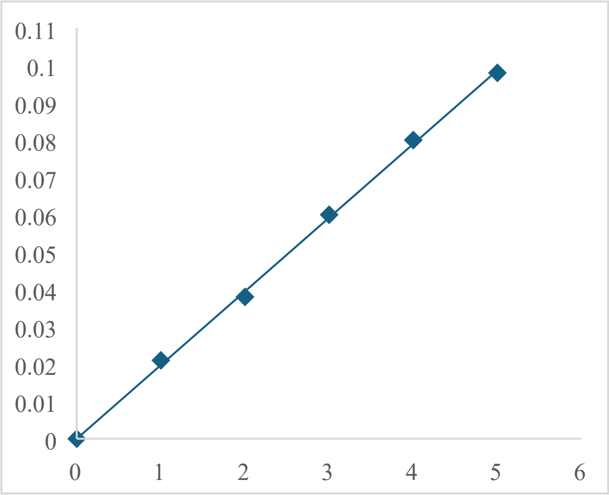

Molar absorption coefficient calculation for **7g**

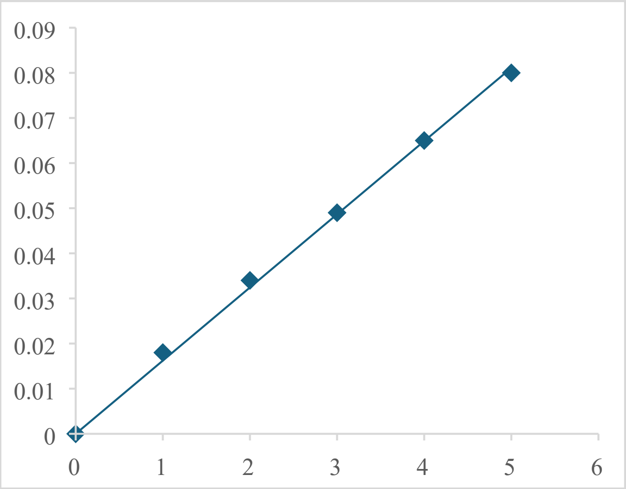

Molar absorption coefficient calculation for **7i**

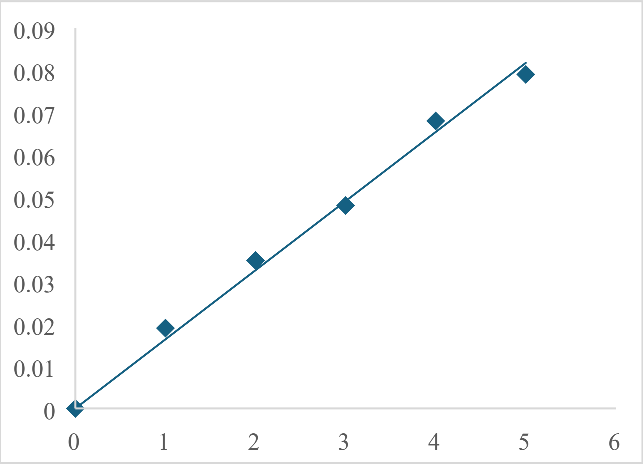

Molar absorption coefficient calculation for ***7J***

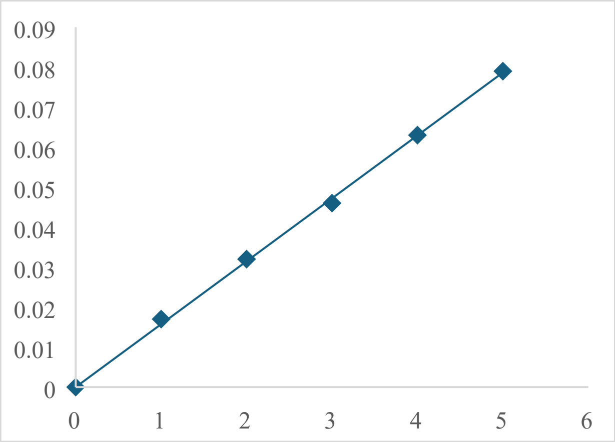

Molar absorption coefficient calculation for **7k**

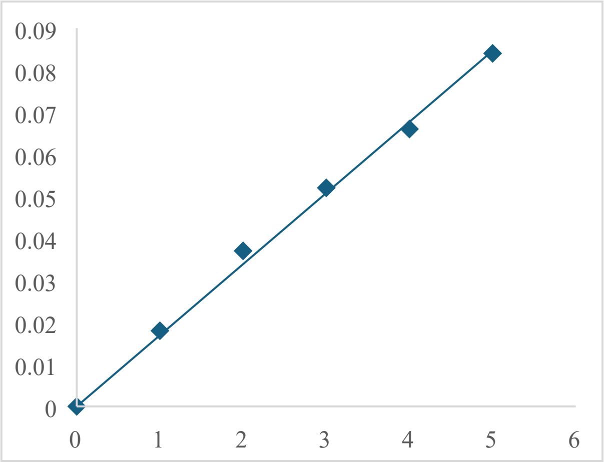

Molar absorption coefficient calculation for **8a**

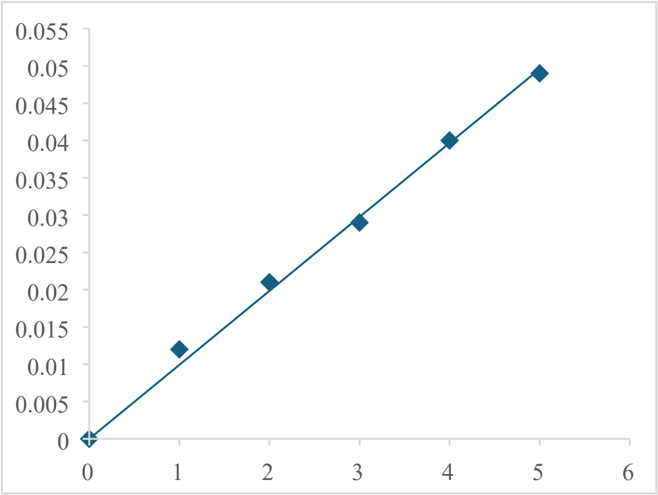

Molar absorption coefficient calculation for **9a**

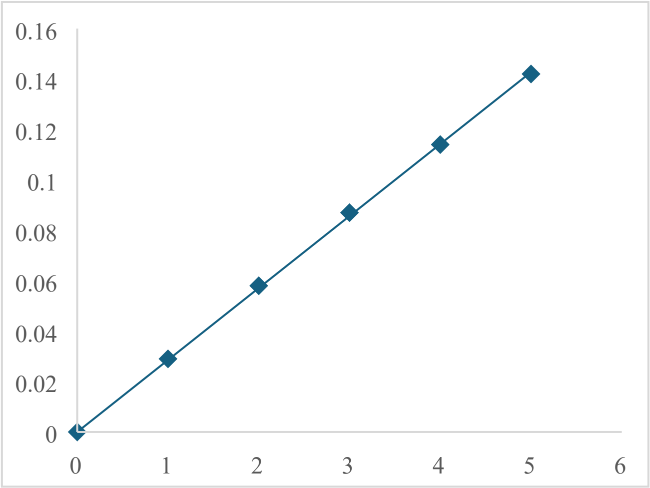

Molar absorption coefficient calculation for **9b**

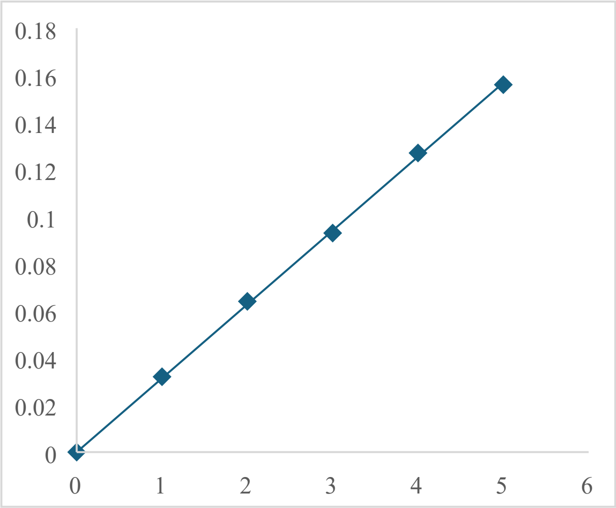

Molar absorption coefficient calculation for **9c**

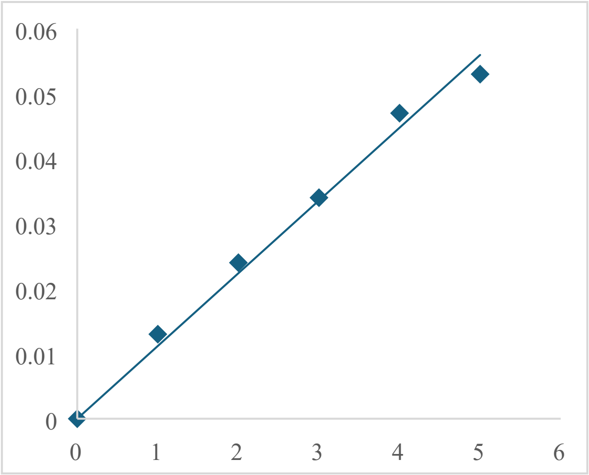

References:

1. C. Sandeep, S., K. Raunak, S. Harshada A PROCESS FOR PREPARATION OF BENZOFURAN AND BENZOFURO[2,3C]PYRIDINE COMPOUNDS. 552392, March 11, 2024, 2024.
2. Johnson, C. R.; Ansari, M. I.; Coop, A., Tetrabutylammonium Bromide-Promoted metal-free, efficient, rapid, and scalable synthesis of N-aryl amines. *ACS omega* **2018,** *3* (9), 10886-10890.

3. J.Y. Horiba, A Guide to Recording Fluorescence Quantum Yields, Stanmore, 2002, https://static.horiba.com/fileadmin/Horiba/Application/Materials/Material_Research/Quantum_Dots/quantumyieldstrad.pdf
4. Umberger, *J.* Q.; LaMer, V. K., The kinetics of diffusion controlled molecular and ionic reactions in solution as determined by measurements of the quenching of fluorescence1, 2. *Journal of the American Chemical Society* **1945,** *67* (7), 1099-1109.
5. Rusakowicz, R.; Testa, A., 2-Aminopyridine as a standard for low-wavelength spectrofluorimetry. *The Journal of Physical Chemistry* **1968,** *72* (7), 2680-2681.
